# Circ‐MALAT1 Functions as Both an mRNA Translation Brake and a microRNA Sponge to Promote Self‐Renewal of Hepatocellular Cancer Stem Cells

**DOI:** 10.1002/advs.201900949

**Published:** 2019-12-21

**Authors:** Liang Chen, Ruijiao Kong, Cong Wu, Shuo Wang, Zixin Liu, Shupeng Liu, Shuiping Li, Tian Chen, Chuanbin Mao, Shanrong Liu

**Affiliations:** ^1^ Department of Laboratory and Diagnosis Changhai Hospital Navy Medical University 168 Changhai Road Shanghai 200433 China; ^2^ No. 904 Hospital of the PLA Joint Logistics Support Force Wuxi 214000 China; ^3^ Shanghai Fourth People's Hospital Tongji University School of Medicine 1878 Sichuan North Road Shanghai 200081 China; ^4^ Department of Pathophysiology Shanghai Jiao Tong University School of Medicine Shanghai 200025 China; ^5^ Department of Chemistry and Biochemistry Stephenson Life Sciences Research Center University of Oklahoma 101 Stephenson Parkway Norman OK 73019‐5300 USA

**Keywords:** circular RNA, hepatocellular cancer stem cells, miR‐6887‐3p, PAX5, ribosomes, self‐renewal

## Abstract

Both circular RNAs (circRNAs) and cancer stem cells (CSCs) are separately known to be involved in cancer, but their interaction remains unclear. Here, the regulation of hepatocellular CSC self‐renewal is discovered by a circRNA, circ‐MALAT1, which is produced by back‐splicing of a long noncoding RNA, MALAT1. Circ‐MALAT1 is highly expressed in CSCs from clinical hepatocellular carcinoma samples under the mediation of an RNA‐binding protein, AUF1. Surprisingly, circMALAT1 functions as a brake in ribosomes to retard PAX5 mRNA translation and promote CSCs' self‐renewal by forming an unprecedented ternary complex with both ribosomes and mRNA. The discovered braking mechanism of a circRNA, termed mRNA braking, along with its more traditional role of miRNA sponging, uncovers a dual‐faceted pattern of circRNA‐mediated post‐transcriptional regulation for maintaining a specific cell state.

## Graphical abstract

Self‐renewal of hepatocellular cancer stem cells (CSCs) is found to be regulated by a circRNA, circ‐MALAT1, through a new mechanism termed mRNA braking. Circ‐MALAT1 functions as a brake in ribosomes to retard PAX5 mRNA translation and promote CSCs' self‐renewal by forming an unprecedented ternary complex with both ribosomes and mRNA.

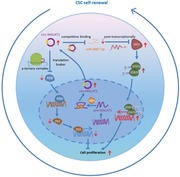

With the advancement of high‐throughput RNA sequencing (RNA‐seq) and novel computational approaches, recent studies have found that a large number of circular RNAs (circRNAs) are endogenous, conserved, stable and specific in eukaryotic cells.^1^, ^2^ CircRNAs are peculiarly stable RNAs produced by circularization of exons with or without introns through poorly characterized mechanisms.^3^ Emerging studies have revealed that the most common function of circRNAs is acting as miRNA sponges via binding sites and that changes of circRNAs abundance can regulate the activity of miRNAs on target genes.^2^, ^4^ Accordingly, the circRNA‐miRNA‐mRNA regulatory network might have potential implications for our understanding of diseases, especially cancers. In addition, recent studies reported that certain endogenous circRNAs are translatable.^5^, ^6^ Although circRNAs possess neither a 3' poly(A) tail nor a 5' cap, it has been reported that translation of circRNAs with an open reading frame (ORF) across the backspliced junction is driven by internal ribosome entry site (IRES) or m^6^A modification, in a cap‐independent manner.^5^, ^6^, ^7^, ^8^

Cancer stem cells (CSCs) were identified with self‐renewal and differentiation properties.^9^ They are considered as seeds for tumor occurrence, progression and metastasis. A higher proportion of CSCs results in tumors being more aggressive upon transition to mature carcinomas.^10^ In stark contrast to their differentiated progeny, CSCs have an unlimited self‐renewal potential, and metastasis‐initiating cells might be present within subpopulations of CSCs.^11^ In addition, the main clinical concern with CSCs is their strong resistance against therapy, a feature considered to be a primary cause of tumor recurrence.^12^ Clarifying the population and function of CSCs may help suppress cancer progression and improve therapeutic strategies.^13^ However, how CSCs renew and maintain themselves still remains unclear and even controversial.^14^, ^15^ Current studies indicated the potential role of circRNAs in cancer initiation, progression and metastasis.^16^, ^17^ Although both CSCs and circRNAs are separately related to the cancer initiation and progression, how circRNAs regulate CSCs in this context has not been known so far.

To answer this important question, we first isolated HCC primary cells from patients' solid tumors and obtained CSCs by suspension culture. Then we generated ribosomal‐depleted circRNA sequencing (circRNA‐seq) data from five pairs of HCC primary samples, compared circRNA expressions in the matched CSCs and adherent cells of HCC cell lines and HCC primary cells, and detected a number of circRNAs that were differentially expressed in the CSCs. One circRNA derived from lncRNA MALAT1, termed circ‐MALAT1 and numbered hsa_circ_0002082, formed a complex with both ribosomes and messenger RNA (mRNA) of a tumor suppressor gene (PAX5) by bearing two specific binding sites. The resultant unprecedented tertiary complex retards PAX5 mRNA translation, and ultimately contributes to the maintenance of CSCs. Circ‐MALAT1 was also found to activate the JAK2/STAT3 signaling pathway to promote CSC self‐renewal by acting as a miR‐6887‐3p sponge to enhance JAK2 expression. Our work reveals a dual‐faceted pattern of circRNA‐mediated post‐transcriptional regulation to maintain a specific state of a cell eventually.

To shed light on the circRNA expression between CSCs and cancer cells, we performed high throughput circRNA‐seq. For this purpose, we first isolated hepatocellular carcinoma (HCC) primary cells from patients' solid tumors and obtained CSCs by suspension culture^18^ (Figure S1, Supporting Information). CircRNA transcripts were then characterized by circRNA‐seq, which used ribosomal RNA (rRNA)‐depleted and RNase R‐digested RNA samples obtained from five pairs of HCC tumor tissues (five HCC adherent cells and five matched CSCs) for high‐throughput sequencing (Figure S2A, Supporting Information). Totally, 193 circRNA transcripts were aberrantly expressed in hepatocellular CSCs compared with adherent cells (**Figure** **1**A). Among the differentially expressed circRNAs, two were downregulated in CSCs samples of all five pairs and eighteen including circ‐MALAT1 were identified as ectopic expression in at least three of the five pairs (Figure S2B (Supporting Information) and Figure 1B).

**Figure 1:**
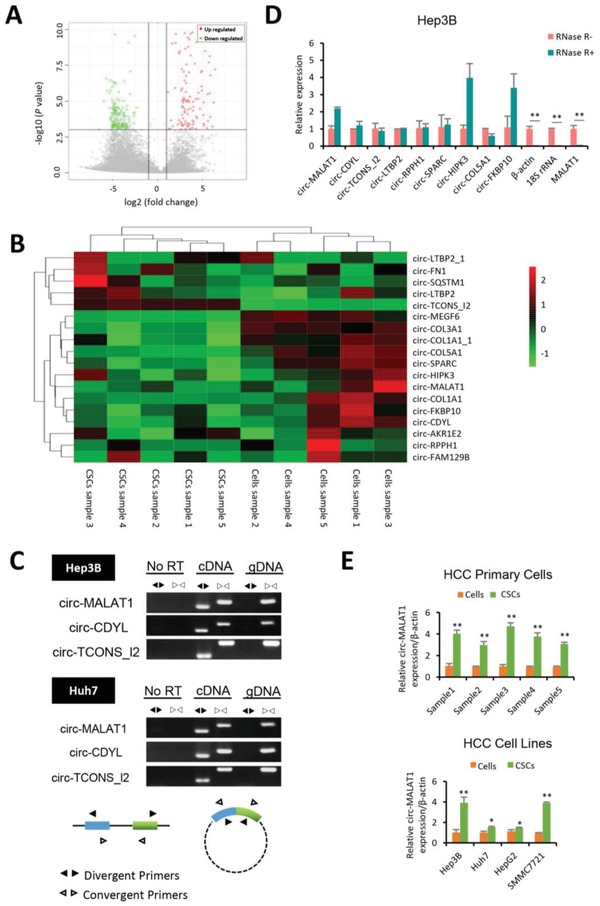
Circ‐MALAT1 is highly expressed in Hepatocellular CSCs. A) Volcano plot of differential circRNA expression between adherent cells and matched CSCs from five pairs of HCC primary samples. Points‐of‐interest (fold‐change > 2 or < 0.5 and *p* < 0.001) were represented in red (upregulated) and green (downregulated). Those points having no significant difference were shown in gray. B) Heat map of 18 differentially expressed circRNAs in at least three of the five pairs from circRNA‐seq data. Each column represented a sample of HCC adherent cells (Cells sample) or matched CSCs (CSCs sample), while each row represented an individual circRNA. The stripe color indicated the level of expression. C) Divergent primers amplified circR‐MALAT1, circ‐CDYL, and circ‐TCONS_I2 in cDNA but not genomic DNA (gDNA) or RNA (No RT). Convergent Primers could amplify linear RNA and circRNA in cDNA and parental gene in gDNA. D) CircRNAs were insensitive to RNase R treatment. Total RNA samples were split into two aliquots; one aliquot was treated with the RNase R exonuclease (RNase R+) and the other was subjected to a mock treatment (RNase R−). Linear RNAs such as β‐actin, 18S rRNA and MALAT1 were substantially digested with RNase R, while circRNAs were almost unaffected and even enriched (such as circ‐MALAT1, circ‐HIPK3 and circ‐FKBP10) due to degradation of linear RNAs. E) Circ‐MALAT1 expression was increased in CSCs of HCC primary cells (top panel) and cell lines (bottom panel). CSCs were enriched by the tumorsphere assay. Control primers, β‐actin. Columns, means from three independent experiments; bars, SD. ** *p* < 0.01, * *p* < 0.05.

Subsequently, based on the fold changes of differential expression among five pairs of samples from high‐throughput circRNA‐seq and our preidentification of the expression of the eighteen circRNAs in HCC cells (data not shown), the ectopic expressions of nine out of the eighteen circRNAs were further assessed. Because divergent primers could amplify circRNAs only in cDNA instead of in genomic DNA, but convergent primers could generate target bands in both genomic DNA and cDNA, we used both convergent and divergent primers to collectively confirm that our discovered circRNAs were indeed circular RNAs with the head‐to‐tail splicing (Figure 1C, Figure S2C and Table S9, Supporting Information). In addition, because the head‐to‐tail splicing could also be formed through either genomic rearrangements or trans‐splicing and detected by divergent primers, we further employed quantitative reverse transcription polymerase chain reaction (qRT‐PCR) to confirm that the nine candidate circRNAs were resistant to RNase R (Figure 1D), further verifying the circular closed structure of the circRNAs and ruling out the possibility of the trans‐splicing and genomic rearrangements. Among these nine candidates, circ‐MALAT1 (circBase ID: hsa_circ_0002082) presented a significantly higher expression level in CSCs than in matched adherent cells both in HCC primary cells and in cell lines, and thus selected for further study (Figure 1E and Figure S2D, Supporting Information).

We next explored the underlying mechanism of circ‐MALAT1 biogenesis in hepatocellular CSCs. To this end, we first used the web tool CircInteractome (https://circinteractome.nia.nih.gov) to identify splicing‐associated factors bound with flanking regions of circ‐MALAT1 (Table S1, Supporting Information). We analyzed the expressions of associated proteins between CSCs and adherent cells. Among the top three candidate RNA binding proteins, the AUF1 mRNA expression was upregulated by more than two folds in CSCs, while AGO2 and FUS were not changed in abundance (**Figure** **2**A, top panel). Notably, AUF1 protein expression was significantly increased in hepatocellular CSCs by western blot assay (Figure 2A, bottom panel and Figure S3A, Supporting Information). Both RNA‐binding Protein Immunoprecipitation (RIP) assay using AUF1 antibody and qRT‐PCR analysis indicated that AUF1 protein bound pre‐circ‐MALAT1 (Figure 2B). To further determine the role of AUF1 in the biogenesis of circ‐MALAT1, we silenced AUF1 in the Huh7 cells using small interfering RNA (siRNA) and found that AUF1 knockdown significantly reduced the expression of circ‐MALAT1 (Figure 2C).

**Figure 2:**
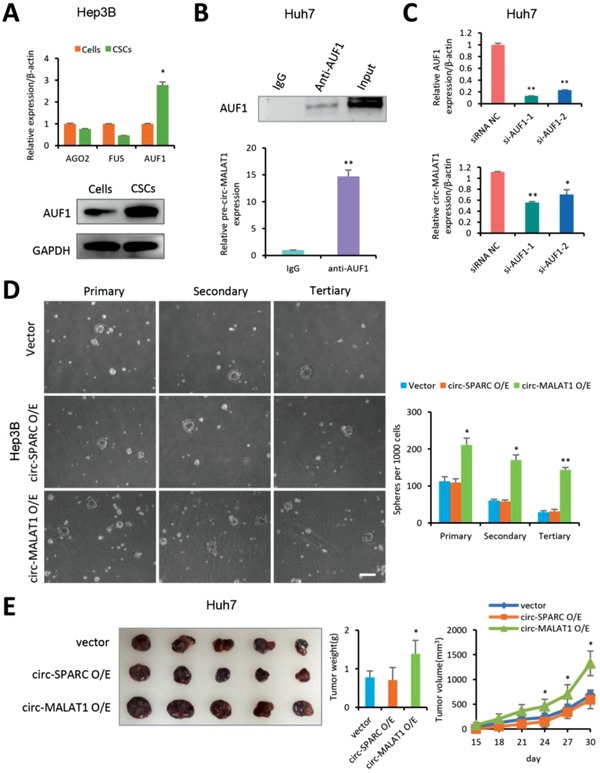
Circ‐MALAT1 promotes the self‐renewal of hepatocellular CSCs. A) Top panel—mRNA of RNA‐binding protein AUF1 (not AGO2 or FUS) was upregulated in Hep3B CSCs. Control primers, β‐actin. Bottom panel, AUF1 protein was analyzed in Hep3B cells and CSCs by western blot assay. CSCs were enriched by the tumorsphere assay. B) RIP was performed using Huh7 cell lysate and either anti‐AUF1 or IgG as IP antibody. Top panel, AUF1 antibody was confirmed by western blot. AUF1 protein was observed in the anti‐AUF1 RIP (lane 2) and 10% Input (lane 3) but not in the IgG RIP (Lane1). Bottom panel, purified RNA from RIP was analyzed by qRT‐PCR using primers specific for the pre‐circ‐MALAT1. C) Top panel, AUF1 was silenced in Huh7 cells by two independent siRNAs (si‐AUF1‐1 and si‐AUF1‐2). Bottom panel, circ‐MALAT1 expression was decreased after silencing AUF1 in Huh7 cells. Control primers, β‐actin. D) Left panel, bright images of spheres of circ‐MALAT1 overexpressed (circ‐MALAT1 O/E), circ‐SPARC overexpressed (circ‐SPARC O/E), or vector control (Vector) Hep3B cells. Right panel, quantitative statistics of the numbers of the primary, secondary, and tertiary spheres per 1000 cells. Scale bar, 200 µm. E) Both Circ‐MALAT1 overexpressed (circ‐MALAT1 O/E), circ‐SPARC overexpressed (circ‐SPARC O/E), and vector control (Vector) Huh7 cells were diluted and subcutaneously implanted into five BALB/c nude mice, respectively. Tumors were observed every 3 d for a period of one month 14 d post‐tumor inoculation. Similar results were obtained in three to six independent experiments. ** *p* < 0.01, * *p* < 0.05.

To uncover the role of circ‐MALAT1 in hepatocellular CSCs, a circRNA‐specific overexpression plasmid (Figure S3B, Supporting Information) was constructed. Moreover, circ‐SPARC (circBase ID: hsa_circ_0004104), which presented no significant difference between CSCs and matched adherent cells either in HCC primary cells or in cell lines (Figure S2D, Supporting Information), was selected as a negative control. Specific overexpression of circ‐MALAT1 and circ‐SPARC was confirmed by qRT‐PCR with divergent primers (Figure S3C and Table S9, Supporting Information). No notable effect on the cell proliferation was detected after circ‐MALAT1 or circ‐SPARC overexpression in Hep3B and Huh7 cells by Cell Counting Kit‐8 assay (Figure S3D, Supporting Information) and EdU imaging analysis (Figure S3E, Supporting Information). Additionally, either cell cycle progression or transwell invasion assays displayed no significant changes in both Hep3B and Huh7 cells overexpressing circ‐MALAT1 or circ‐SPARC (Figure S3F,G, Supporting Information). Then stably circRNA‐overexpressed HCC cells were established and plated for tumorsphere assays. Functionally, circ‐MALAT1 overexpressed cells formed more primary, secondary, and tertiary suspension spheres than the empty control group, while no significant difference was observed between cells overexpressing circ‐SPARC and empty vector (Figure 2D). More importantly, we evaluated the growth of xenograft tumor from the stably circ‐MALAT1‐overexpressed Huh7 cells. In vivo injection of circ‐MALAT1 overexpressed cells (circ‐MALAT1 O/E) led to a larger tumor size (Figure 2E, left panel), a higher tumor weight (Figure 2E, middle panel) and a faster tumor growth rate (Figure 2E, right panel) than that of control cells (Vector and circ‐SPARC O/E). Above all, these findings suggested that circ‐MALAT1 promoted the CSCs self‐renewal in HCC under the mediation of AUF1.

To understand the mechanism by which circ‐MALAT1 regulates hepatocellular CSCs self‐renewal, we focused on the circ‐MALAT1‐associated downstream proteins. Antibody‐array‐based analysis of 1358 human signaling explorer antibodies was performed (Table S2, Supporting Information). Proteins were prepared from Huh7 cells transfected with circ‐MALAT1 plasmid or empty vector. After the ratio of comparison was set at ≥1.8 or ≤1/1.8, 224 and 43 proteins were recognized as upregulated and downregulated, respectively, with the circ‐MALAT1 overexpression (**Figure** **3**A and Table S3, Supporting Information). Based on Gene Ontology (GO) analysis, 96 of the 224 upregulated proteins were found to be associated with CSCs self‐renewal or tumor metastasis and proliferation (Figure 3B, left panel and Table S4, Supporting Information). Among the three GO terms with the most significant fold enrichment, five proteins (FGF2, TGFB1, PTK2, JAK2, and GH1) with the highest fold changes were selected as the further subjects. In addition, 10 of the 43 downregulated proteins were involved in tumor suppression^19^, ^20^, ^21^, ^22^, ^23^, ^24^, ^25^, ^26^, ^27^, ^28^ and were thus selected for the subsequent analysis (Figure 3B, right panel and Table S5, Supporting Information).

**Figure 3:**
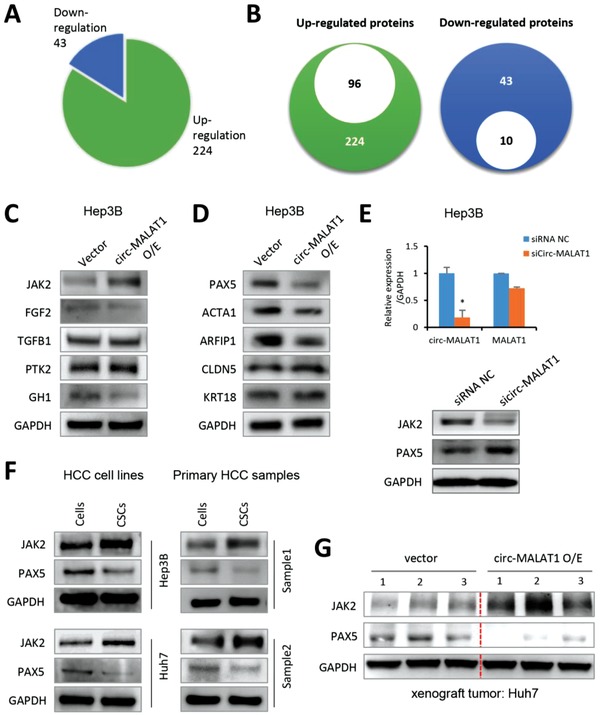
Circ‐MALAT1 expression results in JAK2 and PAX5 Differential Expression. A) Pie chart showed that 224 proteins were upregulated and 43 were downregulated from protein microarray. Ratio of comparison at ≥1.8 or ≤1/1.8. B) Left panel, of the 224 upregulated proteins, 96 proteins were associated with CSCs self‐renewal or tumor metastasis and proliferation. Right panel, 10 of the 43 downregulated proteins were associated with tumor suppression. C) Five upregulated proteins from antibody‐array results were analyzed in Hep3B cells transfected with circ‐MALAT1 overexpression (circ‐MALAT1 O/E) or empty vector (Vector) by western blot analysis. D) Five downregulated proteins from antibody‐array results were analyzed in circ‐MALAT1 overexpression (circ‐MALAT1 O/E) or empty vector (Vector) cells by western blot analysis. E) Top panel, expression of circ‐MALAT1 and MALAT1 was analyzed by qRT‐PCR in Hep3B cells transfected with sicirc‐MALAT1 and siRNA NC. Bottom panel, protein expression of JAK2 and PAX5 was detected by western blot in circ‐MALAT1‐silenced‐Hep3B cells. siRNA NC, scramble control siRNA; sicirc‐MALAT1, siRNA against circ‐MALAT1. Columns, means from three independent experiments; bars, SD. * *p* < 0.05. F) Expression of JAK2 and PAX5 of CSCs and adherent cells of two HCC cell lines (left panel) Hep3B and Huh7 and two HCC primary cells (right panel) were detected by western blot. CSCs were enriched by the tumorsphere assay. G) JAK2 and PAX5 expressions were determined in the xenograft tumors by western blot.

Protein samples were then prepared from Hep3B cells with circ‐MALAT1 overexpression or empty vector and analyzed by western blot to verify the antibody‐array results. The most significantly increased protein correlated with circ‐MALAT1 overexpression was found to be Janus‐activated kinase 2 (JAK2), a well‐characterized protein that could induce CSC‐like characteristics (Figure 3C).^29^, ^30^, ^31^ Conversely, Paired box 5 (PAX5), a tumor suppressor, decreased most significantly after circ‐MALAT1 overexpression among the 5 proteins (PAX5, ACTA1, ARFIP1, CLDN5, and KRT18) out of the 10 downregulated proteins in HCC cells (Figure 3D). Additionally, we silenced circ‐MALAT1 using a specific siRNA (Figure 3E, top panel and Table S10, Supporting Information). Consistent with the overexpression study, circ‐MALAT1 knockdown remarkably downregulated JAK2 protein expression and upregulated PAX5 protein expression (Figure 3E, bottom panel). Subsequently, we examined the expression of JAK2 and PAX5 in CSCs and adherent cells of HCC cell lines and primary cells by western blot. JAK2 expression was much upregulated in hepatocellular CSCs, while PAX5 was downregulated in CSCs (Figure 3F). Moreover, xenograft tumors generated by circ‐MALAT1‐overexpressing Huh7 cells presented higher JAK2 and lower PAX5 protein expression (Figure 3G). Collectively, these results strongly indicated that circ‐MALAT1 could lead to JAK2 upregulation and PAX5 downregulation in hepatocellular CSCs.

So far, the reported studies only show that circRNA upregulates the protein expression. In contrast to this previous view, here we found that the PAX5 protein expression was downregulated by circ‐MALAT1 overexpression. To unlock our discovered downregulation mechanism, we first performed fluorescence in situ hybridization and found that circ‐MALAT1 was localized exclusively in the cytoplasm rather than in the nucleus (**Figure** **4**A and Figure S4A, Supporting Information). Then we detected the expression of PAX5 mRNA following circ‐MALAT1 overexpression by qRT‐PCR. Surprisingly, no significant change was observed with circ‐MALAT1 overexpression (Figure 4B), which indicated that circ‐MALAT1 might exert its regulatory effect on PAX5 at the post‐transcriptional level. Further analysis was performed by the circRNADb (https://202.195.183.4:8000/circrnadb/circRNADb.php) and ORFfinder (https://www.ncbi.nlm.nih.gov/orffinder/) databases. Two IRESs were observed in the circ‐MALAT1 sequence, suggesting the potential combining capacity of circ‐MALAT1 with ribosomes. However, no open reading frame (ORF) was found in circ‐MALAT1, meaning circ‐MALAT1 did not present an encoding capacity (Figure S4B, Supporting Information). Meanwhile, we found there were 11 bases (557–567) complementary to the PAX5 coding sequence (1295–1305) in circ‐MALAT1 through NCBI Blast. Based upon these analyses, we hypothesized that circ‐MALAT1 might obstruct the translation of PAX5 by forming a ternary complex with PAX5 and ribosomes via IRESs and 11 complementary bases, respectively (Figure 4C).

**Figure 4:**
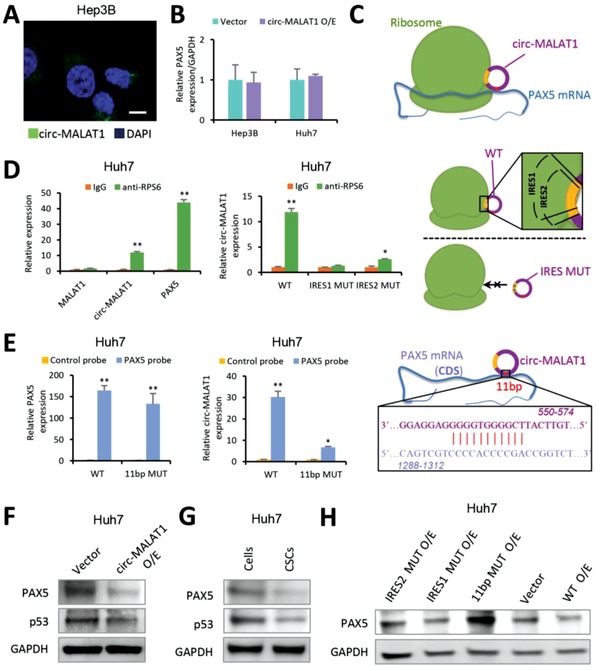
Circ‐MALAT1 obstructs PAX5 translation by binding to PAX5 coding sequence and ribosomes. A) RNA fluorescence in situ hybridization for circ‐MALAT1. Nuclei were stained with DAPI. Green staining signal against circ‐MALAT1 was localized in cytoplasm. Scale bar, 10 µm. B) The level of PAX5 mRNA was analyzed by qRT‐PCR. Circ‐MALAT1 overexpression (circ‐MALAT1 O/E) led to no significant difference in PAX5 mRNA level compared to the control (Vector). Control primers, GAPDH. C) An interaction model showing that circ‐MALAT1 recognized PAX5 mRNA via 11 bp bases paring (red site) and competitively inhibited PAX5 translation through IRESs (yellow sites). D) Left panel, RIP lysates prepared from circ‐MALAT1 overexpressed Huh7 cells were subjected to immunoprecipitation using either a normal rabbit IgG or anti‐RPS6 antibody. Purified RNA was analyzed by qRT‐PCR using primers specific for MALAT1, circ‐MALAT1 and PAX5, respectively. Middle panel, RIP was performed using lysates prepared from Huh7 cells overexpressing wild type (WT), IRES1‐mutated (IRES1 MUT) and IRES2‐mutated (IRES2 MUT) circ‐MALAT1, respectively. Purified RNA was analyzed by qRT‐PCR using primers specific for circ‐MALAT1. Right panel, the model above the dashed line (indicating the result in the left panel of (D) showed that circ‐MALAT1 with wild type of IRES (WT, “1” and “2” overlapped yellow sites) could bind to ribosome while the model below the dashed line (indicating the result in the middle panel of (D) showed that circ‐MALAT1 with mutated IRES (MUT) could not bind to ribosome. E) In vivo RNA pull‐down using PAX5 specific probes was performed in circ‐MALAT1 WT or 11 bp MUT overexpressed Huh7 cells, followed by qRT‐PCR to detect PAX5 (left panel) and circ‐MALAT1 (middle panel). Right panel, the interaction model between circ‐MALAT1 and the CDS region of PAX5 mRNA via 11 bp bases paring (red site). F) PAX5 and its downstream protein p53 were detected by western blot. Vector, empty vector overexpression; circ‐MALAT1 O/E, circ‐MALAT1 overexpression. G) PAX5 and p53 were analyzed in CSCs and adherent cells of Hep3B cell line by western blot. CSCs were enriched by the tumorsphere assay. H) PAX5 was detected in Huh7 cells overexpressing circ‐MALAT1 wild (WT O/E) or mutant type (11 bp MUT O/E, IRES1 MUT O/E, and IRES2 MUT O/E) or none (Vector) by western blot. Columns, means from three independent experiments; bars, SD. ** *p* < 0.01, * *p* < 0.05.

To verify this model, RIP assay was performed using circ‐MALAT1 overexpressed Huh7 cell lysate. We used either normal rabbit IgG or ribosomal protein S6 antibody (anti‐RPS6) as an antibody for immunoprecipitation to identify specific RNA molecules associated with ribosomes. The RPS6 antibody was confirmed by western blot prior to RIP analysis (Figure S4C, Supporting Information). Compared with control IgG, approximately a 12‐fold increase of circ‐MALAT1 and a 40‐fold increase of PAX5 were found in PCR product from anti‐RPS6 RIP. However, significant difference was not found in the group of MALAT1 (< twofold) (Figure 4D, left panel and Figure S4D, Supporting Information), indicating that both circ‐MALAT1 and PAX5 could be combined with ribosomes. Next, we constructed two circ‐MALAT1 mutation plasmids on which position 640–762 (IRES1) or position 611–755 (IRES2) of circ‐MALAT1 was replaced with the non‐IRES parts, respectively (Figure S4E, Supporting Information). Huh7 cells transfected with circ‐MALAT1 IRES‐mutated plasmids were lysed for RIP assay. Anti‐RPS6 RIP product from circ‐MALAT1 IRES2‐mutated samples had a two‐ to threefold increase in the quantity compared to IgG control while IRES1 mutation had no difference (Figure 4D, middle panel), suggesting that the consensus sequence of IRES1 and IRES2 (position 640–755) was required for circ‐MALAT1 to bind to ribosomes.

In order to fish out the interaction between PAX5 and circ‐MALAT1 in HCC cells, PAX5 mRNA was detected by qRT‐PCR in the purified circ‐MALAT1‐associated RNA via in vivo circ‐MALAT1 pull‐down assay. We found a significant enrichment of circ‐MALAT1 as well as PAX5 mRNA by circ‐MALAT1 specific probes as compared to the controls (Figure S4F, Supporting Information). Meanwhile, in vivo RNA pull‐down experiment with biotin‐labeled PAX5 mRNA specific probes was also performed in Huh7 cells transfected with circ‐MALAT1, either wild type (WT) or the mutant with the deletion of 9 (out of 11) bases that are complementary to PAX5 coding sequence (11 bp MUT) (Figure S4G, Supporting Information). We found a specific enrichment of PAX5 mRNA at a comparable level in both circ‐MALAT1 WT and 11 bp MUT overexpression in the Huh7 samples as compared to the controls (Figure 4E, left panel). However, the enrichment multiples of circ‐MALAT1 in the mutant samples (approximately fivefold) were far below those in WT (≈30‐fold) (Figure 4E, middle panel), suggesting that the 11 bases were of great importance for circ‐MALAT1 to combine PAX5 mRNA (Figure 4E, right panel).

We then detected the expression of candidate target proteins. Western blot showed PAX5 protein and its downstream protein p53 were downregulated in circ‐MALAT1 overexpressed cells (Figure 4F). Meanwhile, PAX5 and p53 were also confirmed to be downregulated in the CSCs (Figure 4G). However, when we mutated either the 11 complementary bases or IRES1 on circ‐MALAT1, no significant decrease of PAX5 protein was observed in circ‐MALAT1‐mutant overexpressed cells (Figure 4H). Only the cells transfected with WT circ‐MALAT1 presented significant downregulation of PAX5 protein, but those transfected with circ‐MALAT1 IRES2‐mutant showed slight downregulation of the PAX5 protein (Figure 4H). The overexpression level of circ‐MALAT1, either wild or mutant type, was detected by qRT‐PCR (Figure S4H, Supporting Information). Next, through RNA interfering, we silenced circ‐MALAT1 or/and PAX5 in Huh7 cells (Figure S4I, left panel, Supporting Information). The data showed that silencing circ‐MALAT1 enhanced the protein levels of PAX5 and subsequently p53, whereas the enhancement could be rescued with PAX5 knockdown (Figure S4I, right panel, Supporting Information). Together, the above results indicated that circ‐MALAT1 competitively bound ribosomes via IRESs and thereby suppressed PAX5 translation.

As JAK2 increase was observed with the up‐regulation of circ‐MALAT1 in liver CSCs, and circRNAs are known to function as microRNA (miRNA) sponges to upregulate target gene expression,^4^, ^17^ we then used MiRanda Program to predict the miRNA binding sites on the circ‐MALAT1 sequence. Bioinformatics analysis indicated that there were 322 potential miRNAs that could be bound by circ‐MALAT1 (Figure S5A and Table S6, Supporting Information).

We also used the TargetScanHuman (https://www.targetscan.org/vert_72/) to identify the miRNAs potentially targeting JAK2 (Table S7, Supporting Information). By overlapping the two groups of miRNAs, 121 predicted miRNAs were screened out because they had binding sites with circ‐MALAT1 and potentially targeted JAK2 (Figure S5B and Table S8, Supporting Information). The top 5 miRNAs with the highest binding free energy between miRNAs and circ‐MALAT1 were then analyzed. With circ‐MALAT1 overexpression, miR‐6887‐3p expression was greatly reduced while the expression of miR‐214‐3p, miR‐676‐5p, miR‐503‐5p, and miR‐4773 exhibited no significant change (**Figure** **5**A). Further detailed analysis showed that circ‐MALAT1 harbored eight conserved miR‐6887‐3p seed matches (Figure S5C, Supporting Information). In vivo circ‐MALAT1 pull‐down experiment showed that circ‐MALAT1 specific probes could efficiently pull down circ‐MALAT1 as compared to the control (Figure 5B, left panel), and miR‐6887‐3p was also significantly enriched (Figure 5B, right panel). This result suggested that miR‐6887‐3p was a circ‐MALAT1‐associated miRNA in HCC cells.

**Figure 5:**
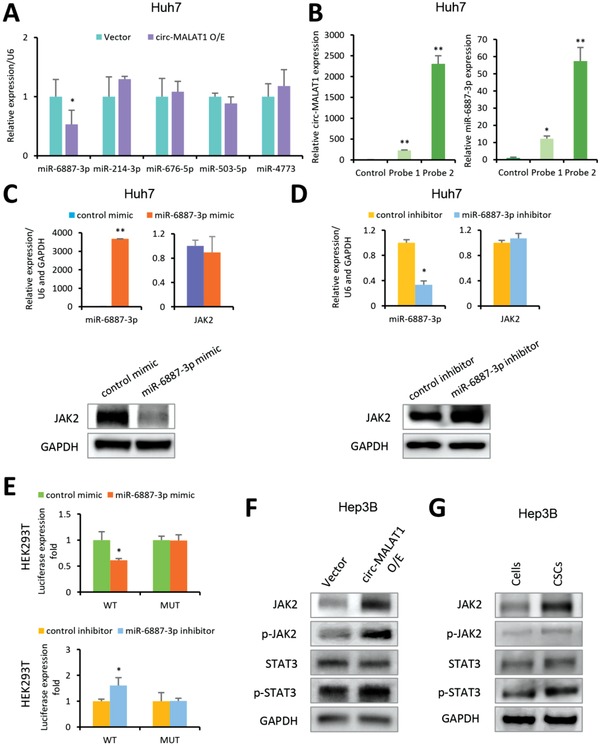
Circ‐MALAT1 acts as miR‐6887‐3p sponge to up‐regulate JAK2. A) Top five miRNAs with the highest binding free energy were analyzed in circ‐MALAT1 overexpressed (circ‐MALAT1 O/E) or empty vector (Vector) Huh7 cells by qRT‐PCR. Control primers, U6. B) In vivo circ‐MALAT1 pull‐down using circ‐MALAT1 specific probes (Probe 1 and Probe 2) was performed in circ‐MALAT1 overexpressed Huh7 cells, followed by qRT‐PCR to detect circ‐MALAT1 (left panel) and miR‐6887‐3p (right panel). C) After treatment with miR‐6887‐3p mimic or its scrambled version (control mimic), both miR‐6887‐3p level and JAK2 expression at mRNA were detected by qRT‐PCR (top panel), and JAK2 expression at protein level was detected by western blot (bottom panel), respectively. U6 and GAPDH were used as control primers. D) After treatment with the miR‐6887‐3p inhibitor or its scrambled version (the control inhibitor), the level of both the miR‐6887‐3p and JAK2 mRNA was detected by qRT‐PCR (top panel), and JAK2 expression at the protein level was detected by western blot (bottom panel). U6 and GAPDH were used as control primers. E) The luciferase activities of pMIR‐JAK2‐3'UTR wild type (WT) and mutated type (MUT) were detected when cells were cotransfected with miR‐6887‐3p mimic (top panel) or miR‐6887‐3p inhibitor (bottom panel). F) The key molecules of JAK2/STAT3 pathway were analyzed in circ‐MALAT1 overexpressed (circ‐MALAT1 O/E) or empty vector (Vector) cells by western blot. p‐JAK2, phospho‐JAK2; p‐STAT3, phospho‐STAT3. G) The key protein molecules in JAK2/STAT3 signaling pathway were analyzed in CSCs and adherent cells of Hep3B cell line by western blot. p‐JAK2, phospho‐JAK2; p‐STAT3, phospho‐STAT3. CSCs were enriched by the tumorsphere assay. Columns, means from three independent experiments; bars, SD. ** *p* < 0.01, * *p* < 0.05.

We next assessed whether JAK2 was the miR‐6887‐3p target. The JAK2‐encoded messenger RNA (mRNA) contained a 3'‐untranslated region (3'UTR) element that was partially complementary to miR‐6887‐3p (Figure S5D, Supporting Information), indicating that miR‐6887‐3p would directly target this site. JAK2 expression in Huh7 cells with or without the transfection of miR‐6887‐3p mimic or inhibitor was detected at both the mRNA and protein level. As shown in Figure 5C, after treatment with the miR‐6887‐3p mimic, JAK2 mRNA was not significantly affected (top panel), but JAK2 protein was significantly decreased (bottom panel). Conversely, JAK2 expression after treatment with the miR‐6887‐3p inhibitor showed the opposite trend (Figure 5D). This finding indicated that miR‐6887‐3p could suppress JAK2 expression at a post‐transcriptional level.

In order to confirm this finding, the 3'‐UTR of JAK2 mRNA containing the wild and mutated type of putative miR‐6887‐3p binding site was inserted into a luciferase reporter vector, generating pMIR‐JAK2‐3'UTR‐WT and pMIR‐JAK2‐3'UTR‐MUT, respectively. The luciferase activity of pMIR‐JAK2‐3'UTR‐WT could be effectively inhibited by miR‐6887‐3p mimic (Figure 5E, top panel) and enhanced by its inhibitor (Figure 5E, bottom panel) whereas no significant change was observed when the binding site was mutated. Western blot then confirmed changes in the expression of key protein molecules in the JAK2/STAT3 signaling pathway following overexpression of circ‐MALAT1 in the Hep3B cell line. Overexpression of circ‐MALAT1 attenuated the inhibitory effect of miR‐6887‐3p on JAK2, thereby increasing the total protein level of JAK2 and enhancing its phosphorylation level (Figure 5F). This further promotes the phosphorylation and activation of STAT3 but not STAT5 (Figure 5E and Figure S5E, Supporting Information). However, the total protein level of STAT3 was not significantly changed (Figure 5F). In addition, the three key protein molecules JAK2, phospho‐JAK2 (p‐JAK2) and phospho‐STAT3 (p‐STAT3) in the JAK2/STAT3 signaling pathway were also confirmed to be upregulated in the CSCs (Figure 5G). Moreover, to further verify the specificity of miR‐6887‐3p in vivo, we also analyzed JAK2 expression in xenograft tumors after treatment with the miR‐6887‐3p mimic or inhibitor. JAK2 mRNA and circ‐MALAT1 were not significantly affected after treatment with the miRNA mimic (**Figure** **6**A, top panel and Figure S5F, Supporting Information), but JAK2 protein was decreased significantly after treatment with the miR‐6887‐3p mimic (Figure 6A, bottom panel). Conversely, JAK2 expression in the tumors treated with the miR‐6887‐3p inhibitor showed the opposite trend (Figure 6B and Figure S5G, Supporting Information). In addition, as MALAT1 attenuated the inhibitory effect of miR‐6887‐3p on JAK2 protein (Figure 6C), p‐JAK2 was enhanced in vivo (Figure 6D). Taken together, up‐regulation of circ‐MALAT1 in CSCs could absorb miR‐6887‐3p and thereby enhance the JAK2/STAT3 pathway activity for the self‐renewal of hepatocellular CSCs.

**Figure 6:**
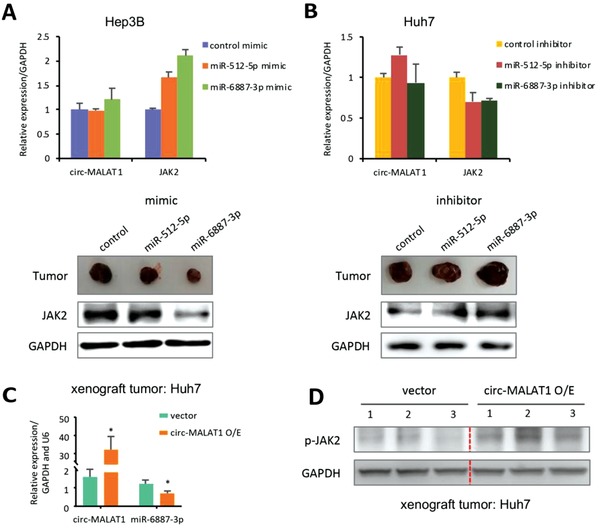
Circ‐MALAT1 absorbs miR‐6887‐3p to upregulate JAK2 in vivo. A) After treatment with miR‐6887‐3p mimic, miR‐512‐5p mimic, or its scrambled version (control mimic), both circ‐MALAT1 and JAK2 mRNA of xenograft tumors were detected by qRT‐PCR (top panel), and JAK2 expression at the protein level was detected by western blot (bottom panel). miR‐512‐5p was used as a control miRNA. GAPDH was used as a control primer. B) After treatment with miR‐6887‐3p inhibitor, miR‐512‐5p inhibitor or its scrambled version (control inhibitor), both circ‐MALAT1 and JAK2 mRNA of xenograft tumors were detected by qRT‐PCR (top panel), and JAK2 expression at the protein level was detected by western blot (bottom panel). miR‐512‐5p was used as a control miRNA. GAPDH was used as a control primer. C) MiR‐6887‐3p expression was analyzed in the xenograft tumors generated by circ‐MALAT1 overexpressed (circ‐MALAT1 O/E) or empty vector (Vector) Huh7 cells. Control primers, GAPDH and U6. D) p‐JAK2 was determined in the xenograft tumors generated by circ‐MALAT1 overexpressed (circ‐MALAT1 O/E) or empty vector (Vector) Huh7 cells. Columns, means from three independent experiments; bars, SD. * *p* < 0.05.

Our results have showed a novel dual‐faceted regulation pathway by a circRNA (**Figure** **7**). Namely, a circRNA can not only enhance the expression of molecules (e.g., JAK2) favoring the CSCs self‐renewal by a more traditional mechanism of sponging a miRNA (e.g., miR‐6887‐3p), but also suppress the expression of molecules (PAX5) disfavoring the CSCs self‐renewal by an unprecedented “brake‐like” mechanism of inhibiting mRNA translation at the ribosome site, termed mRNA braking (Figure 4C). This phenomenon significantly differs from the previous views that the RNA molecules either enhance or suppress the expression of certain molecules, but not in both ways.^32^, ^33^, ^34^ On one hand, circ‐MALAT1 positively promoted liver CSCs self‐renewal by sponging a miRNA (miR‐6887‐3p) to up‐regulate an oncogene (JAK2) and increase phospho‐JAK2, enhancing the JAK2/STAT3 signaling.^34^, ^35^ On the other hand, of importance, we discovered a novel mRNA braking mechanism where the circRNA molecules act as a brake to directly inhibit the PAX5 mRNA translation by binding both the ribosome and PAX5 mRNA. Specifically, circ‐MALAT1 had IRESs but no ORFs, and contained 11 bases complementary with the PAX5 mRNA coding sequence (Figure 4C). Namely, circ‐MALAT1 bound not only the ribosomes (via sequences that acted as IRESs) but also the coding sequence of PAX5 mRNA (via an 11‐base complementary sequence) (Figure 4D–G), forming a specific ternary (ribosome‐circRNA‐mRNA) complex. The ternary complex allowed circ‐MALAT1 to be inlaid between PAX5 coding sequence and ribosome like a brake, directly obstructing the translation of PAX5 mRNA and affecting PAX5‐related cellular functions.

**Figure 7:**
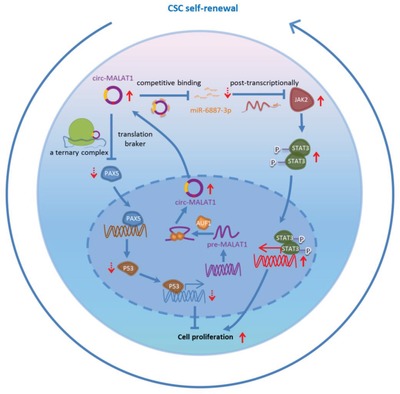
Schematic diagram for the mechanisms of circ‐MALAT1 functioning as both an mRNA translation brake and an miRNA sponge to promote self‐renewal of hepatocellular CSCs.

We believe that the mRNA braking, through the formation of the unprecedented ternary complex, ribosome‐circ‐MALAT1‐PAX5 mRNA, is a novel regulatory mode of circRNAs under the physiological and pathological conditions. In fact, this is also a new mode for noncoding RNAs to negatively regulate the translation of coding genes. It has been extensively reported that miRNAs perform their post‐transcriptional repression mainly in two ways. One is to bind the target 3'UTR via imperfect base pairing to repress translation directly.^36^ The other way is to slice and decay the target mRNA (when base pairing is very perfect and extensive), resulting in reduction of the amount of mRNA and its corresponding protein.^36^, ^37^ In addition, lncRNAs have been revealed to recruit proteins to mRNAs, thereby mediating the decay of these mRNAs.^38^ However, we discovered that circRNAs inhibit mRNA translation in a completely different mechanism (i.e., mRNA braking) than those reported for the miRNAs and lncRNAs. Further in‐depth studies of our discovered mRNA braking mechanism of circRNAs are expected to unlock a new chapter in revealing the role of circRNAs in regulating life activities.

## Experimental Section

*Human Samples*: The tumor tissues for isolation of HCC primary cells were collected from HCC patients at the Second Affiliated Hospital of Navy Medical University (Shanghai, China), which was authorized by the Committee on Ethics of Medicine of Navy Medical University. Written informed consent was obtained from the volunteers.

*Animal Models*: Male BALB/c nude mice without specific pathogens of 4–5 w were purchased from the Charles River Laboratories (Beijing, China) and maintained under a specific pathogen‐free condition. All experiments involving mice were conducted in accordance with the guidelines for animal welfare formulated by the laboratory animal center at Navy Medical University.

*Cell Lines*: HEK293T and human HCC cell lines Hep3B, Huh7, HepG2, and SMMC‐7721 were acquired from the American Tissue Culture Collection (ATCC) and cultured as previously described.^39^

## Conflict of Interest

The authors declare no conflict of interest.

## Supporting information

Supporting Information
