# Supplementary material for: Circ‐MALAT1 Functions as Both an mRNA Translation Brake and a microRNA Sponge to Promote Self‐Renewal of Hepatocellular Cancer Stem Cells
Source: Adv Sci (Weinh). 2019 Dec 21;7(4):1900949. doi: 10.1002/advs.201900949 (PMC7029649; doi:10.1002/advs.201900949)
Supplement: Supplementary file 1 — Supporting Information [file ADVS-7-1900949-s001.pdf]

Copyright WILEY-VCH Verlag GmbH & Co. KGaA, 69469 Weinheim, Germany, 2019.

ADVANCED SCIENCE
Open Access

Supporting Information

for *Adv. Sci.,* DOI: 10.1002/advs.201900949

Circ-MALAT1 Functions as Both an mRNA Translation Brake and a microRNA Sponge to Promote Self-Renewal of Hepatocellular Cancer Stem Cells

Liang Chen, Ruijiao Kong, Cong Wu, Shuo Wang, Zixin Liu, Shupeng Liu, Shuiping Li, Tian Chen, Chuanbin Mao, and Shanrong Liu**

Supplementary Materials

Supplemental Figures

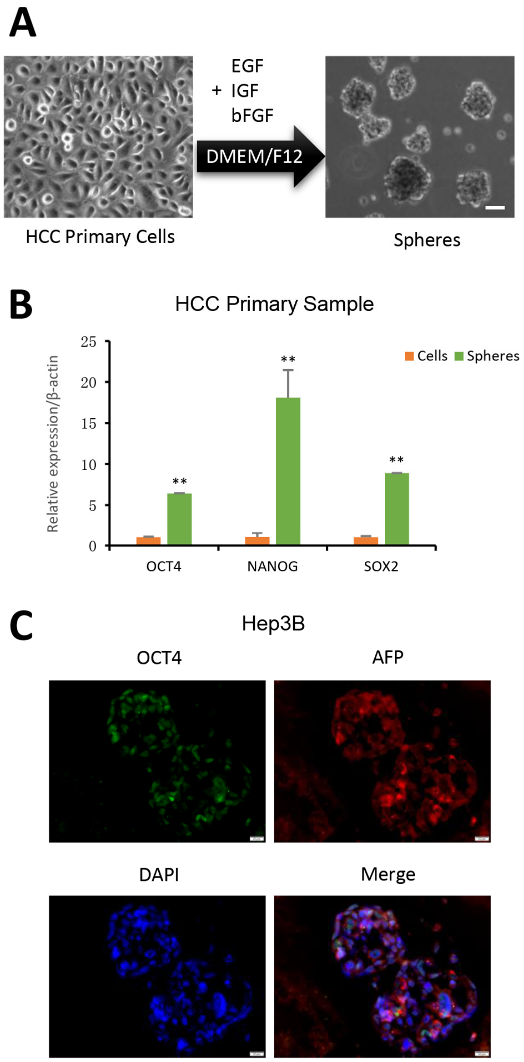

Supplementary Figure 1 (related to Figure 1). Enrichment of CSCs by serum-free medium culture.

(A) Pictures of tumorsphere formation were imaged by a light microscope. Scale bar, 50 µm. (B) The mRNA levels of CSC markers, including OCT4, NANOG and SOX2 were measured by qRT-PCR. Control primers, β-actin. (C) The protein level of CSC marker OCT4 was measured in spheres by immunofluorescence. Scale bar, 20 µm. Columns, mean from three independent experiments; bars, SD. ** $P< 0.01$.

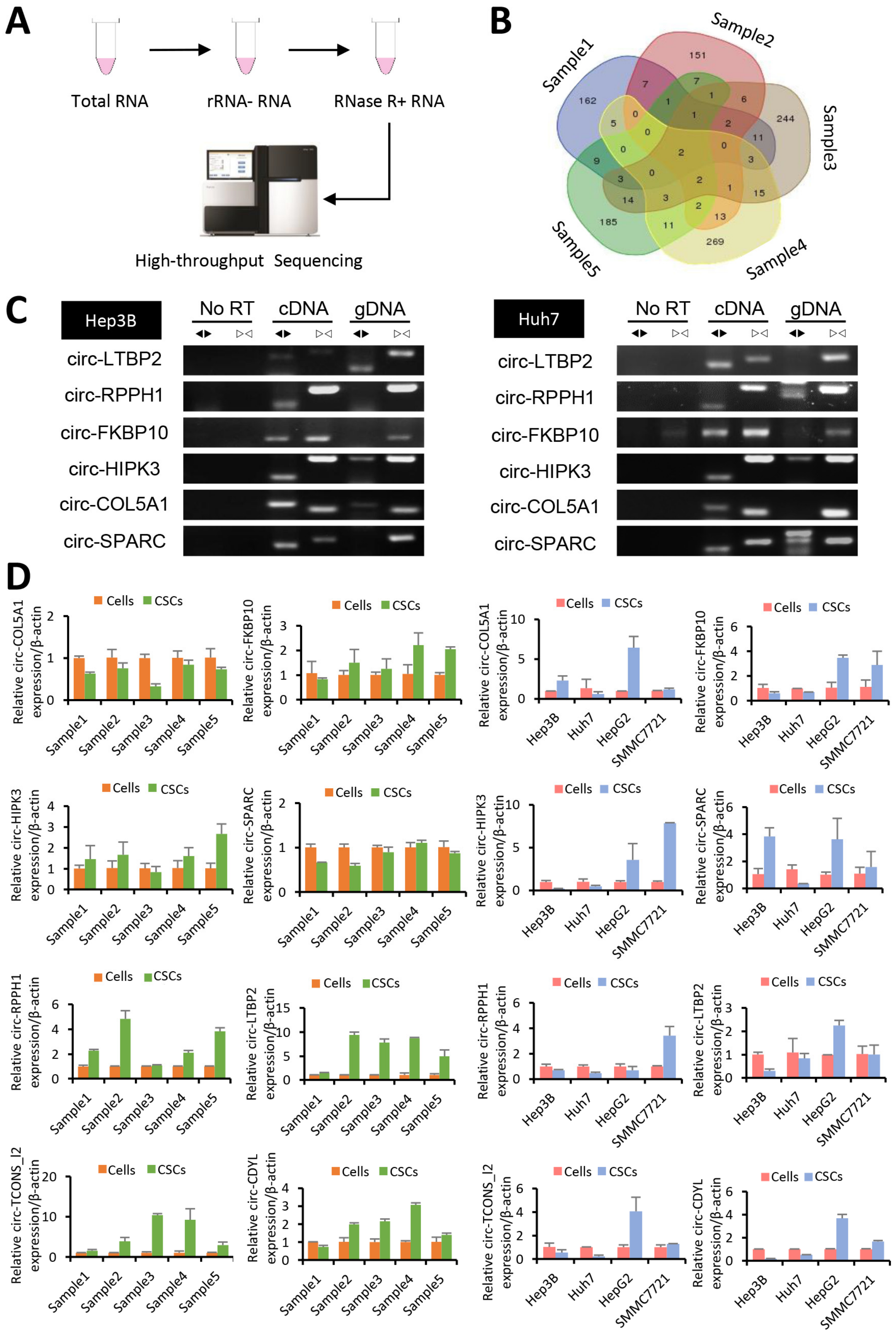

Supplementary Figure 2 (related to Figure 1). CircRNAs are expressed differentially in HCC CSCs.

(A) Total RNA was depleted of rRNA by using RiboMinus method. rRNA-depleted samples (rRNA−) were then treated with the RNase R exonuclease (RNase R+). Sequencing libraries were prepared from each and samples were sequenced and compared. (B) Venn diagram showing that 2 circRNAs were identified in all five pairs of samples and 21 were identified in at least three of the five pairs of samples. (C) Divergent primers amplified 6 candidate circRNAs in cDNA but not genomic DNA (gDNA) and RNA (No RT). (D) Differential expression of candidate circRNAs in CSCs and matched adherent cells of HCC primary cells or HCC cell lines. Control primers, β-actin. CSCs were enriched by tumorsphere assay. Columns, mean from three independent experiments; bars, SD.

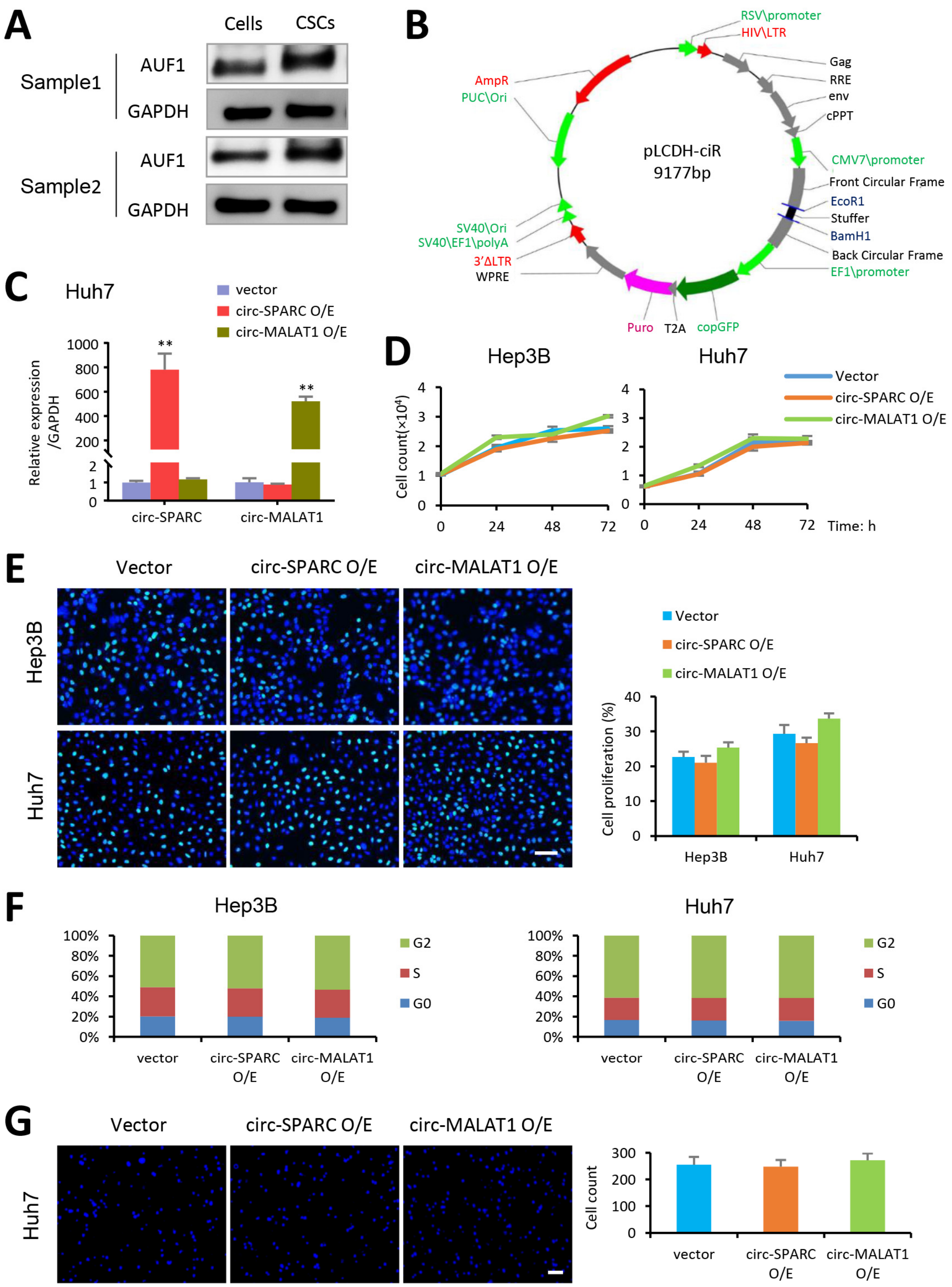

Supplementary Figure 3 (related to Figure 2). Circ-MALAT1 has no significant effect on cell proliferation, cell cycle and migration of HCC cells.

(A) AUF1 was analyzed in CSCs and matched adherent cells of HCC primary cells (Sample1 and Sample2) by western blot. CSCs were enriched by tumorsphere assay. (B) Vector map of construct used for circ-MALAT1 overexpression experiments. (C) qRT-PCR confirmed that

circ-SPARC and circ-MALAT1 were specifically overexpressed when cells were transfected with the circ-SPARC overexpression plasmid (circ-SPARC O/E) and circ-MALAT1 overexpression plasmid (circ-MALAT1 O/E) using divergent primers, respectively. Vector, empty plasmid. Control primers, β-actin. (D) (E) Proliferation of Hep3B and Huh7 cells transfected with circ-MALAT1 overexpression plasmid (circ-MALAT1 O/E), circ-SPARC overexpression plasmid (circ-SPARC O/E), or empty plasmid (Vector) was detected by Cell Counting Kit–8 assay and EdU imaging analysis. Scale bar, 100 μm. (F) There was no significant difference between circ-MALAT1 overexpression (circ-MALAT1 O/E), circ-SPARC overexpression (circ-SPARC O/E), and empty vector control (Vector) on the cell cycle progression of both Hep3B and Huh7 cells. (G) Transwell assays showed no significant effect of circ-MALAT1 on cancer cell invasion. Scale bar, 100 μm. Vector, empty plasmid; circ-SPARC O/E, circ-SPARC overexpression plasmid; circ-MALAT1 O/E, circ-MALAT1 overexpression plasmid. Similar results were obtained in three to six independent experiments. ** $P < 0.01$.

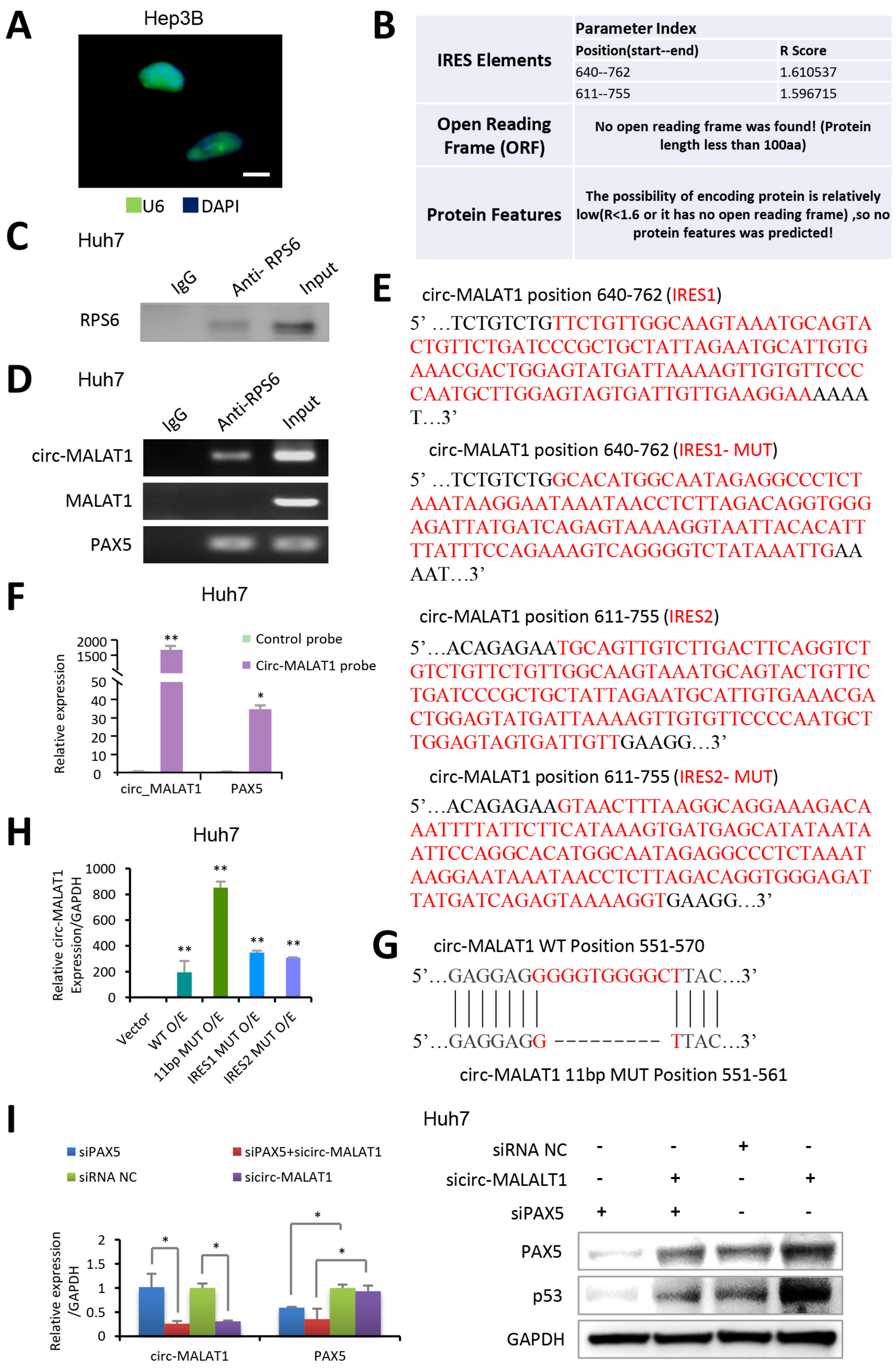

Supplementary Figure 4 (related to Figure 4). Circ-MALAT1 obstructs PAX5 translation by binding to PAX5 coding sequence and ribosomes.

(A) RNA fluorescence *in situ* hybridization for U6. Nuclei were stained with DAPI. Green staining signal against U6 was localized in nucleus. Scale bar, 10 μm. (B) Analysis was performed by the circRNADb and ORFfinder databases to predict the potential IRESs and ORFs. (C) RPS6 antibody was confirmed by western blot. RPS6 protein was observed in the anti-RPS6 RIP (lane 2) and 10% Input (lane 3) but not the IgG RIP (Lane1). (D) RIP was performed using Huh7 cell lysate and either anti-RPS6 or IgG as IP antibody. PCR products of Circ-MALAT1 and PAX5 were observed in the anti-RPS6 RIP (lane RPS6) and 10% Input and substantially less was detected in the IgG RIP (Lane IgG). MALAT1 PCR product was only clearly observed in 10% input. (E) Predicted two IRES elements in circ-MALAT1 were mutated as shown. (F) *In vivo* circ-MALAT1 pull-down using circ-MALAT1 probe 2 was performed in circ-MALAT1 overexpressed Huh7 cells, followed by qRT-PCR to detect circ-MALAT1 (left panel) and PAX5 (right panel). (G) 11 bases complementary to PAX5 coding sequence in circ-MALAT1 were mutated as shown. (H) The level of circ-MALAT1 was detected in Huh7 cells overexpressing circ-MALAT1 wild (WT O/E) or mutant type (11bp MUT O/E, IRES1 MUT O/E and IRES2 MUT O/E) or not (Vector) by qRT-PCR. Control primers, GAPDH. (I) Left panel, RNA levels of circ-MALAT1 and PAX5 were analyzed by qRT-PCR. Right panel, protein levels of PAX5 and p53 were analyzed by western blot. siRNA NC, scramble control siRNA; sicirc-MALAT1, siRNA against circ-MALAT1; siPAX5, siRNA against PAX5. Control primers, GAPDH. Columns, mean from three independent experiments; bars, SD. ** $P< 0.01$, * $P< 0.05$.

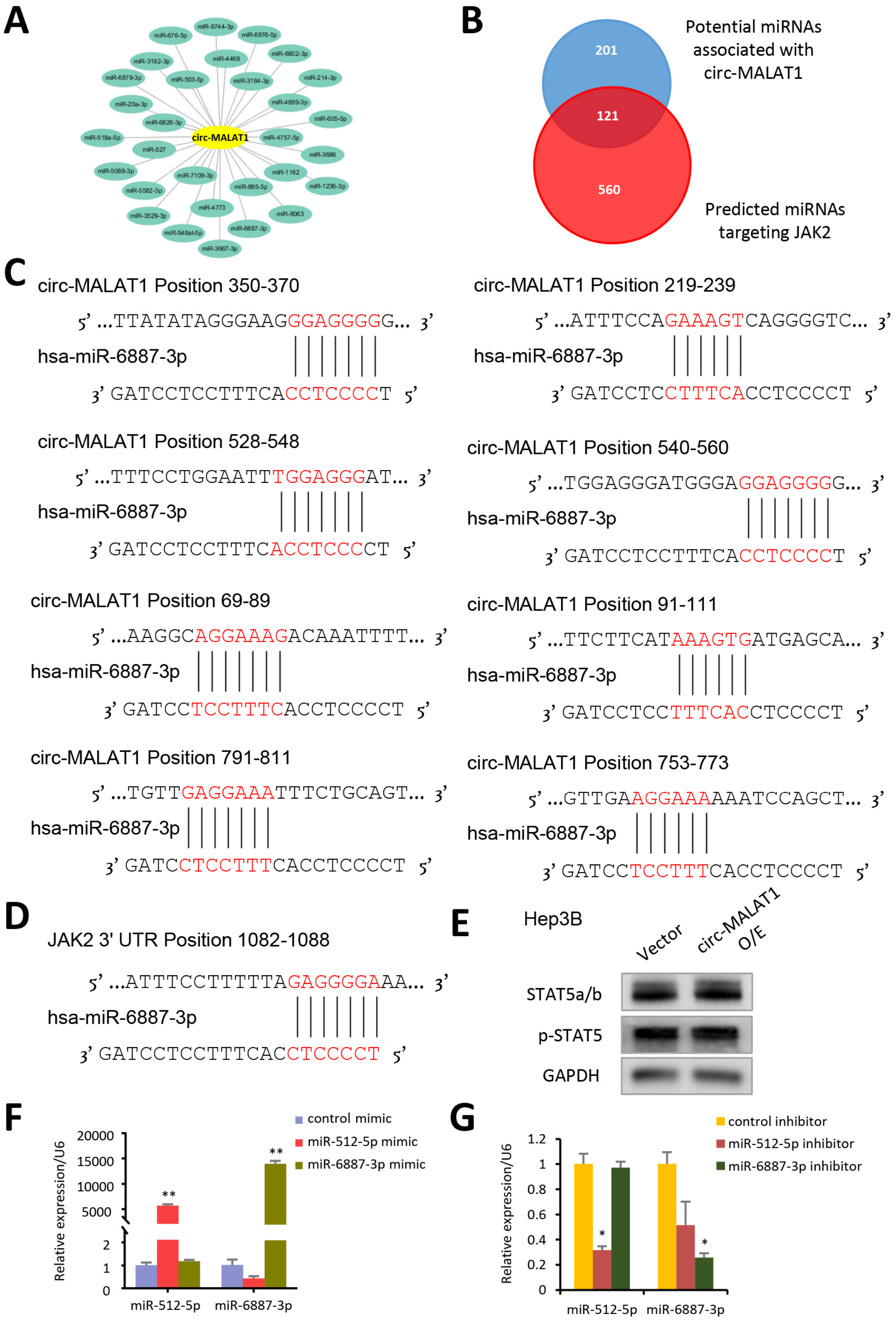

Supplementary Figure 5 (related to Figure 5 and Figure 6). MiR-6887-3p has binding sites with circ-MALAT1 and potentially targets JAK2.

(A) Radial pattern showed a part of 322 miRNA binding sites on circ-MALAT1 predicted by bioinformatics analysis. (B) 121 predicted miRNAs having binding sites with both circ-MALAT1 (blue pie) and potentially targeting JAK2 (red pie) were screened out. (C) Predicted 8 duple combinations between circ-MALAT1 and miR-6887-3p. (D) Predicted duplex combination between the JAK2 3'UTR and miR-6887-3p. (E) STAT5 and phospho-STAT5 (p-STAT5), the downstream molecules of JAK2, were analyzed in cells with circ-MALAT1 overexpressed (circ-MALAT1 O/E) or not (Vector) by western blot. (F) After treatment with miR-6887-3p mimic, miR-512-5p mimic or its scrambled version (control mimic), miRNAs of xenograft tumors were detected by qRT-PCR. miR-512-5p was used as a control miRNA. U6 was used as a control primer. (G) After treatment with miR-6887-3p inhibitor, miR-512-5p inhibitor or its scrambled version (control inhibitor), miRNAs of xenograft tumors were detected by qRT-PCR. miR-512-5p was used as a control miRNA. U6 was used as a control primer. Columns, mean from three independent experiments; bars, SD. ** $P < 0.01$, * $P < 0.05$.

Supplementary Table 1. RNA-binding protein sites matching flanking regions of circ-MALAT1

RNA-binding Protein	#Tags
AGO2	44
FUS	40
AUF1	27
DGCR8	17
HNRNPC	15
IGF2BP2	13
U2AF65	12
HuR	10
PTB	9
ZC3H7B	9
IGF2BP1	8
LIN28A	8
TDP43	7
TIA1	7
IGF2BP3	6
EWSR1	5
LIN28B	5
TIAL1	4
C22ORF28	3
EIF4A3	3
FMBP	3
SFRS1	3
TAF15	3
TNRC6	3
MOV10	2
ALKBH5	1
C17ORF85	1
PUM2	1
QKI	1

Supplementary Table 2. 1358 signaling explorer antibodies of antibody-array

Antibody Name	Reactivity	Swiss Prot	Clonality
14-3-3 beta	H,M,R	P31946	Polyclonal
14-3-3 epsilon	H,M,R	P62258	Polyclonal
14-3-3 eta	H,M,R	Q04917	Polyclonal
14-3-3 gamma	H,M,R	P61981	Polyclonal
14-3-3 theta	H,M,R	P27438	Polyclonal
14-3-3 zeta	H,M,R	P63104	Polyclonal
4E-BP1	H	Q13541	Monoclonal
5-HT-1A	H,M,R	P08908	Polyclonal
5-HT-1F	H,M,R	P30939	Polyclonal
5-HT-2C	H,M,R	P28335	Polyclonal
5-HT-3A	H	P46098	Polyclonal
5-HT-4	H,M,R	Q13639	Polyclonal
5-HT-5A	H	P47898	Polyclonal
60S Ribosomal Protein L10	H,M,R	P27635	Polyclonal
6-Phosphofructo-2-Kinase	H	O60825	Polyclonal
A1BG	H	P04217	Monoclonal
A26C2/3	H	Q6S5H5	Polyclonal
AARSD1	H,M,R	Q9BTE6	Polyclonal
AASDHPPT	H,M,R	Q9NRN7	Polyclonal
AATF	H,M,R	Q9NY61	Polyclonal
ABCA8	H,M	O94911	Polyclonal
ABCB7	H	O75027	Polyclonal
ABCD1	H	P33897	Polyclonal
ABHD11	H	Q8NFV4	Polyclonal
ABHD12	H,M,R	Q8N2K0	Polyclonal
ABHD12B	H	Q7Z5M8	Polyclonal
ABHD14A	H,M,R	Q9BUJ0	Polyclonal
ABHD14B	H,M,R	Q96IU4	Polyclonal
ABHD4	H,M	Q8TB40	Polyclonal
ABL1	H,M,R	P00519	Polyclonal
ACAD10	H	Q6JQN1	Polyclonal
ACBD6	H,M,R	Q9BR61	Polyclonal
ACOT2	H	P49753	Polyclonal
ACOT4	H	Q8N9L9	Polyclonal
ACSL6	H,M,R	Q9UKU0	Polyclonal

Actin-alpha-1	H,M,R	P68133	Polyclonal
Actin-gamma2	H,M,R	P63267	Polyclonal
Actin-pan	H,M,R	P60709	Polyclonal
ACTL6A	H,M,R	O96019	Polyclonal
ACTN 1/2/3/4	H,M,R	P12814	Polyclonal
ACTN alpha-2/3	H,M,R	P35609/Q08043	Polyclonal
ACTR-1C	H,M,R	Q8NER5	Polyclonal
ACTR3	H,M,R	P61158	Polyclonal
ACVL1	H,M,R	P37023	Polyclonal
ADA2L	H,M,R	O75478	Polyclonal
ADAM 17 (Cleaved-Arg215)	H	P78536	Polyclonal
ADAR1	H,M,R	P55265	Polyclonal
ADCK1	H,M	Q86TW2	Polyclonal
ADCK2	H	Q7Z695	Polyclonal
ADCK3	H,M,R	Q8NI60	Polyclonal
ADCK5	H	Q3MIX3	Polyclonal
ADCY4	H,M,R	Q8NFM4	Polyclonal
ADCY5/6	H,M,R	O43306	Polyclonal
ADCY7	H,M	P51828	Polyclonal
ADCY8	H,M,R	P40145	Polyclonal
ADD2	H,M,R	P35612	Polyclonal
ADD3	H,M,R	Q9UEY8	Polyclonal
ADH7	H	P40394	Polyclonal
ADK	H,M,R	P55263	Polyclonal
ADNP	H,M	Q9H2P0	Polyclonal
ADPGK	H,M,R	Q9BRR6	Polyclonal
ADRB1	H,M,R	P08588	Polyclonal
Adrenergic Receptor alpha-2A	H,M,R	P08913	Polyclonal
Adrenergic Receptor alpha-2B	H	P18089	Polyclonal
Adrenergic Receptor alpha-2C	H,M,R	P18825	Polyclonal
AFP	H	P02771	Monoclonal
Aggrecan (Cleaved-Asp369)	H,R	P16112	Polyclonal
AGR3	H,M	Q8TD06	Polyclonal
AIFM2	H,M	Q9BRQ8	Polyclonal
AIG1	H	Q9NVV5	Polyclonal
AIRE	H,M	O43918	Polyclonal
AKR1B1	H,R	P15121	Polyclonal

AKR1CL1	H	Q5T2L2	Polyclonal
AKR1CL2	H	Q96JD6	Polyclonal
Akt	H,M,R	P31749	Polyclonal
AKT2	H,R	P31751	Monoclonal
Akt3	H	Q9Y243	Monoclonal
ALCAM	H	Q13740	Monoclonal
ALDH1A2	H,M,R	O94788	Polyclonal
ALDH1B1	H	P30837	Polyclonal
ALDH3B1	H	P43353	Polyclonal
ALDOB	H,M,R	P05062	Polyclonal
ALDOC	H,M,R	P09972	Polyclonal
alpha hCG	H	P01215	Monoclonal
AMACR	H,M	Q9UHK6	Monoclonal
AMPD1	H,M,R	P23109	Polyclonal
Amylin	H	P10997	Polyclonal
Androgen receptor	H	P10275	Monoclonal
Angiopoietin-1	H,M	Q15389	Polyclonal
Angiopoietin-2	H,M	O15123	Polyclonal
Annexin A6	H	P08133	Polyclonal
AOS1	H,M,R	Q9UBE0	Polyclonal
AP-2	H,M,R	P05549	Polyclonal
AP2C	H,M,R	Q92754	Polyclonal
APAF-1-ALT	H	O14727	Polyclonal
APC	H,M,R	P25054	Polyclonal
APC6	H,M,R	Q13042	Polyclonal
APLP2	H,M,R	Q06481	Polyclonal
APOF	H	Q13790	Polyclonal
APOL1	H	O14791	Polyclonal
APOL2	H	Q9BQE5	Polyclonal
ARC	H,M	O60936	Polyclonal
ARF4	H,M,R	P18085	Polyclonal
ARFGEF2	H,M,R	Q9Y6D5	Polyclonal
ARFIP1	H,M,R	P53367	Polyclonal
ARHGEF10	H	O15013	Polyclonal
ARHGEF12	H,M,R	Q9NZN5	Polyclonal
ARHGEF2	H	Q92974	Polyclonal
ARHGEF3	H,M	Q9NR81	Polyclonal

ARHGEF5	H,M,R	Q12774	Polyclonal
ARHGEF9	H,M,R	O43307	Polyclonal
ARPP21	H,M	Q9UBL0	Polyclonal
ARSA	H,M,R	P15289	Polyclonal
ARSD	H	P51689	Polyclonal
ARSI	H	Q5FYB1	Polyclonal
ARSK	H,M,R	Q6UWY0	Polyclonal
ASC	H,M,R	Q9ULZ3	Polyclonal
ATBP3	H	Q7Z7A3	Polyclonal
ATF1	H,M,R	P18846	Polyclonal
ATF3	H,M,R	P18847	Polyclonal
ATF5	H,M,R	Q9Y2D1	Polyclonal
ATF6B	H	Q99941	Polyclonal
ATF7	H,M	P17544	Polyclonal
ATG4B	H,M	Q9Y4P1	Polyclonal
ATP2C1	H	P98194	Monoclonal
ATP5A1	H,M,R	P25705	Polyclonal
ATP5D	H,M,R	P30049	Polyclonal
ATP5G2	H	Q06055	Polyclonal
ATP5G3	H,R	P48201	Polyclonal
ATP5H	H,M,R	O75947	Polyclonal
ATP5S	H,M	Q99766	Polyclonal
ATP6V1B1	H,M	P15313	Polyclonal
ATP6V1H	H,M	Q9UI12	Polyclonal
ATP7B	H,M,R	P35670	Polyclonal
ATPAF2	H,M	Q8N5M1	Polyclonal
ATPG	H,M,R	P36542	Polyclonal
ATRX	H,M	P46100	Polyclonal
AURKB	H	Q96GD4	Monoclonal
AVEN	H	Q9NQS1	Polyclonal
AXL	H	P30530	Monoclonal
BACH1	H	O14867	Polyclonal
BAD	H,M,R	Q92934	Polyclonal
BAD (Cleaved-Asp71)	M,R	Q61337	Polyclonal
BAGE2	H	Q86Y30	Polyclonal
BAGE3	H	Q86Y29	Polyclonal
BAGE4	H	Q86Y28	Polyclonal

Bak	H,M	Q16611	Polyclonal
BARD1	H	Q99728	Polyclonal
Bax	H,M,R	Q07812	Polyclonal
BCA3	H	Q9NQ31	Polyclonal
BCL-10	H,M	O95999	Monoclonal
BCLW	H,M,R	Q92843	Polyclonal
beta hCG	H	P01233	Monoclonal
beta-2-Microglobulin	H	P61769	Monoclonal
Beta-Actin	H,M,R	P60709	Monoclonal
BIM	H,M	O43521	Polyclonal
BLCAP	H,M,R	P62952	Polyclonal
BLK	H	P51451	Monoclonal
BMP8A	H	Q7Z5Y6	Polyclonal
BMX (ETK)	H	P51813	Monoclonal
B-RAF	H,M	P15056	Polyclonal
BRCA2	H,R	P51587	Polyclonal
BRI3B	H,M	Q8WY22	Polyclonal
BRMS1	H,M,R	Q9HCU9	Polyclonal
BRSK1	H,M	Q8TDC3	Polyclonal
BST2	H	Q10589	Polyclonal
BUB1	H,M	O43683	Polyclonal
BUB1B	H,M	O60566	Polyclonal
BUB3	H,M,R	O43684	Polyclonal
C140	H,M,R	Q9NVX2	Polyclonal
C1R (light chain,Cleaved-Ile464)	H	P00736	Polyclonal
C1S	H	P09871	Polyclonal
C1S (heavy chain,Cleaved-Arg437)	H	P09871	Polyclonal
C3AR1	H	Q16581	Polyclonal
C56D2	H	O14569	Polyclonal
C5orf13	H,M,R	Q16612	Polyclonal
C9	H	P02748	Polyclonal
C9orf89	H,M	Q96LW7	Polyclonal
CA 15-3	H	P15941	Monoclonal
CA125	H	Q8WXI7	Monoclonal
CA13	H,M	Q8N1Q1	Polyclonal
CA14	H,M,R	Q9ULX7	Polyclonal
CA181	H,M	Q9NWK9	Polyclonal

CA19-9	H	P78552	Monoclonal
CA5B	H,M,R	Q9Y2D0	Polyclonal
CA6	H	P23280	Polyclonal
Cadherin-pan	H,M,R	P12830	Polyclonal
CAGE1	H	Q8TC20	Polyclonal
Calcyclin (S100A6)	H	P06703	Monoclonal
Calnexin	H	P27824	Monoclonal
Calreticulin	H,M	P27797	Monoclonal
CaMK1-beta	H,M,R	Q6P2M8	Polyclonal
CaMK2beta/gamma	H,M,R	Q13554/Q13555	Polyclonal
CAMK5	H,M,R	Q8NCB2	Polyclonal
cAMP	H,M,R	N/A	Monoclonal
CARD6	H	Q9BX69	Polyclonal
CARKL	H,M,R	Q9UHJ6	Polyclonal
CASP1 (p20,Cleaved-Asn120)	H	P29466	Polyclonal
CASP2 (p18,Cleaved-Gly170)	H	P42575	Polyclonal
CASP2 (p18,Cleaved-Thr325)	H,M,R	P42575	Polyclonal
CASP3 (p17,Cleaved-Asp175)	H,M,R	P42574	Polyclonal
CASP4 (p20,Cleaved-Gln81)	H	P49662	Polyclonal
CASP5 (p10,Cleaved-Ser331)	H	P51878	Polyclonal
CASP5 (p20,Cleaved-Asp121)	H	P51878	Polyclonal
CASP8 (Cleaved-Asp384)	H	Q14790	Polyclonal
Caspase 10	H	Q92851	Polyclonal
Caspase 3 (Cleaved-Asp175)	H,M,R	P42574	Polyclonal
Caspase 6 (Cleaved-Asp162)	H,R	P55212	Polyclonal
Caspase 7 (Cleaved-Asp198)	H,M	P55210	Polyclonal
Caspase 9 (Cleaved-Asp315)	H	P55211	Polyclonal
Caspase 9 (Cleaved-Asp330)	H	P55211	Polyclonal
Caspase 9 (Cleaved-Asp353)	M,R	Q9R0S9	Polyclonal
CATD (heavy chain,Cleaved-Leu169)	H	P07339	Polyclonal
CATD (light chain,Cleaved-Gly65)	H	P07339	Polyclonal
Catenin-alpha1	H,M,R	P35221	Polyclonal
Catenin-beta 1	H,M,R	P35222	Polyclonal
Catenin-gamma	H,M,R	P14923	Polyclonal
CATG (Cleaved-Ile21)	H,M,R	P08311	Polyclonal
CATL1 (heavy chain,Cleaved-Thr288)	H	P07711	Polyclonal
CATL2 (Cleaved-Leu114)	H	O60911	Polyclonal

CATZ (Cleaved-Leu62)	H	Q9UBR2	Polyclonal
Caveolin-1	H,M,R	Q03135	Polyclonal
CBLN1	H,M,R	P23435	Polyclonal
CBLN2	H,M,R	Q8IUK8	Polyclonal
CBLN3	H,M	Q6UW01	Polyclonal
CBLN4	H,M	Q9NTU7	Polyclonal
CBP (Acetyl-Lys1535)	H,M,R	Q92793	Polyclonal
CBR3	H,M,R	O75828	Polyclonal
CCT6A	H,M,R	P40227	Polyclonal
CD10	H	P08473	Monoclonal
CD14	H	P08571	Monoclonal
CD153	H,M	P32971	Polyclonal
CD154 (sCD40-Ligand)	H	P29965	Polyclonal
CD18 (ITGB2)	H,M	P05107	Monoclonal
CD19	H	P15391	Monoclonal
CD2 Tail-binding	H	O95400	Polyclonal
CD247 (CD3Z)	H	P20963	Monoclonal
CD253	H	P50591	Polyclonal
CD3	H	P04234/P20963/<br>P09693/P07766	Monoclonal
CD302	H,M,R	Q8IX05	Polyclonal
CD31	H	P16284	Monoclonal
CD33	H	P20138	Monoclonal
CD34	H	P28906	Monoclonal
CD37	H	P11049	Monoclonal
CD38	H	P28907	Monoclonal
CD3E	H	P07766	Monoclonal
CD3EAP	H	O15446	Polyclonal
CD40	H,M	P25942	Polyclonal
CD44	H,M	P16070	Monoclonal
CD45	H	P08575	Monoclonal
CD55	H	P08174	Polyclonal
CD69	H	Q07108	Monoclonal
CD8	H	P01732/P10966	Monoclonal
CD80	H	P33681	Monoclonal
CD97beta (Cleaved-Ser531)	H	P48960	Polyclonal
CDC2	H,M,R	P06493	Polyclonal

CDC25C	H	P30307	Monoclonal
CDC40	H,M,R	O60508	Polyclonal
CDC6	H	Q99741	Polyclonal
CDC7	H	O00311	Polyclonal
CDCA2	H	Q69YH5	Polyclonal
CDCA3	H,M,R	Q99618	Polyclonal
CDCA4	H,M	Q9BXL8	Polyclonal
CDCA7	H,M,R	Q9BWT1	Polyclonal
CDCP1	H,M	Q9H5V8	Polyclonal
CDH10	H,M	Q9Y6N8	Polyclonal
CDH11	H,M	P55287	Polyclonal
CDH18	H,M	Q13634	Polyclonal
CDH2	H,M,R	P19022	Polyclonal
CDH20	H,M,R	Q9HBT6	Polyclonal
CDH24	H,M	Q86UP0	Polyclonal
CDH3	H	P22223	Polyclonal
CDH4	H,M	P55283	Polyclonal
CDH8	H,M,R	P55286	Polyclonal
CDH9	H,M	Q9ULB4	Polyclonal
CDK2	H,M,R	P24941	Polyclonal
CDK5R1	H,M,R	Q15078	Polyclonal
CDK5R2	H	Q13319	Polyclonal
CDK7	H,M	P50613	Polyclonal
CDK8	H,M	P49336	Polyclonal
CDKA1	H,M	O14519	Polyclonal
CDKA2	H,M	O75956	Polyclonal
CDKL1	H,R	Q00532	Polyclonal
CDKL2	H	Q92772	Polyclonal
CDKL3	H	Q8IVW4	Polyclonal
CDKN1B	H	Q6I9V6	Monoclonal
CDYL2	H	Q8N8U2	Polyclonal
CEA	H	P11465	Monoclonal
CEBPE	H,M,R	Q15744	Polyclonal
CER1	H	O95813	Monoclonal
CFAB Bb (Cleaved-Lys260)	H,M	P00751	Polyclonal
CHD4	H,M	Q14839	Polyclonal
CHK1	H,M	O14757	Monoclonal

CHK2	H	O96017	Monoclonal
CHKB	H	Q9Y259	Polyclonal
CHML	H	P26374	Polyclonal
CHP2	H,M,R	O43745	Polyclonal
CHST10	H,M,R	O43529	Polyclonal
CHST2	H	Q9Y4C5	Polyclonal
CHST6	H	Q9GZX3	Polyclonal
CHST8	H,M,R	Q9H2A9	Polyclonal
CIB1	H	Q99828	Monoclonal
CIB2	H,M,R	O75838	Polyclonal
CIB3	H,M	Q96Q77	Polyclonal
CIDEB	H,M	Q9UHD4	Polyclonal
CKI-alpha	H,M,R	P48729	Polyclonal
CKI-alpha1/L	H,M,R	Q8N752	Polyclonal
CKI-gamma1	H,M,R	Q9HCP0	Polyclonal
CKI-gamma2	H,M,R	P78368	Polyclonal
C-Kit	H	P10721	Monoclonal
CKLF2	H	Q8TAZ6	Polyclonal
CLASP1	H,M	Q7Z460	Polyclonal
Claudin 1	H,M,R	O95832	Polyclonal
Claudin 10	H,M	P78369	Polyclonal
Claudin 11	H,M,R	O75508	Polyclonal
Claudin 2	H,M	P57739	Polyclonal
Claudin 3	H,M,R	O15551	Polyclonal
Claudin 4	H,M,R	O14493	Polyclonal
Claudin 5	H,M,R	O00501	Polyclonal
Claudin 7	H,M,R	O95471	Polyclonal
CLCC1	H	Q96S66	Polyclonal
CLDN19	H,R	Q8N6F1	Polyclonal
CLDN6	H	P56747	Polyclonal
CLIC3	H	O95833	Polyclonal
CLIC4	H,M,R	Q9Y696	Polyclonal
CLIP1	H,M	P30622	Polyclonal
CLK1	H,M	P49759	Polyclonal
CLK2	H,M,R	P49760	Polyclonal
CLN6	H	Q9NWW5	Polyclonal
CMC1	H	O75746	Polyclonal

CMKLR1	H,M,R	Q99788	Polyclonal
CNGA2	H,M,R	Q16280	Polyclonal
CNKR2	H,M,R	Q8WXI2	Polyclonal
CNN2	H,M	Q99439	Polyclonal
CNTD2	H	Q9H8S5	Polyclonal
CNTN4	H,M,R	Q8IWV2	Polyclonal
CNTROB	H	Q8N137	Polyclonal
Cofilin	H,M,R	Q9Y281	Polyclonal
Collagen alpha1 XVIII	H,M	P39060	Polyclonal
Collagen I	H,M,R	P08123	Polyclonal
Collagen I alpha2	H	P08123	Polyclonal
Collagen I alpha2 (Cleaved-Gly1102)	H	P08123	Polyclonal
Collagen II	H,M,R	P02458	Polyclonal
Collagen III	H,M,R	P02461	Polyclonal
Collagen III alpha1 (Cleaved-Gly1221)	H	P02461	Polyclonal
Collagen IV	H,M	P02462	Polyclonal
Collagen IV alpha2	H,M	P08572	Polyclonal
Collagen IV alpha3	H	Q01955	Polyclonal
Collagen IV alpha3 (Cleaved-Leu1425)	H	Q01955	Polyclonal
Collagen IV alpha3 (Cleaved-Pro1426)	H	Q01955	Polyclonal
Collagen IV alpha4	H	P53420	Polyclonal
Collagen IV alpha5	H,M	P29400	Polyclonal
Collagen IV alpha6	H	Q14031	Polyclonal
Collagen IX alpha3	H	Q14050	Polyclonal
Collagen V alpha1	H	P20908	Polyclonal
Collagen V alpha2	H	P05997	Polyclonal
Collagen VI alpha3	H,M	P12111	Polyclonal
Collagen XI alpha1	H,M	P12107	Polyclonal
Collagen XII alpha1	H	Q99715	Polyclonal
Collagen XIV alpha1	H,M	Q05707	Polyclonal
Collagen XIX alpha1	H	Q14993	Polyclonal
Collagen XVIII alpha1	H,M	P39060	Polyclonal
Collagen XX alpha1	H,M	Q9P218	Polyclonal
Collagen XXIII alpha1	H,M,R	Q86Y22	Polyclonal
Collagen XXV alpha1	H,M	Q9BXS0	Polyclonal
Connexin 43	H,M,R	P17302	Polyclonal
COPZ1	H,M	P61923	Polyclonal

COT2	H,M,R	P24468	Polyclonal
COX1	H	P23219	Polyclonal
COX11	H,M	Q9Y6N1	Polyclonal
COX15	H,M,R	Q7KZN9	Polyclonal
COX17	H,M,R	Q14061	Polyclonal
COX19	H,M	Q49B96	Polyclonal
COX2	H	P35354	Polyclonal
COX41	H	P13073	Polyclonal
COX7S/A2	H,M,R	O60397	Polyclonal
CPB2	H	Q96IY4	Polyclonal
CPM	H	P14384	Polyclonal
CPN1	H	P15169	Polyclonal
CPNE8	H,M,R	Q86YQ8	Polyclonal
CREB-BP	H,M	Q92793	Polyclonal
CREBZF	H,M	Q9NS37	Polyclonal
CREM	H,M,R	Q03060	Polyclonal
CRP	H	P02741	Monoclonal
CRYAB	H	P02511	Monoclonal
CSE1L	H	P55060	Polyclonal
CSF-1 (MCSF)	H	P09603	Monoclonal
CSF2 (GM-CSF)	H	P04141	Monoclonal
CSK	H,M,R	P41240	Polyclonal
CST1	H	P01037	Polyclonal
CST2	H	P09228	Polyclonal
CST9L	H	Q9H4G1	Polyclonal
CSTF2T	H,M,R	Q9H0L4	Polyclonal
CSTL1	H	Q9H114	Polyclonal
cTnI (TNNI3)	H	P19429	Monoclonal
Cullin 1	H,M	Q13616	Polyclonal
Cullin 2	H,M	Q13617	Polyclonal
Cullin 3	H,M	Q13618	Polyclonal
CXADR	H,M,R	P78310	Polyclonal
CYB5R1	H,M,R	Q9UHQ9	Polyclonal
CYB5R3	H	P00387	Polyclonal
Cyclin A	H,M,R	P78396	Polyclonal
Cyclin A1	H,M,R	P78396	Polyclonal
Cyclin E1	H,M,R	P24864	Polyclonal

Cyclin F	H	P41002	Polyclonal
Cyclin G	H,M,R	P51959	Polyclonal
Cyclin L1	H,M,R	Q9UK58	Polyclonal
Cyclosome 1	H,M	Q9H1A4	Polyclonal
CYTL1	H	Q9NRR1	Polyclonal
Cytochrome b561 D1	H	Q8N8Q1	Polyclonal
Cytochrome c	H,M,R	P99999	Polyclonal
Cytochrome c-type Heme Lyase	H,M	P53701	Polyclonal
Cytochrome P450 17A1	H	P05093	Polyclonal
Cytochrome P450 19A1	H,M,R	P11511	Polyclonal
Cytochrome P450 1A1/2	H,M,R	P04798	Polyclonal
Cytochrome P450 1A2	H,M,R	P05177	Polyclonal
Cytochrome P450 24A1	H	Q07973	Polyclonal
Cytochrome P450 26A1	H,M,R	O43174	Polyclonal
Cytochrome P450 26C1	H	Q6V0L0	Polyclonal
Cytochrome P450 27A1	H	Q02318	Polyclonal
Cytochrome P450 2A13	H	Q16696	Polyclonal
Cytochrome P450 2A6	H	P11509	Polyclonal
Cytochrome P450 2B6	H	P20813	Polyclonal
Cytochrome P450 2C19	H	P33261	Polyclonal
Cytochrome P450 2C8	H	P10632	Polyclonal
Cytochrome P450 2C8/9/18/19	H	P33260	Polyclonal
Cytochrome P450 2D6	H	Q2XND8/P10635	Polyclonal
Cytochrome P450 2E1	H,M,R	P05181	Polyclonal
Cytochrome P450 2R1	H,M	Q6VVX0	Polyclonal
Cytochrome P450 2S1	H,M	Q96SQ9	Polyclonal
Cytochrome P450 2U1	H,M,R	Q7Z449	Polyclonal
Cytochrome P450 2W1	H	Q8TAV3	Polyclonal
Cytochrome P450 39A1	H,M	Q9NYL5	Polyclonal
Cytochrome P450 3A4/5	H	P08684	Polyclonal
Cytochrome P450 3A43	H	Q9HB55	Polyclonal
Cytochrome P450 3A7	H	A4D288	Polyclonal
Cytochrome P450 4F2	H	P78329	Polyclonal
Cytochrome P450 4X1	H	Q8N118	Polyclonal
Cytochrome P450 4Z1	H	Q86W10	Polyclonal
Cytochrome P450 7B1	H	O75881	Polyclonal
DAK	H,M,R	Q3LXA3	Polyclonal

DAXX	H	Q9UER7	Monoclonal
DCC	H,M,R	P43146	Polyclonal
DCT	H,M	P40126	Polyclonal
DDR1	H	Q08345	Monoclonal
DDR2	H	Q16832	Monoclonal
DDX4	H	Q9NQI0	Monoclonal
Desmin	H,M,R	P17661	Polyclonal
DFF45 (Cleaved-Asp224)	H	O00273	Polyclonal
DFFA	H,M,R	O00273	Polyclonal
DGKD	H	Q16760	Polyclonal
DGKH	H,M	Q86XP1	Polyclonal
DGKK	H	Q5KSL6	Polyclonal
Dipeptidyl-peptidase 1 (heavy chain,Cleaved-Arg394)	H	P53634	Polyclonal
DJ-1	H,M	Q99497	Polyclonal
DLEC1	H	Q9Y238	Polyclonal
DMGDH	H	Q9UI17	Polyclonal
DNA Polymerase alpha	H	P09884	Polyclonal
DNA Polymerase beta	H,M,R	P06746	Polyclonal
DNA Polymerase lambda	H,M,R	Q9UGP5	Polyclonal
DNA Polymerase theta	H	O75417	Polyclonal
DNA Polymerase zeta	H,M	O60673	Polyclonal
DNAJB11	H,M,R	Q9UBS4	Polyclonal
DNAJB4	H,M,R	Q9UDY4	Polyclonal
DNAL1	H,M,R	Q4LDG9	Polyclonal
DNAL4	H,M	O96015	Polyclonal
DNA-PK	H,M	P78527	Polyclonal
DOK3	H,M	Q7L591	Polyclonal
DOK4	H,M	Q8TEW6	Polyclonal
DOK6	H,M	Q6PKX4	Polyclonal
DOK7	H,M	Q18PE1	Polyclonal
DP-1	H	Q14186	Polyclonal
Dynamin-1	H	Q05193	Monoclonal
Dynamin-2	H	P50570	Monoclonal
Dysferlin	H,M	O75923	Polyclonal
Dyskerin	H,M,R	O60832	Polyclonal
E2F2	H,M	Q14209	Polyclonal

E2F4	H,M,R	Q16254	Polyclonal
E2F6	H,M,R	O75461	Polyclonal
E-cadherin	H	P12830	Polyclonal
EDD	H,M	O95071	Polyclonal
EEF1G	H,M,R	P26641	Polyclonal
EFEMP1	H,M,R	Q12805	Polyclonal
EFNA1	H,M,R	P20827	Polyclonal
EFNA2	H,M	O43921	Polyclonal
EFNA2 (Cleaved-Asn188)	H,M	O43921	Polyclonal
EFNA3	H,M,R	P52797	Polyclonal
EFNA4	H,M	P52798	Polyclonal
EFNA5	H,M,R	P52803	Polyclonal
EFNB3	H,M,R	Q15768	Polyclonal
EGF	H	P01133	Monoclonal
EGFR	H	P00533	Monoclonal
EGR1	H	P18146	Monoclonal
EIF4G2	H,M	P78344	Polyclonal
EKI2	H,M	Q9NVF9	Polyclonal
Elk1	H,M,R	P19419	Polyclonal
ELOVL1	H,M	Q9BW60	Polyclonal
ELOVL3	H	Q9HB03	Polyclonal
ELOVL4	H,M	Q9GZR5	Polyclonal
ELOVL5	H	Q9NYP7	Polyclonal
EMR1	H	Q14246	Polyclonal
EMR2	H	Q9UHX3	Polyclonal
EMR3	H	Q9BY15	Polyclonal
ENAH	H,M,R	Q8N8S7	Polyclonal
ENDOGL1	H	Q9Y2C4	Polyclonal
eNOS	H	P29474	Monoclonal
EPCAM	H	P16422	Monoclonal
EPHA1	H	P21709	Polyclonal
EPHA6	H,M,R	Q9UF33	Polyclonal
EPHA7	H,M,R	Q15375	Polyclonal
EPHB1/2/3	H,M	P54762	Polyclonal
EPHB2	H	P29323	Polyclonal
EPHB4	H,M	P54760	Polyclonal
EPHB6	H,M,R	O15197	Polyclonal

EPN2	H,M,R	O95208	Polyclonal
EPN3	H	Q9H201	Polyclonal
ERAB	H,M,R	Q99714	Polyclonal
ERAS	H	Q7Z444	Polyclonal
ERCC1	H,M	P07992	Polyclonal
ERCC5	H	P28715	Polyclonal
ERCC6	H	Q03468	Polyclonal
ERF	H,M	P50548	Polyclonal
ERK2	H,M	P28482	Monoclonal
ERN1 (IRE1)	H	O75460	Monoclonal
Ezrin	H,M,R	P15311	Polyclonal
FA10 (activated heavy chain,Cleaved-Ile235)	H,M,R	P00742	Polyclonal
FA12 (heavy chain,Cleaved-Arg372)	H	P00748	Polyclonal
FA13A (Cleaved-Gly39)	H	P00488	Polyclonal
FA7 (light chain,Cleaved-Arg212)	H	P08709	Polyclonal
FABP4	H	P15090	Monoclonal
FADD	H	Q13158	Polyclonal
FAK	H	Q05397	Monoclonal
FAKD1	H,M	Q53R41	Polyclonal
FAKD2	H	Q9NYY8	Polyclonal
FAM84B	H	Q96KN1	Polyclonal
FAS	H	P25445	Polyclonal
FAS ligand	H,M	P48023	Polyclonal
FER	H,M,R	P16591	Polyclonal
Ferritin	H	P02794	Monoclonal
FES	H,M	P07332	Polyclonal
FGF-1	H	P05230	Polyclonal
FGF18	H	O76093	Polyclonal
FGF-2	H	P09038	Polyclonal
FGF22	H,M,R	Q9HCT0	Polyclonal
FGFR1 Oncogene Partner	H,M,R	O95684	Polyclonal
FGFR2	H,M	P21802	Polyclonal
FGFR3	H,M,R	P22607	Polyclonal
FGR	H,M,R	P09769	Polyclonal
FHIT	H	P49789	Polyclonal
Fibrillin-1	H,M,R	P35555	Polyclonal
Fibronectin	H	P02751	Monoclonal

Fibulin 5	H	Q9UBX5	Monoclonal
FKBPL	H,M,R	Q9UIM3	Polyclonal
FLI1	H,M	Q01543	Polyclonal
Flt-1 (VEGFR1)	H	P17948	Monoclonal
Flt3 ligand	H	P49771	Polyclonal
FMN2	H,M	Q9NZ56	Polyclonal
Fos	H,M,R	P01100	Polyclonal
FOXA2	H	Q9Y261	Monoclonal
FOXB1/2	H,M	Q99853	Polyclonal
FOXJ3	H,M	Q9UPW0	Polyclonal
FOXN4	H	Q96NZ1	Polyclonal
Foxp3	H,M	B7ZLG1	Monoclonal
FOXR1	H,M	Q6PIV2	Polyclonal
Fra-2	H,M,R	P15408	Polyclonal
Free PSA (KLK3)	H	P07288	Monoclonal
FRK	H,M,R	P42685	Polyclonal
FRS3	H	O43559	Polyclonal
FSH	H	P01225	Monoclonal
G3BP2	H,M,R	Q9UN86	Polyclonal
GABA-B Receptor	H,M,R	Q9UBS5	Polyclonal
GABRA6	H,M,R	Q16445	Polyclonal
GABRG1	H,M,R	Q8N1C3	Polyclonal
GAD1	H,M,R	Q99259	Polyclonal
GAD1/2	H,M	Q99259	Polyclonal
GADD153	H,M,R	P35638	Polyclonal
GADD45 beta	H,M,R	O75293	Polyclonal
GADD45GIP1	H	Q8TAE8	Polyclonal
Galectin 3	H,M,R	P17931	Polyclonal
Gamma-glutamyltransferase 4 (heavy chain,Cleaved-Thr472)	H,M,R	Q9UJ14	Polyclonal
GANP	H,M	O60318	Polyclonal
GAPDH	H,M,R	P04406	Monoclonal
GAS1	H,M	P54826	Polyclonal
GAS6	H	Q14393	Polyclonal
Gastrin	H,M,R	P01350	Polyclonal
GATA1	H	P15976	Monoclonal
GATA3	H	P23771	Monoclonal

GCNT7	H	Q6ZNI0	Polyclonal
GFR alpha-1	H,M,R	P56159	Polyclonal
GGH	H	Q92820	Polyclonal
GLB1L3	H	Q8NCI6	Polyclonal
Glucagon	H,M,R	P01275	Polyclonal
Glucose-6-phosphate isomerase	H	P06744	Monoclonal
GluR5	H,M,R	P39086	Polyclonal
GLUT1	H,M,R	P11166	Polyclonal
GLUT3	H	P11169	Polyclonal
GNL3L	H	Q9NVN8	Polyclonal
GNPAT	H	O15228	Polyclonal
GPR120	H,M,R	Q5NUL3	Polyclonal
GPR132	H,M	Q9UNW8	Polyclonal
GPR150	H	Q8NGU9	Polyclonal
GPR151	H	Q8TDV0	Polyclonal
GPR152	H	Q8TDT2	Polyclonal
GPR153	H,M	Q6NV75	Polyclonal
GPR160	H	Q9UJ42	Polyclonal
GPR171	H,M	O14626	Polyclonal
GPR173	H,M,R	Q9NS66	Polyclonal
GPR174	H,M	Q9BXC1	Polyclonal
GPR175	H,M,R	Q86W33	Polyclonal
GPR18	H	Q14330	Polyclonal
GPRIN1	H	Q7Z2K8	Polyclonal
GPRIN2	H	O60269	Polyclonal
GPRIN3	H	Q6ZVF9	Polyclonal
GRAH	H	P20718	Polyclonal
Granzyme B	H,M,R	P10144	Polyclonal
GRB14	H,M,R	Q14449	Polyclonal
GRB2	H,M,R	P62993	Polyclonal
GRID2	H,M,R	O43424	Polyclonal
GRK3	H,M,R	P35626	Polyclonal
GRK5	H,M,R	P34947	Polyclonal
GRK6	H,M,R	P43250	Polyclonal
GRK7	H	Q8WTQ7	Polyclonal
GRP75	H,M,R	P38646	Polyclonal
GRP78	H,M,R	P11021	Polyclonal

GRP94	H,M	P14625	Polyclonal
GRTP1	H	Q5TC63	Polyclonal
GSK3 alpha	H	P49840	Monoclonal
GSK3 beta	H,M,R	P49841	Monoclonal
Guanylate Cyclase beta	H,M,R	Q02153	Polyclonal
HAT	H,M,R	O14929	Polyclonal
Hcg	H	P01215/P01233	Monoclonal
HCK	H	P08631	Monoclonal
HDAC1	H,M,R	Q13547	Polyclonal
HDAC10	H,M,R	Q969S8	Polyclonal
HDAC3	H,M,R	O15379	Polyclonal
HDAC5	H,M,R	Q9UQL6	Polyclonal
HDAC6	H,M	Q9UBN7	Polyclonal
HDAC7	H,M	Q8WUI4	Polyclonal
HDAC9	H	Q9UKV0	Polyclonal
HEN1/2	H,M	Q02575	Polyclonal
Hepatitis B Surface Antigen	H	N/A	Monoclonal
HER2	H,M,R	P04626	Polyclonal
HER3	H	P21860	Polyclonal
Heregulin	H,M,R	Q15491	Polyclonal
Hexokinase-3	H	P52790	Polyclonal
HGH	H	P01241	Monoclonal
HIPK4	H,M,R	Q8NE63	Polyclonal
Histone 1F0	H	P07305	Polyclonal
Histone H1 (Acetyl-Lys25)	H	Q8IZA3	Polyclonal
Histone H2A (Acetyl-Lys5)	H,M,R	P0C0S5	Polyclonal
Histone H2AX	H	P16104	Polyclonal
Histone H2B	H,M	Q93079	Polyclonal
Histone H2B (Acetyl-Lys12)	H,M	P57053	Polyclonal
Histone H2B (Acetyl-Lys15)	H,M	P57053	Polyclonal
Histone H2B (Acetyl-Lys5)	H,M	P57053	Polyclonal
Histone H3 (Acetyl-Lys18)	H,M,R	P68431	Polyclonal
Histone H3 (Acetyl-Lys23)	H,M,R	P68431	Polyclonal
Histone H3 (Acetyl-Lys27)	H,M,R	P68431	Polyclonal
Histone H3 (Acetyl-Lys9)	H,M,R	P68431	Polyclonal
Histone H4 (Acetyl-Lys12)	H,M,R	P62805	Polyclonal
Histone H4 (Acetyl-Lys5)	H,M,R	P62805	Polyclonal

Histone H4 (Acetyl-Lys8)	H,M,R	P62805	Polyclonal
HLA-DOA	H	P06340	Polyclonal
HLAH	H	P01893	Polyclonal
HMG20B	H	Q9P0W2	Polyclonal
HMGB1	H,M,R	P09429	Polyclonal
HMGB2	H,M,R	P26583	Polyclonal
HNF4alpha/gamma	H,M,R	P41235/Q14541	Polyclonal
HOXA11/D11	H,M	P31270	Polyclonal
HOXA6	H,M	P31267	Polyclonal
HOXB2	H	P14652	Polyclonal
HPRT	H	P00492	Monoclonal
HSF2	H,M,R	Q03933	Polyclonal
HSP10	H,M,R	P61604	Polyclonal
HSP105	H,M	Q92598	Polyclonal
HSP40	H,M,R	P25685	Polyclonal
HSP60	H,M,R	P10809	Polyclonal
HSP90A	H,M,R	P07900	Polyclonal
HSPB2	H,M,R	Q16082	Polyclonal
human Albumin	H	P02768	Monoclonal
ICAM1	H	P05362	Monoclonal
IFN-gamma	H	P01579	Monoclonal
IgA	H	P01876	Monoclonal
IgE	H	P01854	Monoclonal
IGF 1R	H	P08069	Monoclonal
IGFBP2	H	P18065	Monoclonal
IgG	H	P01860	Monoclonal
IGLL1	H	P15814	Polyclonal
IgM	H	P01871	Monoclonal
Ik3-2	H,M	Q9BTV7	Polyclonal
IKB alpha	H,M,R	P25963	Polyclonal
IKBKB (IKK beta)	H	O14920	Monoclonal
IKBKE (IKK epsilon)	H	Q14164	Monoclonal
IKK Alpha	H	O15111	Monoclonal
IKK-gamma	H	Q9Y6K9	Polyclonal
IL-1 alpha	H	P01583	Monoclonal
IL-1 beta	H	P01584	Monoclonal
IL-10	H	P22301	Monoclonal

IL-1beta (Cleaved-Asp210)	H	P29466	Polyclonal
IL-2	H	P60568	Monoclonal
IL20RB	H,M	Q6UXL0	Polyclonal
IL-6	H	P05231	Monoclonal
IL-8	H	P10145	Monoclonal
ILKAP	H,M,R	Q9H0C8	Polyclonal
Influenza B virus Nucleoprotein	H	P04665	Monoclonal
INHA (Inhibin alpha)	H	P05111	Monoclonal
iNOS	H	P35228	Polyclonal
INSL4	H	Q14641	Polyclonal
Insulin	H	P01308	Monoclonal
Integrin beta5	H,M,R	P18084	Polyclonal
Involucrin	H	P07476	Polyclonal
IP3KA	H,M,R	P23677	Polyclonal
IP3KC	H	Q96DU7	Polyclonal
IP6K2	H,M,R	Q9UHH9	Polyclonal
IP6K3	H,M	Q96PC2	Polyclonal
IPKA	H,M,R	P61925	Polyclonal
IPKB	H,M,R	Q9C010	Polyclonal
IRAK3	H	Q9Y616	Polyclonal
IRF4	H,M	Q15306	Polyclonal
ITGA5	H	P08648	Monoclonal
ITGA5 (heavy chain,Cleaved-Phe42)	H,M	P08648	Polyclonal
ITGA5 (light chain,Cleaved-Glu874)	H	P08648	Polyclonal
ITGA6 (light chain,Cleaved-Glu942)	H	P23229	Polyclonal
ITGA7 (light chain,Cleaved-Glu959)	H	Q13683	Polyclonal
ITGAV (heavy chain,Cleaved-Lys889)	H	P06756	Polyclonal
ITIH1 (Cleaved-Asp672)	H,M	P19827	Polyclonal
ITK (LYK)	H	Q08881	Monoclonal
JAB1	H,M,R	Q9UNS2	Polyclonal
JAK1	H,M,R	P23458	Polyclonal
JAK2	H	O60674	Monoclonal
JAK3	H,M	P52333	Monoclonal
JM4	H,M,R	O60831	Polyclonal
JNKK	H,M,R	P45985	Polyclonal
Kallikrein-11 (Cleaved-Ile54)	H	Q9UBX7	Polyclonal
KAP0	H,M,R	P10644	Polyclonal

KCNA1	H,M,R	Q09470	Polyclonal
KCND1	H,M	Q9NSA2	Polyclonal
KCNG3	H,M,R	Q8TAE7	Polyclonal
KCNJ2	H,R	P63252	Polyclonal
KCNJ9	H,M,R	Q92806	Polyclonal
KCNK15	H	Q9H427	Polyclonal
KCNK17	H	Q96T54	Polyclonal
KCNK4	H,M	Q9NYG8	Polyclonal
KCNMB2	H,M,R	Q9Y691	Polyclonal
KCNT1	H,M,R	Q5JUK3	Polyclonal
KCNV2	H,M	Q8TDN2	Polyclonal
KDR (VEGFR2)	H	P35968	Monoclonal
Keratin 1 (CK1)	H	P04264	Monoclonal
Keratin 10	H,M,R	P13645	Polyclonal
Keratin 15	H,M,R	P19012	Polyclonal
Keratin 16	H,M,R	P08779	Polyclonal
Keratin 17	H,M,R	Q04695	Polyclonal
Keratin 18	H,M,R	P05783	Polyclonal
Keratin 19	H,M,R	P08727	Polyclonal
Keratin 20	H,M,R	P35900	Polyclonal
Keratin 5	H,M,R	P13647	Polyclonal
Keratin 7	H	P08729	Polyclonal
Keratin 8	H,M,R	P05787	Polyclonal
Ki67	H	P46013	Polyclonal
KIAA1967	H	Q8N163	Polyclonal
KIF4A	H	O95239	Polyclonal
KITH	H	P04183	Polyclonal
KLHL3	H	Q9UH77	Polyclonal
KLKB1 (heavy chain,Cleaved-Arg390)	H	P03952	Polyclonal
KPB1/2	H,M,R	P46020	Polyclonal
KSR2	H,M	Q6VAB6	Polyclonal
Ku70	H,M,R	P12956	Polyclonal
Ku70/80	H	P13010	Polyclonal
LAMA1	H	P25391	Polyclonal
LAMA3	H,M,R	Q16787	Polyclonal
LAMA4	H,M	Q16363	Polyclonal
LAMA5	H,M	O15230	Polyclonal

LAMB2	H,M,R	P55268	Polyclonal
LAMB3	H	Q13751	Polyclonal
LAMC3	H,M	Q9Y6N6	Polyclonal
Lamin A (Cleaved-Asp230)	H,M,R	P02545	Polyclonal
Laminin	H	Q9Y6N6	Monoclonal
LAMP3	H	Q9UQV4	Polyclonal
LAT3	H	O75387	Polyclonal
LATH	H	Q86YQ2	Polyclonal
Lck	H	P06239	Monoclonal
LDLRAD1	H	Q5T700	Polyclonal
LDLRAD2	H	Q5SZI1	Polyclonal
LDLRAD3	H,M	Q86YD5	Polyclonal
LDOC1L	H,M	Q6ICC9	Polyclonal
LEG4	H	P56470	Polyclonal
LEG7	H,M,R	P47929	Polyclonal
LEG9	H,M,R	O00182	Polyclonal
LH	H	P01229	Monoclonal
LHR2A	H	O00534	Polyclonal
LILRA1	H	O75019	Polyclonal
LILRA2	H	Q8N149	Polyclonal
LIMK1	H,M,R	P53667	Polyclonal
LIMK2	H,M,R	P53671	Polyclonal
LPA	H	P08519	Monoclonal
LPL	H	P06858	Monoclonal
LRP10	H,M,R	Q7Z4F1	Polyclonal
LRP11	H	Q86VZ4	Polyclonal
LRP3	H,M,R	O75074	Polyclonal
LRRK1	H	Q38SD2	Polyclonal
LW-1	H	Q9UBD0	Polyclonal
LYN	H	P07948	Monoclonal
Lys-acetylated proteins	H,M,R	N/A	Polyclonal
M3K13	H,M,R	O43283	Polyclonal
MADD	H,M,R	Q8WXG6	Polyclonal
MAGE-1	H	P43355	Polyclonal
Mammaglobin	H,R	Q13296	Polyclonal
Mammaglobin B	H	O75556	Polyclonal
MAP2K2 (MEK2)	H,M,R	P36507	Monoclonal

MAP2K4	H	P45985	Monoclonal
MAP2K6	H,M,R	P52564	Polyclonal
MAP3K1	H,M,R	Q13233	Polyclonal
MAP3K10	H,M	Q02779	Polyclonal
MAP3K3	H,M	Q99759	Polyclonal
MAP3K4	H,M	Q9Y6R4	Polyclonal
MAP3K6	H,M	O95382	Polyclonal
MAP3K9	H,M	P80192	Polyclonal
MAP3KL4	H	Q5TCX8	Polyclonal
MAP4K3	H,M,R	Q8IVH8	Polyclonal
MAP4K4	H	O95819	Monoclonal
MAP4K6	H,M	Q8N4C8	Polyclonal
MAPK 11	H	Q15759	Monoclonal
MAPK10	H,M,R	P53779	Polyclonal
MAPK15	H	Q8TD08	Polyclonal
MAPK3	H,M,R	Q16644	Polyclonal
MAPK9	H,M,R	P45984	Polyclonal
March2	H,M,R	Q9P0N8	Polyclonal
March3	H,M,R	Q86UD3	Polyclonal
March4	H,M	Q9P2E8	Polyclonal
March5	H,M,R	Q9NX47	Polyclonal
MARK	H,M,R	Q9P0L2	Polyclonal
MARK2	H,M,R	Q7KZI7	Polyclonal
MARK3	H,M,R	P27448	Polyclonal
MARK4	H,M	Q96L34	Polyclonal
MART-1	H	Q16655	Polyclonal
MASP1 (heavy chain,Cleaved-Arg448)	H	P48740	Polyclonal
MAST3	H,M	O60307	Polyclonal
MAST4	H,M	O15021	Polyclonal
MAT1	H,M,R	P51948	Polyclonal
MATK (CTK)	H	P42679	Monoclonal
MCL-1	H	Q07820	Monoclonal
MCL1	H,M,R	Q07820	Polyclonal
MCM2	H,M	P49736	Polyclonal
MCM5	H,M	P33992	Polyclonal
MDFI	H,M	Q99750	Polyclonal
MDM2	H,M	Q00987	Polyclonal

MDM4	H	O15151	Monoclonal
ME1	H,M,R	P48163	Polyclonal
ME3	H,M	Q16798	Polyclonal
MEF2B	H,M	Q02080	Polyclonal
MEF2C	H,M	Q06413	Polyclonal
MEKKK 1	H,M	Q92918	Polyclonal
MEKKK 4	H,M	O95819	Polyclonal
MER	H	Q12866	Monoclonal
MET	H	P08581	Polyclonal
Mevalonate Kinase	H	Q03426	Polyclonal
mGluR2/3	H,M,R	Q14416	Polyclonal
mGluR4	H,M,R	Q14833	Polyclonal
mGluR6	H	O15303	Polyclonal
mGluR7	H,M,R	Q14831	Polyclonal
mGluR8	H,M,R	O00222	Polyclonal
MGMT	H,R	P16455	Polyclonal
MIPT3	H	Q8TDR0	Polyclonal
MLH1	H,M,R	P40692	Polyclonal
MLH3	H	Q9UHC1	Polyclonal
MLL	H	Q03164	Monoclonal
Mlx	H,M,R	Q9UH92	Polyclonal
MMP-1	H	P03956	Polyclonal
MMP1 (Cleaved-Phe100)	H	P03956	Polyclonal
MMP-10	H,M,R	P09238	Polyclonal
MMP-11	H,M,R	P24347	Polyclonal
MMP12 (Cleaved-Glu106)	H	P39900	Polyclonal
MMP-13	H	P45452	Polyclonal
MMP-14	H,M,R	P50281	Polyclonal
MMP14 (Cleaved-Tyr112)	H,M,R	P50281	Polyclonal
MMP-15	H,M	P51511	Polyclonal
MMP15 (Cleaved-Tyr132)	H	P51511	Polyclonal
MMP-16	H,M,R	P51512	Polyclonal
MMP17 (Cleaved-Gln129)	H	Q9ULZ9	Polyclonal
MMP-19	H,M	Q99542	Polyclonal
MMP-2	H,M,R	P08253	Polyclonal
MMP-23	H,M,R	O75900	Polyclonal
MMP23 (Cleaved-Tyr79)	H,M,R	O75900	Polyclonal

MMP27 (Cleaved-Tyr99)	H	Q9H306	Polyclonal
MMP-3	H,M,R	P08254	Polyclonal
MMP3 (Cleaved-Phe100)	H	P08254	Polyclonal
MMP-7	H,M,R	P09237	Polyclonal
MMP-8	H,M,R	P22894	Polyclonal
MMP-9	H	P14780	Polyclonal
MOK	H	Q9UQ07	Polyclonal
MPRIP	H,M,R	Q6WCQ1	Polyclonal
MPS1	H	P42677	Monoclonal
MRC2	H,M,R	Q9UBG0	Polyclonal
MRCKB	H,M,R	Q9Y5S2	Polyclonal
MRP9	H	Q96J65	Polyclonal
MSH2	H,M,R	P43246	Polyclonal
MSH3	H	P20585	Polyclonal
MSH6	H,M	P52701	Polyclonal
MTA1	H,M,R	Q13330	Polyclonal
MUC13	H	Q9H3R2	Polyclonal
Mucin-14	H,M,R	Q9ULC0	Polyclonal
MUM1	H	Q2TAK8	Monoclonal
MUSK	H	O15146	Monoclonal
MUTYH	H,M,R	Q9UIF7	Polyclonal
MYBPC3	H,M	Q14896	Polyclonal
MYC	H,M,R	P01106	Polyclonal
Myeloperoxidase	H	P05164	Monoclonal
MYH14	H,M	Q7Z406	Polyclonal
MYH4	H	Q9Y623	Polyclonal
MYL2	H	P10916	Monoclonal
MYL3	H	P08590	Monoclonal
MYLIP	H,M	Q8WY64	Polyclonal
MYO1D	H,M,R	O94832	Polyclonal
Myoglobin	H	P02144	Monoclonal
MYOM1	H	P52179	Polyclonal
MYOM2	H,M	P54296	Polyclonal
Myostatin	H	O14793	Monoclonal
MYST1	H	Q9H7Z6	Monoclonal
NCK2	H,M	O43639	Polyclonal
NCOA3	H	Q9Y6Q9	Monoclonal

NCOA7	H	Q8NI08	Polyclonal
NCoR1	H,M	O75376	Polyclonal
NCR1	H	O76036	Polyclonal
NCR3	H,R	O14931	Polyclonal
NEDD8	H,M,R	Q15843	Polyclonal
NEGR1	H,M,R	Q7Z3B1	Polyclonal
Neurogenin-3	H	Q9Y4Z2	Monoclonal
Neuropsin (Cleaved-Val33)	H	O60259	Polyclonal
NF1	H,M,R	P21359	Polyclonal
NF-kB p65	H,M	Q04206	Monoclonal
NFRKB	H,M	Q6P4R8	Polyclonal
NFYC	H,M,R	Q13952	Polyclonal
NF-κB p65 (Acetyl-Lys310)	H,M	Q04206	Polyclonal
NGFR	H	P08138	Monoclonal
NKX2.5	H	P52952	Monoclonal
NKX26	H	A6NCS4	Polyclonal
NKX3.1	H	Q99801	Polyclonal
NKX3A	H	Q99801	Monoclonal
NM23	H,M,R	P22392	Polyclonal
Notch 1 (Cleaved-Val1754)	H,M,R	P46531	Polyclonal
Notch 2 (Cleaved-Ala1734)	H,M,R	Q04721	Polyclonal
Notch 2 (Cleaved-Asp1733)	H,M,R	Q04721	Polyclonal
Notch 2 (Cleaved-Val1697)	H,M,R	Q04721	Polyclonal
NOX3	H,M,R	Q9HBY0	Polyclonal
NOX5	H	Q96PH1	Polyclonal
NPTN	H,M,R	Q9Y639	Polyclonal
NRBF2	H,M,R	Q96F24	Polyclonal
Nrf2	H,M,R	Q16236	Polyclonal
NSE	H,M,R	P09104	Polyclonal
NSG1	H,M,R	P42857	Polyclonal
NSG2	H,M	Q9Y328	Polyclonal
NT	H,M,R	Q9P121	Polyclonal
Nucleophosmin (NPM)	H	Q9BYG9	Monoclonal
NYREN18	H,M	Q9Y5A7	Polyclonal
Oct-1	H,M	P14859	Polyclonal
Oct-2	H,M	P09086	Polyclonal
Oct-3	H,M,R	Q01860	Polyclonal

Oct-6	H,M,R	Q03052	Polyclonal
ORAV1	H	Q8WV07	Polyclonal
ORCTL-2	H	Q96BI1	Polyclonal
Osteopontin	H,M,R	P10451	Polyclonal
p14 ARF	H	Q8N726	Polyclonal
p15 INK	H,M,R	P42772	Polyclonal
p16 INK	H,M	P42771	Polyclonal
p18 INK	H,M	P42773	Polyclonal
p19 INK4d	H	P55273	Polyclonal
p300	H	Q09472	Polyclonal
p300/CBP	H,M,R	Q92831	Polyclonal
p42 MAPK	H,M,R	P28482	Polyclonal
p44 MAPK	H,M,R	P27361	Polyclonal
p44/42 MAPK	H,M,R	P27361	Polyclonal
p50 CDC37	H,M,R	Q16543	Polyclonal
p50 Dynamitin	H,M,R	Q13561	Polyclonal
p53	H,M,R	P04637	Polyclonal
p53 (Acetyl-Lys386)	H	P04637	Polyclonal
p55CDC	H,M,R	Q12834	Polyclonal
p57KIP2	H	P49918	Polyclonal
p63	H,M,R	Q9H3D4	Polyclonal
p73	H	O15350	Polyclonal
p97 MAPK	H,M,R	Q16659	Polyclonal
PAK2	H	Q13177	Monoclonal
PAR1	H	P25116	Monoclonal
PAR4	H	Q96IZ0	Monoclonal
PAR4 (Cleaved-Gly48)	H	Q96RI0	Polyclonal
Parathyroid Hormone	H	P01270	Polyclonal
PARK7	H,M,R	Q99497	Polyclonal
Parkin	H,M,R	O60260	Polyclonal
PARL	H	Q9H300	Monoclonal
PARP	H	P09874	Monoclonal
PARP (Cleaved-Asp214)	H,M	P09874	Polyclonal
PARP (Cleaved-Gly215)	H	P09874	Polyclonal
PARP3	H	Q9Y6F1	Polyclonal
Patched	H,M	Q13635	Polyclonal
Pax-5	H,M	Q02548	Polyclonal

PBOV1	H	Q9GZY1	Polyclonal
PC	H,M,R	P11498	Polyclonal
PCNA	H,M,R	P12004	Polyclonal
PDGFB	H,M,R	P01127	Polyclonal
PDGFR alpha	H,M,R	P16234	Polyclonal
PDGFR beta	H,M	P09619	Monoclonal
PDK2	H,M,R	Q15119	Polyclonal
PDRG1	H,M,R	Q9NUG6	Polyclonal
PE2R3	H	P43115	Polyclonal
PE2R4	H	P35408	Polyclonal
Peripherin	H,R	P41219	Polyclonal
PGP9.5	H,M,R	P09936	Polyclonal
PHLA1	H,M,R	Q8WV24	Polyclonal
PIAS1	H,M	O75925	Polyclonal
PIAS2	H,M,R	O75928	Polyclonal
PIAS3	H,M,R	Q9Y6X2	Polyclonal
PIAS4	H,M	Q8N2W9	Polyclonal
PIGH	H,M	Q14442	Polyclonal
PIGY	H,M	Q3MUY2	Polyclonal
PIK3R5	H,M	Q8WYR1	Polyclonal
PIP5K	H,M	Q9Y2I7	Polyclonal
PIP5K1C	H	O60331	Polyclonal
Pirh2 (RCHY1)	H,R	Q96PM5	Monoclonal
PKCB1	H	Q9ULU4	Polyclonal
PKM2	H	P14618	Polyclonal
PLA1A	H	Q53H76	Polyclonal
PLA2G4C	H	Q9UP65	Polyclonal
PLA2G4D	H	Q86XP0	Polyclonal
PLA2G4E	H	Q3MJ16	Polyclonal
PLD4	H,M,R	Q96BZ4	Polyclonal
PLK2	H,M,R	Q9NYY3	Polyclonal
PLK3	H,M,R	Q9H4B4	Polyclonal
PLK5	H	Q496M5	Polyclonal
PML	H	P29590	Polyclonal
PMP22	H,M,R	Q01453	Polyclonal
PMS2/PMS2CL	H	Q68D20	Polyclonal
POLD3	H,M,R	Q15054	Polyclonal

POLDIP3	H,M	Q9BY77	Polyclonal
POLE1	H,M	Q07864	Polyclonal
POLG2	H,M	Q9UHN1	Polyclonal
POLI	H	Q9UNA4	Polyclonal
Potassium Channel Kv3.2b	H,M,R	Q96PR1	Polyclonal
POTE8	H	Q6S8J7	Polyclonal
PPGB (32k,Cleaved-Arg326)	H,M	P10619	Polyclonal
PPHLN	H,M	Q8NEY8	Polyclonal
PPP1R8	H,M,R	Q12972	Polyclonal
PPRC1	H,M	Q5VV67	Polyclonal
Presenilin 1	H	P49768	Polyclonal
PRIM1	H,M,R	P49642	Polyclonal
PRKAB1	H,M,R	Q9Y478	Polyclonal
PRKX	H,M,R	P51817	Polyclonal
PRKY	H	O43930	Polyclonal
Prolactin	H	P01236	Monoclonal
Prostate Apoptosis Response protein-4	H,M,R	Q96IZ0	Polyclonal
Prostate Stem Cell Antigen	H,M	O43653	Polyclonal
Prostate-specific Antigen	H	P07288	Polyclonal
Proteinase 3	H	P24158	Monoclonal
PROZ	H	P22891	Monoclonal
PRPF19	H,M,R	Q9UMS4	Polyclonal
PSA-ACT	H	P07288	Monoclonal
PTEN	H,M	P60484	Monoclonal
PTH (Parathyroid Hormone)	H	P01270	Monoclonal
PTK6 (breast tumor kinase)	H	Q13882	Monoclonal
PTTG1	H	O95997	Polyclonal
PYK2 (FAK2)	H	Q14289	Monoclonal
QSK	H,M	Q9Y2K2	Polyclonal
RAB11FIP2	H,M	Q7L804	Polyclonal
RAB11FIP3	H,M,R	O75154	Polyclonal
RAB11FIP4	H,M	Q86YS3	Polyclonal
RAB18	H,M,R	Q9NP72	Polyclonal
RAB20	H	Q9NX57	Polyclonal
Rab25	H	P57753	Monoclonal
RAB34	H	Q9BZG1	Polyclonal
RAB37	H,M	Q96AX2	Polyclonal

RAB38	H,M,R	P57729	Polyclonal
RAB3GAP1	H	Q15042	Polyclonal
RAB3GAP2	H,M,R	Q9H2M9	Polyclonal
RAB40B	H	Q12829	Polyclonal
RAB41	H	Q5JT25	Polyclonal
RAB5C	H,M,R	P51148	Polyclonal
RAB6A	H,M,R	P20340	Polyclonal
RAB6C	H,M,R	Q9H0N0	Polyclonal
RAB7L1	H,M,R	O14966	Polyclonal
RABEP1	H,M,R	Q15276	Polyclonal
RABEP2	H,M,R	Q9H5N1	Polyclonal
RAD50	H,M,R	Q92878	Polyclonal
RAD51L1	H,M	O15315	Polyclonal
RAN	H,M,R	P62826	Polyclonal
RASH/RASK	H,M,R	P01112	Polyclonal
RASSF2	H,M,R	P50749	Polyclonal
RASSF4	H,M,R	Q9H2L5	Polyclonal
RASSF6	H	Q6ZTQ3	Polyclonal
RBAK	H,M	Q9NYW8	Polyclonal
RBM26	H,M	Q5T8P6	Polyclonal
RBM5	H,M	P52756	Polyclonal
RCBTB1	H,M	Q8NDN9	Polyclonal
RCL	H	O43598	Polyclonal
RDX	H,M,R	P35241	Polyclonal
RED	H,M,R	Q13123	Polyclonal
REN	H	P00797	Polyclonal
RET	H	P07949	Monoclonal
Retinoic Acid Receptor beta	H,M	P10826	Polyclonal
Retinoid X Receptor gamma	H,M	P48443	Polyclonal
RFPL4A	H	A6NLU0	Polyclonal
RFWD2	H,M	Q8NHY2	Polyclonal
RGS1	H,M,R	Q08116	Polyclonal
RHG17	H,M,R	Q68EM7	Polyclonal
RHG22	H	Q7Z5H3	Polyclonal
RHG9	H	Q9BRR9	Polyclonal
RHOBTB3	H,M	O94955	Polyclonal
RhoH	H,M,R	Q15669	Polyclonal

RIT1	H,M	Q92963	Polyclonal
RON	H	Q04912	Monoclonal
ROR1	H	Q01973	Monoclonal
RORA	H	P35398	Polyclonal
RPC1	H,M	O14802	Polyclonal
RPC4	H,M	P05423	Polyclonal
RPC8	H,M,R	Q9Y535	Polyclonal
RSK1 (p90 RSK)	H	Q15418	Monoclonal
R-spondin 1	H	Q2MKA7	Monoclonal
RTN3	H	O95197	Monoclonal
S100 A1	H,M,R	P23297	Polyclonal
S100A10/ P11	H	P60903	Monoclonal
S100A16	H,R	Q96FQ6	Polyclonal
S100A3	H,M,R	P33764	Polyclonal
S100B	H	P04271	Monoclonal
S100Z	H	Q8WXG8	Polyclonal
S6K	H,M,R	P23443	Polyclonal
S6K-alpha2	H,M	Q15349	Polyclonal
S6K-alpha6	H	Q9UK32	Polyclonal
SAA4	H	P35542	Polyclonal
SAR1B	H,M,R	Q9Y6B6	Polyclonal
SDCG1	H,M,R	O60524	Polyclonal
SENP1	H,M	Q9P0U3	Polyclonal
SENP2	H,M,R	Q9HC62	Polyclonal
SENP3	H,M	Q9H4L4	Polyclonal
SENP5	H,M	Q96HI0	Polyclonal
SENP6	H	Q9GZR1	Polyclonal
SENP7	H,M	Q9BQF6	Polyclonal
SENP8	H,M,R	Q96LD8	Polyclonal
Septin-1	H,M,R	Q8WYJ6	Polyclonal
Septin-2	H,M,R	Q15019	Polyclonal
Septin-3	H,M,R	Q9UH03	Polyclonal
Septin-7	H,M,R	Q16181	Polyclonal
Septin-8	H,M,R	Q92599	Polyclonal
SERC1	H,M,R	Q9NRX5	Polyclonal
SERC2	H,M,R	Q96SA4	Polyclonal
SERC3	H,M,R	Q13530	Polyclonal

Serpin A5	H	P05154	Polyclonal
SERPINB7	H	O75635	Polyclonal
SERPINB9	H,M,R	P50453	Polyclonal
SESN1	H,M,R	Q9Y6P5	Polyclonal
SGOL1	H	Q5FBB7	Polyclonal
SHC2	H,R	P98077	Polyclonal
SHC3	H,M,R	Q92529	Polyclonal
SHD	H	Q96IW2	Polyclonal
SHIP1	H,M,R	Q92835	Polyclonal
SH-PTP2	H,M,R	Q06124	Polyclonal
SIAH1	H,M,R	Q8IUQ4	Polyclonal
SIAH2	H,M,R	O43255	Polyclonal
Sirp alpha1	H,M,R	P78324	Polyclonal
SIRPB1	H	O00241	Polyclonal
SIRPG	H	Q9P1W8	Polyclonal
SIX5	H,M	Q8N196	Polyclonal
SKP1A/p19	H,M,R	P63208	Polyclonal
SKP2/p45	H	Q13309	Polyclonal
SLC17A2	H,M	O00624	Polyclonal
SLC24A4	H,M,R	Q8NFF2	Polyclonal
SLC24A6	H,M	Q6J4K2	Polyclonal
SLC25A21	H,M,R	Q9BQT8	Polyclonal
SLC25A31	H,M	Q9H0C2	Polyclonal
SLC25A6	H	P12236	Polyclonal
SLC27A4	H,M	Q6P1M0	Polyclonal
SLC27A5	H	Q9Y2P5	Polyclonal
SLC28A2	H,R	O43868	Polyclonal
SLC30A1	H	Q9Y6M5	Polyclonal
SLC30A4	H,M,R	O14863	Polyclonal
SLC30A8	H	Q8IWU4	Polyclonal
SLC39A1	H	Q9NY26	Polyclonal
SLC39A7	H,M	Q92504	Polyclonal
SLC4A11	H	Q8NBS3	Polyclonal
SLC4A8/10	H,M,R	Q6U841	Polyclonal
SLC5A2	H,M,R	P31639	Polyclonal
SLC5A3	H,M,R	P53794	Polyclonal
SLC5A6	H	Q9Y289	Polyclonal

SLC6A15	H,R	Q9H2J7	Polyclonal
SLC6A16	H	Q9GZN6	Polyclonal
SLC6A6	H,M,R	P31641	Polyclonal
SLC9A7	H,M	Q96T83	Polyclonal
SLC9A9	H,M	Q8IVB4	Polyclonal
SLCO1A2	H	P46721	Polyclonal
SLK	H,M,R	Q9H2G2	Polyclonal
SLU7	H,M,R	O95391	Polyclonal
Smad1/5/9	H,M,R	Q15797/Q99717/O15198	Polyclonal
Smad4	H,M,R	Q13485	Polyclonal
SMF	H	Q12766	Polyclonal
SNAI2 (SLUG)	H	O43623	Monoclonal
SNAP25	H,M,R	P60880	Polyclonal
SNCA (alpha-synuclein)	H	P37840	Monoclonal
SND1/P100	H	Q7KZF4	Monoclonal
SOD1	H,M	P00441	Monoclonal
Sodium Channel-pan	H,M,R	P35498	Polyclonal
Somatostatin	H,M,R	P61278	Polyclonal
SORL1	H	Q92673	Monoclonal
SOX2	H	P48431	Monoclonal
SP3/4	H,M,R	Q02447	Polyclonal
SPINK6	H	Q6UWN8	Polyclonal
SPR1	H,M	Q15743	Polyclonal
SPTA2 (Cleaved-Asp1185)	H	Q13813	Polyclonal
SPTBN1	H,M	Q01082	Polyclonal
SPTBN5	H	Q9NRC6	Polyclonal
SRA	H	Q9HD15	Monoclonal
SRC	H	P12931	Monoclonal
SRPK1	H,M,R	Q96SB4	Polyclonal
SRY	H	Q05066	Polyclonal
ST5	H,M	P78524	Polyclonal
ST6GAL1	H,M,R	P15907	Polyclonal
STAG3	H	Q9UJ98	Polyclonal
STAT1	H	P42224	Polyclonal
STAT3	H,M,R	P40763	Polyclonal
STAT5A	H,M,R	P42229	Polyclonal
STAT5A/B	H,M,R	P42229	Polyclonal

STEA2	H,M	Q8NFT2	Polyclonal
STEA3	H,M,R	Q658P3	Polyclonal
STEAP4	H	Q687X5	Polyclonal
Stefin A	H	P01040	Polyclonal
Stefin B	H,R	P04080	Polyclonal
STK24	H,M,R	Q9Y6E0	Polyclonal
STK36	H,M	Q9NRP7	Polyclonal
STK39	H,M,R	Q9UEW8	Polyclonal
STMN4	H,M,R	Q9H169	Polyclonal
STRAD	H,M	Q7RTN6	Polyclonal
STYK1	H	Q6J9G0	Monoclonal
Sumo1	H,M,R	P63165	Polyclonal
SUMO2/3 (Cleaved-Gly93)	H,M,R	P61956	Polyclonal
Survivin	H	O15392	Monoclonal
SVOP	H,M,R	Q8N4V2	Polyclonal
SYK	H	P43405	Polyclonal
Synaptophysin	H,M,R	P08247	Polyclonal
Synuclein beta	H,M,R	Q16143	Polyclonal
Synuclein gamma	H	O76070	Polyclonal
Synuclein-pan	H,M,R	P37840	Polyclonal
TACC1	H,M	O75410	Polyclonal
TACD1	H	P16422	Polyclonal
TAF15	H,M	Q92804	Polyclonal
TAF1A	H,R	Q15573	Polyclonal
TAF4	H,M	O00268	Polyclonal
TAF5	H,M	Q15542	Polyclonal
TAF5L	H	O75529	Polyclonal
TAF6L	H,M	Q9Y6J9	Polyclonal
TALL-2	H,M,R	O75888	Polyclonal
TBP	H,M,R	P20226	Polyclonal
TENS3	H	Q68CZ2	Polyclonal
Testosterone	H	P04278	Monoclonal
TGF alpha	H,M,R	P01135	Polyclonal
TGF beta Receptor II	H,M,R	P37173	Polyclonal
TGF beta Receptor III	H,M,R	Q03167	Polyclonal
TGF beta1	H,M,R	P01137	Polyclonal
TGF beta2	H,M,R	P61812	Polyclonal

TGF beta3	H,M,R	P10600	Polyclonal
Thioredoxin (TRX)	H	P10599	Monoclonal
THRB (AP2,Cleaved-Arg327)	H	P00734	Polyclonal
Thrombin Receptor	H	P25116	Polyclonal
Thyroid Hormone Receptor alpha	H,M,R	P10827	Polyclonal
Thyroid Hormone Receptor beta	H,M,R	P10828	Polyclonal
TIMP1	H,M,R	P01033	Polyclonal
TIMP2	H,M,R	P16035	Polyclonal
TIMP3	H,M,R	P35625	Polyclonal
TIMP4	H,M,R	Q99727	Polyclonal
TLE2	H	Q04725	Polyclonal
TLE4	H,M,R	Q04727	Polyclonal
TNF Receptor I	H,M,R	P19438	Polyclonal
TNF Receptor II	H,M,R	P20333	Polyclonal
TNF11	H,M	O14788	Polyclonal
TNF12	H,M	O43508	Polyclonal
TNF14	H	O43557	Polyclonal
TNFA	H,M,R	P01375	Polyclonal
TNFL4	H	P23510	Polyclonal
TNK1	H	Q13470	Monoclonal
TNNI3K	H,M,R	Q59H18	Polyclonal
TNXB	H,M	P22105	Polyclonal
TOP2A	H	P11388	Polyclonal
TOP2B	H,M	Q02880	Polyclonal
TOP3B	H,M	O95985	Polyclonal
Total PSA	H	P07288	Monoclonal
TP53I11	H	O14683	Polyclonal
TP53INP1	H	Q96A56	Polyclonal
TP53INP2	H,M,R	Q8CFU8	Polyclonal
TPD52	H,M	P55327	Polyclonal
TPD54	H,M,R	O43399	Polyclonal
TRADD	H,M	Q15628	Polyclonal
Transglutaminase 2	H,M	P21980	Polyclonal
TrkA	H	P04629	Monoclonal
TRXR2	H,M,R	Q9NNW7	Polyclonal
TSH	H	P01222	Monoclonal
TSH1	H,M	Q6ZSZ6	Polyclonal

TSH2	H,M	Q9NRE2	Polyclonal
TTF2	H,M,R	O00358	Polyclonal
TUBB3 (Tubulin beta 3)	H	Q13509	Monoclonal
TUBGCP3	H,M	Q96CW5	Polyclonal
TUBGCP4	H,M	Q9UGJ1	Polyclonal
TUBGCP5	H,M	Q96RT8	Polyclonal
TUBGCP6	H	Q96RT7	Polyclonal
Tubulin alpha	H,M,R	Q71U36	Polyclonal
Tubulin beta	H,M,R	Q13509	Polyclonal
Tubulin gamma	H,M,R	P23258	Polyclonal
TUFM	H,M,R	P49411	Polyclonal
TUSC2	H,M	O75896	Polyclonal
TUSC3	H,M,R	Q13454	Polyclonal
TUSC5	H	Q8IXB3	Polyclonal
Tyk2	H	P29597	Monoclonal
TYRO3	H	Q06418	Monoclonal
Tyrosinase	H	P14679	Polyclonal
UBA2	H	Q9UBT2	Polyclonal
UBA5	H,M,R	Q9GZZ9	Polyclonal
UBAC1	H,M,R	Q9BSL1	Polyclonal
UBAP2L	H,M	Q14157	Polyclonal
UBE1L	H	P41226	Polyclonal
UBE3B	H	Q7Z3V4	Polyclonal
UBFD1	H,M,R	O14562	Polyclonal
Ubiquitin	H,M,R	P62988	Polyclonal
UBR1	H,M	Q8IWV7	Polyclonal
UBTD1	H,M,R	Q9HAC8	Polyclonal
ULK3	H,M	Q6PHR2	Polyclonal
UNG	H,M,R	P13051	Polyclonal
UPF1	H,M	Q92900	Polyclonal
URB1	H	O60287	Polyclonal
Urocortin	H,M,R	P55089	Polyclonal
USF2	H,M,R	Q15853	Polyclonal
USP13	H,M	Q92995	Polyclonal
USP19	H,M,R	O94966	Polyclonal
USP24	H,M	Q9UPU5	Polyclonal
USP30	H,M	Q70CQ3	Polyclonal

USP32	H,M	Q8NFA0	Polyclonal
USP36	H	Q9P275	Polyclonal
USP42	H,M	Q9H9J4	Polyclonal
USP53	H	Q70EK8	Polyclonal
USP6NL	H	Q92738	Polyclonal
VANGL1	H,M	Q8TAA9	Polyclonal
VCAM1	H	P19320	Monoclonal
VEGFB	H,M,R	P49765	Polyclonal
Vimentin	H,M,R	P08670	Polyclonal
WASF3	H,M	Q9UPY6	Polyclonal
WASF4	H	Q8IV90	Polyclonal
WDHD1	H	O75717	Polyclonal
WEE2	H	P0C1S8	Polyclonal
WNT 10B	H	O00744	Monoclonal
WNT1	H,M	P04628	Polyclonal
WNT5A	H	P41221	Monoclonal
XPA	H,M	P23025	Polyclonal
XPF	H,M	Q92889	Polyclonal
XRCC1	H,M,R	P18887	Polyclonal
XRCC2	H	O43543	Polyclonal
XRCC3	H	O43542	Polyclonal
XRCC3	H	O43542	Polyclonal
XRCC4	H	Q13426	Polyclonal
XRCC5	H,M	P13010	Polyclonal
XRCC6	H,M	P12956	Polyclonal
YAP	H,M,R	P46937	Polyclonal
YES1	H	P07947	Monoclonal
ZADH1	H,M,R	Q8N8N7	Polyclonal
ZADH2	H,M	Q8N4Q0	Polyclonal
ZAP70	H	P43403	Monoclonal
ZHX2	H,M	Q9Y6X8	Polyclonal
ZP1	H	P60852	Polyclonal
ZP4	H,R	Q12836	Polyclonal
Empty Spot			
Negative Control			
Positive Marker			

Abbreviations: H, Human; M, Mouse; R, Rat.

Supplementary Table 3. FC1.8_differential protein statistics between groups of antibody-array

Name	Gene Symbol	Swiss Prot	Huh7_vector_ Normalization	Huh7_2082_OE_Normalization	FC>=1.8_ Huh7_2082_OE_vs_Huh7_vector_
CA125	MUC16	Q8WXI7	270.91	8849.60	32.67
FGF-2	FGF2	P09038	121.00	1591.99	13.16
FGF-1	FGF1	P05230	134.51	1660.31	12.34
IL-1 alpha	IL1A	P01583	138.07	1537.57	11.14
EGFR	EGFR	P00533	155.06	1434.68	9.25
Angiopoietin-2	ANGPT2	O15123	191.29	1167.40	6.10
alpha hCG	CGA	P01215	122.53	550.59	4.49
Angiopoietin-1	ANGPT1	Q15389	278.67	1154.01	4.14
PTK6 (breast tumor kinase)	PTK6	Q13882	112.57	417.24	3.71
Collagen XXV alpha1	COL25A1	Q9BXS0	128.37	433.68	3.38
GPR175	TPRA1	Q86W33	130.44	422.84	3.24
SENP3	SENP3	Q9H4L4	141.29	444.78	3.15
Thioredoxin (TRX)	TXN	P10599	117.60	365.76	3.11
Collagen XVIII alpha1	COL18A1	P39060	137.17	410.75	2.99
Sirp alpha1	SIRPA	P78324	169.32	503.33	2.97
JAK3	JAK3	P52333	114.97	340.61	2.96
GPR132	GPR132	Q9UNW8	143.03	419.35	2.93
Total PSA	KLK3	P07288	123.46	359.29	2.91
Survivin	BIRC5	O15392	183.49	530.26	2.89
PSA-ACT	KLK3	P07288	122.97	349.47	2.84
CRP	CRP	P02741	142.52	404.38	2.84
IL-10	IL10	P22301	161.53	454.66	2.81
AMACR	AMACR	Q9UHK6	143.73	402.27	2.80
TGF beta1	TGFB1	P01137	191.01	522.22	2.73
IgE	N/A	N/A	152.32	410.60	2.70
PTEN	PTEN	P60484	148.45	399.57	2.69
IgG	N/A	N/A	122.97	322.66	2.62
SUMO2/3 (Cleaved-Gly93)	SUMO2	P61956	180.47	472.51	2.62

cTnI (TNNI3)	TNNI3	P19429	188.68	493.31	2.61
CD34	CD34	P28906	123.60	323.11	2.61
CER1	CER1	O95813	126.00	328.04	2.60
HPRT	HPRT1	P00492	142.19	369.41	2.60
STEA3	STEAP3	Q658P3	172.49	443.04	2.57
RSK1 (p90 RSK)	RPS6KA1	Q15418	125.97	322.39	2.56
CEA	PSG2	P11465	118.04	301.18	2.55
Collagen XIX alpha1	COL19A1	Q14993	131.32	331.16	2.52
ITGA5	ITGA5	P08648	131.49	331.30	2.52
GPR153	GPR153	Q6NV75	204.20	513.15	2.51
Lys-acetylated proteins	N/A	N/A	161.37	402.41	2.49
BLK	BLK	P51451	140.12	349.14	2.49
HCK	HCK	P08631	139.63	346.51	2.48
Glucose-6-phosphate isomerase	GPI	P06744	127.14	312.64	2.46
EGF	EGF	P01133	136.47	334.75	2.45
hCG	CGA	P01215	126.98	309.71	2.44
GABRA6	GABRA6	Q16445	138.10	335.38	2.43
ACTN alpha-2/3	ACTN2/ACTN3	P35609/Q08043	146.03	351.66	2.41
RAN	RAN	P62826	180.84	434.83	2.40
STYK1	STYK1	Q6J9G0	169.56	407.67	2.40
ZAP70	ZAP70	P43403	124.95	300.12	2.40
CIB1	CIB1	Q99828	137.03	328.67	2.40
GPR174	GPR174	Q9BXC1	141.38	336.76	2.38
SNAI2 (SLUG)	SNAI2	O43623	180.40	426.75	2.37
Cyclin L1	CCNL1	Q9UK58	116.97	275.88	2.36
ERAS	ERAS	Q7Z444	188.73	442.52	2.34
FADD	FADD	Q13158	174.02	404.97	2.33
Free PSA (KLK3)	KLK3	P07288	119.51	277.25	2.32
ROR1	ROR1	Q01973	127.56	295.07	2.31
MAP2K2 (MEK2)	MAP2K2	P36507	228.70	528.80	2.31
EMR1	ADGRE1	Q14246	126.49	291.57	2.31
PROZ	PROZ	P22891	170.60	392.86	2.30
JAK2	JAK2	O60674	148.48	340.20	2.29
FAK	PTK2	Q05397	174.37	398.64	2.29

Smad4	SMAD4	Q13485	118.62	270.79	2.28
ULK3	ULK3	Q6PHR2	149.50	340.96	2.28
Dipeptidyl-peptidase 1 (heavy chain,Cleaved-Arg 394)	CTSC	P53634	153.85	350.80	2.28
PIGH	PIGH	Q14442	126.56	287.67	2.27
Influenza B virus Nucleoprotein	NP	P04665	151.62	344.20	2.27
DAXX	DAXX	Q9UER7	152.45	344.89	2.26
SLC4A11	SLC4A11	Q8NBS3	177.42	400.06	2.25
HGH	GH1	P01241	137.63	310.12	2.25
WNT 10B	WNT10B	O00744	136.52	306.44	2.24
S100B	S100B	P04271	138.54	310.25	2.24
Cytochrome P450 1A1/2	CYP1A1	P04798	138.80	310.38	2.24
CD33	CD33	P20138	131.98	294.90	2.23
XRCC3	XRCC3	O43542	139.29	310.48	2.23
Retinoid X Receptor gamma	RXRG	P48443	211.99	472.23	2.23
IL-6	IL6	P05231	134.96	298.40	2.21
GPR152	GPR152	Q8TDT2	115.48	255.20	2.21
CYB5R3	CYB5R3	P00387	133.54	294.63	2.21
PDRG1	PDRG1	Q9NUG6	141.49	311.72	2.20
CSF2 (GM-CSF)	CSF2	P04141	152.59	334.86	2.19
Collagen XII alpha1	COL12A1	Q99715	146.89	321.54	2.19
GPR173	GPR173	Q9NS66	161.95	352.22	2.17
NKX3A	NKX3-1	Q99801	175.44	380.94	2.17
Ferritin	FTH1	P02794	159.55	346.24	2.17
Calcyclin (S100A6)	S100A6	P06703	175.95	379.65	2.16
Akt3	AKT3	Q9Y243	115.02	247.93	2.16
SH-PTP2	PTPN11	Q06124	259.14	556.29	2.15
CD18 (ITGB2)	ITGB2	P05107	139.47	299.25	2.15
GPR171	GPR171	O14626	135.45	290.55	2.15
TSH	TSHB	P01222	144.03	308.24	2.14
PDGFR beta	PDGFRB	P09619	147.43	315.43	2.14

HLAH	HLA-H	P01893	131.05	278.64	2.13
KCNJ9	KCNJ9	Q92806	174.84	371.33	2.12
DNAL1	DNAL1	Q4LDG9	134.09	284.36	2.12
GFR alpha-1	GFRA1	P56159	136.72	289.40	2.12
beta hCG	N/A	P01233	149.96	317.19	2.12
ALCAM	ALCAM	Q13740	167.58	354.33	2.11
Foxp3	FOXP3	B7ZLG1	151.08	318.78	2.11
IGF 1R	IGF1R	P08069	151.55	319.66	2.11
FABP4	FABP4	P15090	170.44	359.28	2.11
CaMK2beta/gamma	CAMK2B/CAMK2G	Q13554/Q13555	146.82	309.28	2.11
PARP	PARP1	P09874	163.65	343.86	2.10
FRS3	FRS3	O43559	145.85	306.12	2.10
PARK7	PARK7	Q99497	129.09	270.82	2.10
MATK (CTK)	MATK	P42679	138.63	290.20	2.09
SRA	SRA1	Q9HD15	145.05	303.54	2.09
ADAM 17 (Cleaved-Arg215)	ADAM17	P78536	160.64	335.37	2.09
EPCAM	EPCAM	P16422	158.99	331.16	2.08
CD44	CD44	P16070	154.20	320.80	2.08
DDR1	DDR1	Q08345	174.07	361.70	2.08
NKX26	NKX2-6	A6NCS4	150.83	313.18	2.08
human Albumin	ALB	P02768	161.53	334.29	2.07
RON	MST1R	Q04912	157.99	326.86	2.07
SIX5	SIX5	Q8N196	121.93	252.25	2.07
ITK (LYK)	ITK	Q08881	156.15	322.47	2.07
CA181	ZNHIT6	Q9NWK9	169.93	350.92	2.07
Collagen III alpha1 (Cleaved-Gly1221)	COL3A1	P02461	150.90	311.23	2.06
Prolactin	PRL	P01236	161.11	330.61	2.05
CA19-9	ST6GALNAC6	Q969X2	197.83	404.94	2.05
Rab25	esr1	P57753	145.61	297.77	2.04
LDLRAD2	LDLRAD2	Q5SZI1	130.01	265.36	2.04
MARK3	MARK3	P27448	141.49	288.76	2.04
CD45	PTPRC	P08575	134.05	273.35	2.04
SORL1	SORL1	Q92673	165.02	336.46	2.04
WNT5A	WNT5A	P41221	134.00	273.10	2.04
CDKL2	CDKL2	Q92772	167.00	340.25	2.04

EMR2	ADGRE2	Q9UHX3	114.55	233.08	2.03
ACSL6	ACSL6	Q9UKU0	130.01	264.29	2.03
ALDOB	ALDOB	P05062	154.15	313.04	2.03
BMX (ETK)	BMX	P51813	144.50	292.66	2.03
HOXA6	HOXA6	P31267	141.94	287.32	2.02
S100A10/ P11	S100A10	P60903	123.53	249.54	2.02
VCAM1	VCAM1	P19320	1952.69	3940.55	2.02
FOXA2	FOXA2	Q9Y261	166.95	336.78	2.02
ME1	ME1	P48163	128.53	258.84	2.01
SAR1B	SAR1B	Q9Y6B6	140.07	281.51	2.01
IgM	N/A	N/A	182.57	366.72	2.01
Connexin 43	GJA1	P17302	144.40	289.95	2.01
URB1	URB1	O60287	124.09	248.54	2.00
ARF4	ARF4	P18085	131.03	262.21	2.00
ITGA6 (light chain,Cleaved-Glu 942)	ITGA6	P23229	127.56	254.51	2.00
CD10	MME	P08473	120.53	240.34	1.99
THRB (AP2,Cleaved-Arg 327)	F2	P00734	168.09	334.84	1.99
ATRX	ATRX	P46100	138.91	276.12	1.99
AASDHPPT	AASDHPPT	Q9NRN7	144.87	287.94	1.99
ELOVL4	ELOVL4	Q9GZR5	124.42	246.91	1.98
AARSD1	AARSD1	Q9BTE6	146.57	290.75	1.98
MER	MERTK	Q12866	168.53	334.32	1.98
GPR160	GPR160	Q9UJ42	134.93	267.15	1.98
Neurogenin-3	NEUROG3	Q9Y4Z2	122.55	242.02	1.97
ACTL6A	ACTL6A	O96019	137.45	270.65	1.97
ATP2C1	ATP2C1	P98194	150.03	295.28	1.97
IGLL1	IGLL1	P15814	129.47	254.35	1.96
CD97beta (Cleaved-Ser531)	CD97	P48960	165.16	323.51	1.96
USP53	USP53	Q70EK8	150.48	294.49	1.96
SOX2	SOX2	P48431	128.53	250.37	1.95
MUTYH	MUTYH	Q9UIF7	193.22	376.33	1.95
CDC2	CDK1	P06493	200.85	389.45	1.94
CASP4	CASP4	P49662	113.04	219.03	1.94

(p20,Cleaved-Gln81)					
TUSC2	TUSC2	O75896	162.16	314.01	1.94
MLL	KMT2A	Q03164	153.66	297.03	1.93
Tyk2	TYK2	P29597	168.72	325.92	1.93
NT	NTM	Q9P121	131.14	252.98	1.93
RHG22	ARHGAP22	Q7Z5H3	134.12	258.58	1.93
MUM1	MUM1	Q2TAK8	222.45	428.84	1.93
MYST1	KAT8	Q9H7Z6	142.47	274.57	1.93
IKK Alpha	CHUK	O15111	160.11	307.64	1.92
CIDEB	CIDEB	Q9UHD4	137.21	263.47	1.92
ATP5G2	ATP5G2	Q06055	149.61	287.11	1.92
DCT	DCT	P40126	141.49	271.39	1.92
MAST3	MAST3	O60307	170.23	326.08	1.92
WDHD1	WDHD1	O75717	138.07	264.40	1.91
UBE3B	UBE3B	Q7Z3V4	132.61	253.79	1.91
Histone H3 (Acetyl-Lys18)	HIST1H3A-J	P68431	176.70	337.88	1.91
IKBKE (IKK epsilon)	IKBKE	Q14164	143.68	274.30	1.91
SERPINB7	SERPINB7	O75635	211.46	403.22	1.91
DDX4	DDX4	Q9NQI0	133.49	254.53	1.91
MCM5	MCM5	P33992	155.97	297.34	1.91
NCOA7	NCOA7	Q8NI08	124.53	237.37	1.91
GATA3	GATA3	P23771	138.05	263.03	1.91
C1R (light chain,Cleaved-Ile464)	C1R	P00736	186.59	355.34	1.90
Cytochrome P450 39A1	CYP39A1	Q9NYL5	162.71	309.60	1.90
IP6K2	IP6K2	Q9UHH9	139.86	265.20	1.90
NOX3	NOX3	Q9HBY0	135.21	256.05	1.89
6-Phosphofructo-2-Kinase	PFKFB2	O60825	170.79	323.14	1.89
MUSK	MUSK	O15146	144.96	273.93	1.89
S100 A1	S100A1	P23297	142.54	268.75	1.89
TNK1	TNK1	Q13470	182.59	344.04	1.88
Septin-1	SEPT1	Q8WYJ6	181.70	342.21	1.88

PRKX	PRKX	P51817	151.36	284.90	1.88
Fibronectin	FN1	P02751	188.31	353.67	1.88
AIFM2	AIFM2	Q9BRQ8	124.60	233.62	1.87
RED	IK	Q13123	131.05	245.51	1.87
ABHD14A	ABHD14A	Q9BUJ0	131.95	247.14	1.87
Osteopontin	SPP1	P10451	156.58	292.88	1.87
ABHD12	ABHD12	Q8N2K0	148.85	277.72	1.87
MMP1 (Cleaved-Phe100)	MMP1	P03956	127.49	237.29	1.86
SLC28A2	SLC28A2	O43868	134.54	250.27	1.86
AIG1	AIG1	Q9NVV5	110.11	204.43	1.86
IL-2	IL2	P60568	157.57	291.89	1.85
LPL	LPL	P06858	193.01	357.52	1.85
MAP4K4	MAP4K4	O95819	161.53	299.16	1.85
MGMT	MGMT	P16455	185.78	343.61	1.85
RASSF6	RASSF6	Q6ZTQ3	147.40	272.09	1.85
PARP (Cleaved-Gly215)	PARP1	P09874	179.14	330.06	1.84
NOX5	NOX5	Q96PH1	149.08	273.99	1.84
GPRIN1	GPRIN1	Q7Z2K8	163.69	300.56	1.84
POTE8	POTEA	Q6S8J7	135.40	248.15	1.83
DDR2	DDR2	Q16832	156.99	287.62	1.83
TUFM	TUFM	P49411	129.05	236.29	1.83
ADA2L	TADA2A	O75478	135.42	247.97	1.83
Caspase 6 (Cleaved-Asp162)	CASP6	P55212	163.97	300.15	1.83
MYO1D	MYO1D	O94832	131.56	240.47	1.83
UBA5	UBA5	Q9GZZ9	146.17	266.68	1.82
INHA (Inhibin alpha)	INHA	P05111	153.15	279.21	1.82
MYC	MYC	P01106	130.02	236.27	1.82
Mar V	MARCH5	Q9NX47	142.03	258.04	1.82
Mar IV	MARCH4	Q9P2E8	131.05	238.08	1.82
UBAP2L	UBAP2L	Q14157	120.11	217.25	1.81
p50 Dynamitin	DCTN2	Q13561	127.56	230.63	1.81
GSK3 alpha	GSK3A	P49840	151.50	273.76	1.81
GADD45GIP1	GADD45GIP1	Q8TAE8	195.15	351.98	1.80
EPN2	EPN2	O95208	138.49	249.64	1.80

TNF Receptor II	TNFRSF1B	P20333	494.99	269.61	0.54
ORAV1	ORAOV1	Q8WV07	522.80	280.45	0.54
MMP-10	MMP10	P09238	688.51	367.64	0.53
beta-2-Microglobulin	B2M	P61769	1093.77	566.43	0.52
DP-1	TFDP1	Q14186	490.80	253.94	0.52
Claudin 3	CLDN3	O15551	484.35	249.22	0.51
Pax-5	PAX5	Q02548	394.10	199.32	0.51
MYLIP	MYLIP	Q8WY64	504.53	250.78	0.50
FGF22	FGF22	Q9HCT0	573.63	282.83	0.49
Actin-pan	ACTB	P60709	658.89	319.11	0.48
EMR3	ADGRE3	Q9BY15	1342.10	622.98	0.46
ABCD1	ABCD1	P33897	1538.75	678.16	0.44
Caveolin-1	CAV1	Q03135	607.73	266.37	0.44
ACTR-1C	ACVR1C	Q8NER5	607.12	264.68	0.44
GAD1	GAD1	Q99259	589.75	252.07	0.43
HER3	ERBB3	P21860	768.90	325.14	0.42
Patched	PTCH1	Q13635	714.95	299.71	0.42
GLUT1	SLC2A1	P11166	753.03	312.83	0.42
CMC1	SLC25A12	O75746	692.53	264.47	0.38
CDH9	CDH9	Q9ULB4	949.41	357.81	0.38
SERPINB9	SERPINB9	P50453	1282.23	454.62	0.35
GRB14	GRB14	Q14449	2132.82	733.69	0.34
mGluR2/3	GRM2	Q14416	851.76	292.01	0.34
GAD1/2	GAD1	Q99259	807.34	270.36	0.33
ARC	NOL3	O60936	1154.69	371.78	0.32
ARFIP1	ARFIP1	P53367	1866.78	595.11	0.32
Keratin 18	KRT18	P05783	1234.78	388.95	0.31
SOD1	SOD1	P00441	1261.87	391.75	0.31
CDH24	CDH24	Q86UP0	1685.41	521.55	0.31
GPR151	GPR151	Q8TDV0	1342.23	403.85	0.30
Cytochrome b561 D1	CYB561D1	Q8N8Q1	3791.27	1133.84	0.30
Cullin 1	CUL1	Q13616	947.42	282.56	0.30
Claudin 5	CLDN5	O00501	1406.43	378.62	0.27
ARSI	ARSI	Q5FYB1	1436.04	366.65	0.26
KCNMB2	KCNMB2	Q9Y691	1293.46	320.82	0.25
p42 MAPK	MAPK1	P28482	1217.19	295.08	0.24

Caspase 7 (Cleaved-Asp198)	CASP7	P55210	1049.01	243.19	0.23
Desmin	DES	P17661	1889.41	394.44	0.21
Actin-alpha-1	ACTA1	P68133	1553.21	308.91	0.20
Peripherin	PRPH	P41219	1438.72	267.06	0.19
Testosterone	SHBG	P04278	1767.12	320.18	0.18
Tubulin alpha	TUBA1A	Q71U36	1839.69	262.29	0.14
HSP90A	HSP90AA1	P07900	1910.45	258.87	0.14

Supplementary Table 4. GO enrichment analysis of up-regulated proteins in antibody-array

Category	Term	Count	Genes	Category	Term
GOTERM_BP_DIRECT	GO:0007165~signal transduction	40	CGA, S100A6, SORL1, GJA1, DDR2, ALCAM, ARHGAP22, IGF1R, GATA3, FRS3, ACTL6A, EGF, FGF1, ANGPT2, FGF2, AKT3, EGFR, GPR173, ITK,	GOTERM_BP_DIRECT	GO:0007166~signal transduction

			RAN, GABRA6, BMX, ESR1, FADD, INHA, CDKL2, RASSF6, RPS6KA1, CD34, ATP2C1, CD33, TXN, PDGFRB, JAK2, MST1R		
GOTERM_BP_DIRECT	GO:0008284~positive regulation of cell proliferation	30	CSF2, CGA, PTEN, TGFB1, EPCAM, IGF1R, PTK2, GADD45GIP1, NKX3-1, FGF1, EGF, MYC, FGF2, MATK, CIB1, FN1, EGFR, COL18A1, IL6, WNT10B, NKX2-6, HCK, BIRC5, S100B, F2, ADAM17, PDGFRB, MST1R, IL2	GOTERM_BP_DIRECT	GO:0008285~positive regulation of cell proliferation
GOTERM_BP_DIRECT	GO:0007155~cell adhesion	25	ITGB2, DDR2, PRKX, VCAM1, ALCAM, CD44, COL12A1, CIB1, FN1, SPP1, COL18A1,	GOTERM_BP_DIRECT	GO:0007156~cell adhesion

			ADGRE1, ADGRE2, HCK, SIRPA, DDR1, COL19A1, ITGA6, CD34, ITGA5, CD33, ADAM17, NTM, IL2, MUC16		
GOTERM_BP_DIRECT	GO:0000165~MAPK cascade	21	EGFR, CSF2,MAP2K2, MARK3, TGFB1, PTK2, GFRA1, PDGFRB, FRS3, JAK2, ANGPT1, JAK3, FGF1, EGF, FGF2, MYC, IL2	GOTERM_BP_DIRECT	GO:0000166~MAPK cascade
GOTERM_BP_DIRECT	GO:0008283~cell proliferation	19	TUSC2, EGFR, CDK1, NOX5, CYP1A1, SRA1, SMAD4, PTEN, DCTN2, S100B, CD34, TXN, FGF1, PRL, MYC, IL1A, MATK	GOTERM_BP_DIRECT	GO:0008284~cell proliferation
GOTERM_BP_DIRECT	GO:0030335~positive regulation of cell migration	15	COL18A1, EGFR, CGA, SNAI2, TGFB1, IGF1R, PTK2, ITGA6, ITGA5,	GOTERM_BP_DIRECT	GO:0030336~positive regulation of cell migration

			PDGFRB, ADAM17, JAK2, FGF1, CIB1		
GOTERM_BP_DIRECT	GO:0016477~cell migration	13	TYK2, CDK1, STYK1, ADGRE2, PTK6, ARF4, PSG2, PDGFRB, JAK2, JAK3, PTEN, TGFB1, MATK	GOTERM_BP_DIRECT	GO:0016478~cell migration
GOTERM_BP_DIRECT	GO:0042127~regulation of cell proliferation	13	ITK, BLK, BMX, INHA, TYK2, PTK2, STYK1, PTK6, TNK1, JAK2, CIB1	GOTERM_BP_DIRECT	GO:0042128~regulation of cell proliferation
GOTERM_BP_DIRECT	GO:0071356~cellular response to tumor necrosis factor	7	VCAM1, IL6, GATA3, NKX3-1, FABP4, CHUK, CIB1	GOTERM_BP_DIRECT	GO:0071357~cellular response to tumor necrosis factor
GOTERM_BP_DIRECT	GO:0030307~positive regulation of cell growth	6	EGFR, RPS6KA1, F2, ADAM17, CIB1, IL2	GOTERM_BP_DIRECT	GO:0030308~positive regulation of cell growth
GOTERM_BP_DIRECT	GO:0000186~activation of MAPKK activity	4	EGFR, JAK2, EGF, FGF2	GOTERM_BP_DIRECT	GO:0000187~activation of MAPKK activity
GOTERM_BP_DIRECT	GO:0060396~growth hormone receptor signaling pathway	3	GH1, PTK2, JAK2	GOTERM_BP_DIRECT	GO:0060397~growth hormone receptor signaling pathway

GOTERM_BP_DIRECT	GO:0022408~negative regulation of cell-cell adhesion	3	PTK2, JAK2, TGFB1	GOTERM_BP_DIRECT	GO:0022409~negative regulation of cell-cell adhesion
GOTERM_BP_DIRECT	GO:0048864~stem cell development	2	PTPRC, FGF2	GOTERM_BP_DIRECT	GO:0048865~stem cell development

Supplementary Table 5. 10 down-regulated proteins involved in tumor suppression in antibody-array

No.	Name	Gene Symbol	Swiss Prot	Huh7_vector_ Normalization	Huh7_2082_OE_ Normalization	Huh7_2082_OE_vs_Huh7_vector_
1	Claudin 3	CLDN3	O15551	484.35	249.22	0.51
2	Pax-5	PAX5	Q02548	394.10	199.32	0.51
3	MYLIP	MYLIP	Q8WY64	504.53	250.78	0.50
4	ACTR-1C	ACVR1C	Q8NER5	607.12	264.68	0.44
5	ARFIP1	ARFIP1	P53367	1866.78	595.11	0.32
6	Keratin 18	KRT18	P05783	1234.78	388.95	0.31
7	CDH24	CDH24	Q86UP0	1685.41	521.55	0.31
8	Claudin 5	CLDN5	O00501	1406.43	378.62	0.27
9	Caspase 7 (Cleaved-Asp198)	CASP7	P55210	1049.01	243.19	0.23
10	Actin-alpha-1	ACTA1	P68133	1553.21	308.91	0.20

Supplementary Table 6. 322 potential miRNAs binding with circ-MALAT1

No.	miRNA	Total Score	Total Energy	Max Score	Max Energy	Len1	Len2	Positions
1	hsa-miR-6756-3p	304	-65.41	158	-35.68	20	867	542 350
2	hsa-miR-4640-3p	313	-64.23	162	-34.5	22	867	542 349
3	hsa-miR-512-5p	459	-60.82	162	-27.21	23	867	772 823 757
4	hsa-miR-6887-3p	323	-59.24	162	-30.5	21	867	351 538
5	hsa-miR-6892-3p	288	-58.44	148	-30.64	21	867	351 538
6	hsa-miR-4773	457	-53.42	159	-17.98	22	867	458 622 648
7	hsa-miR-4742-3p	423	-52.56	142	-18.93	23	867	595 642 120
8	hsa-miR-1224-3p	301	-52.27	158	-38.28	21	867	544 164
9	hsa-miR-6865-3p	293	-50.7	147	-26.28	21	867	350 543
10	hsa-miR-4323	302	-48.25	159	-26.05	18	867	551 367
11	hsa-miR-214-3p	285	-45.74	145	-27.71	22	867	628 453
12	hsa-miR-6767-5p	304	-44.8	152	-23.11	23	867	1 860
13	hsa-miR-4713-5p	289	-43.27	146	-26.04	22	867	165 532
14	hsa-miR-1260a	287	-41.55	147	-25.99	18	867	167 549
15	hsa-miR-676-5p	296	-38.44	153	-22.23	21	867	739 779
16	hsa-miR-3611	445	-38.37	157	-14.39	21	867	80 581 256
17	hsa-miR-483-3p	145	-37.75	145	-37.75	21	867	544
18	hsa-miR-6807-3p	316	-37.08	160	-19.1	23	867	643 594

19	hsa-miR-503-5p	172	-36.46	172	-36.46	23	867	656
20	hsa-miR-4298	152	-36.41	152	-36.41	22	867	458
21	hsa-miR-1260b	281	-36.2	141	-22.31	19	867	548 166
22	hsa-miR-3166	296	-35.96	149	-17.98	23	867	860 1
23	hsa-miR-6862-3p	141	-35.95	141	-35.95	24	867	541
24	hsa-miR-4433a-5p	159	-35.72	159	-35.72	21	867	547
25	hsa-miR-6779-3p	142	-35.58	142	-35.58	21	867	545
26	hsa-miR-519d-5p	294	-35.02	150	-18.25	25	867	517 381
27	hsa-miR-1299	297	-34.85	153	-18.14	22	867	204 105
28	hsa-miR-3529-3p	297	-34.81	152	-17.62	24	867	553 737
29	hsa-miR-4667-3p	168	-34.75	168	-34.75	21	867	539
30	hsa-miR-6845-3p	145	-34.09	145	-34.09	21	867	540
31	hsa-miR-6752-3p	141	-34.02	141	-34.02	21	867	547
32	hsa-miR-605-5p	160	-33.77	160	-33.77	23	867	362
33	hsa-miR-5196-3p	142	-33.6	142	-33.6	21	867	542
34	hsa-miR-1205	282	-33.33	142	-17.69	20	867	792 482
35	hsa-miR-3158-5p	287	-33.12	146	-17.5	21	867	791 386
36	hsa-miR-3680-5p	300	-32.75	150	-17.29	22	867	462 485
37	hsa-miR-1324	294	-32.74	151	-18.12	24	867	617 852
38	hsa-miR-3162-3p	153	-32.74	153	-32.74	21	867	547
39	hsa-miR-1236-3p	144	-32.58	144	-32.58	22	867	539
40	hsa-miR-5088-3p	140	-31.98	140	-31.98	21	867	541
41	hsa-miR-877-3p	150	-31.83	150	-31.83	21	867	538
42	hsa-miR-6787-3p	287	-31.39	145	-17.16	22	867	756 823
43	hsa-miR-6826-3p	162	-31.38	162	-31.38	22	867	350
44	hsa-miR-4290	149	-30.7	149	-30.7	19	867	542
45	hsa-miR-7114-3p	141	-30.57	141	-30.57	21	867	548
46	hsa-miR-6729-3p	146	-30.56	146	-30.56	21	867	542
47	hsa-miR-3686	323	-30.32	175	-15.47	22	867	578 385
48	hsa-miR-548at-5p	315	-30.1	158	-17.45	22	867	278 47
49	hsa-miR-4705	298	-30.05	153	-17.99	22	867	814 733
50	hsa-miR-6870-3p	144	-29.98	144	-29.98	22	867	544
51	hsa-miR-4758-3p	162	-29.64	162	-29.64	23	867	547
52	hsa-miR-1225-3p	140	-29.18	140	-29.18	22	867	548
53	hsa-miR-6744-3p	171	-29.01	171	-29.01	23	867	124
54	hsa-miR-3184-3p	141	-28.94	141	-28.94	23	867	547
55	hsa-miR-7109-3p	152	-28.76	152	-28.76	22	867	548

56	hsa-miR-34b-3p	301	-28.73	161	-22.3	22	867	733 237
57	hsa-miR-7108-3p	140	-28.54	140	-28.54	20	867	545
58	hsa-miR-4699-3p	296	-28.31	150	-20.21	22	867	638 178
59	hsa-miR-6784-3p	144	-28.12	144	-28.12	22	867	546
60	hsa-miR-4749-3p	141	-27.8	141	-27.8	20	867	540
61	hsa-miR-6879-3p	143	-27.7	143	-27.7	21	867	355
62	hsa-miR-5193	154	-27.15	154	-27.15	22	867	538
63	hsa-miR-589-3p	289	-27.01	145	-13.65	24	867	648 622
64	hsa-miR-4436a	155	-26.89	155	-26.89	21	867	625
65	hsa-miR-7111-3p	156	-26.65	156	-26.65	22	867	535
66	hsa-miR-3661	145	-26.12	145	-26.12	22	867	613
67	hsa-miR-6749-3p	154	-25.96	154	-25.96	21	867	536
68	hsa-miR-6798-3p	140	-25.84	140	-25.84	21	867	545
69	hsa-miR-203a-3p	287	-25.73	147	-13.61	22	867	394 198
70	hsa-miR-4468	156	-25.19	156	-25.19	18	867	460
71	hsa-miR-4757-5p	150	-25.02	150	-25.02	23	867	122
72	hsa-miR-4633-3p	140	-24.82	140	-24.82	22	867	445
73	hsa-miR-20a-3p	284	-24.73	142	-12.47	22	867	595 638
74	hsa-miR-6802-3p	141	-24.71	141	-24.71	22	867	543
75	hsa-miR-4269	144	-24.55	144	-24.55	21	867	358
76	hsa-miR-3127-3p	140	-24.49	140	-24.49	22	867	539
77	hsa-miR-2116-3p	150	-24.23	150	-24.23	21	867	534
78	hsa-miR-8075	140	-23.82	140	-23.82	24	867	489
79	hsa-miR-885-3p	142	-23.77	142	-23.77	22	867	661
80	hsa-miR-217	280	-23.73	140	-11.9	23	867	595 641
81	hsa-miR-3192-3p	152	-23.71	152	-23.71	21	867	177
82	hsa-miR-4685-3p	151	-23.49	151	-23.49	22	867	346
83	hsa-miR-6511a-5p	141	-23.46	141	-23.46	23	867	617
84	hsa-miR-6789-3p	141	-23.41	141	-23.41	21	867	356
85	hsa-miR-3154	142	-23.39	142	-23.39	22	867	451
86	hsa-miR-668-3p	143	-23.06	143	-23.06	23	867	759
87	hsa-miR-1182	141	-23.05	141	-23.05	23	867	125
88	hsa-miR-518a-5p	166	-23.01	166	-23.01	20	867	385
89	hsa-miR-527	166	-23.01	166	-23.01	20	867	385
90	hsa-miR-3667-3p	142	-22.84	142	-22.84	22	867	539
91	hsa-miR-187-5p	158	-22.62	158	-22.62	22	867	558
92	hsa-miR-5582-3p	284	-22.4	143	-11.66	22	867	267 507

93	hsa-miR-670-3p	156	-22.32	156	-22.32	21	867	780
94	hsa-miR-4514	140	-22.18	140	-22.18	18	867	623
95	hsa-miR-508-5p	144	-21.84	144	-21.84	23	867	691
96	hsa-miR-4530	143	-21.82	143	-21.82	18	867	631
97	hsa-miR-125b-2-3p	143	-21.75	143	-21.75	22	867	810
98	hsa-miR-3129-5p	157	-21.74	157	-21.74	22	867	325
99	hsa-miR-223-3p	141	-21.74	141	-21.74	22	867	231
100	hsa-miR-7113-5p	141	-21.71	141	-21.71	21	867	517
101	hsa-miR-6125	142	-21.64	142	-21.64	20	867	455
102	hsa-miR-1304-3p	158	-21.51	158	-21.51	22	867	482
103	hsa-miR-6894-3p	140	-21.5	140	-21.5	21	867	310
104	hsa-miR-7152-3p	148	-21.44	148	-21.44	20	867	17
105	hsa-miR-6876-5p	146	-21.42	146	-21.42	22	867	450
106	hsa-miR-6895-5p	145	-21.32	145	-21.32	21	867	446
107	hsa-miR-639	144	-21.25	144	-21.25	23	867	480
108	hsa-miR-8063	152	-20.99	152	-20.99	22	867	421
109	hsa-miR-337-5p	142	-20.92	142	-20.92	21	867	412
110	hsa-miR-607	156	-20.9	156	-20.9	21	867	28
111	hsa-miR-556-5p	140	-20.9	140	-20.9	22	867	489
112	hsa-miR-6501-3p	147	-20.87	147	-20.87	23	867	649
113	hsa-miR-6894-5p	145	-20.82	145	-20.82	24	867	397
114	hsa-miR-4512	153	-20.81	153	-20.81	22	867	123
115	hsa-miR-4709-5p	144	-20.73	144	-20.73	22	867	737
116	hsa-miR-6771-3p	150	-20.63	150	-20.63	21	867	364
117	hsa-miR-17-3p	140	-20.47	140	-20.47	22	867	610
118	hsa-miR-6731-5p	145	-20.4	145	-20.4	22	867	448
119	hsa-miR-548ar-3p	171	-20.08	171	-20.08	21	867	795
120	hsa-miR-935	140	-20.08	140	-20.08	23	867	41
121	hsa-miR-6734-3p	145	-20.05	145	-20.05	23	867	342
122	hsa-miR-298	140	-20.01	140	-20.01	24	867	449
123	hsa-miR-1283	148	-19.67	148	-19.67	22	867	384
124	hsa-miR-767-3p	142	-19.64	142	-19.64	23	867	31
125	hsa-miR-4778-5p	152	-19.53	152	-19.53	22	867	580
126	hsa-miR-4638-5p	141	-19.5	141	-19.5	21	867	825
127	hsa-miR-6830-3p	154	-19.39	154	-19.39	23	867	61
128	hsa-miR-421	148	-19.31	148	-19.31	23	867	735
129	hsa-miR-548ba	144	-19.27	144	-19.27	22	867	602

130	hsa-miR-492	145	-19.24	145	-19.24	23	867	614
131	hsa-miR-1226-3p	151	-19.23	151	-19.23	22	867	838
132	hsa-miR-1295b-3p	142	-19.08	142	-19.08	22	867	219
133	hsa-miR-3173-3p	145	-18.98	145	-18.98	22	867	450
134	hsa-miR-136-5p	143	-18.87	143	-18.87	23	867	690
135	hsa-miR-6077	149	-18.71	149	-18.71	21	867	447
136	hsa-miR-6760-3p	146	-18.58	146	-18.58	21	867	232
137	hsa-miR-619-3p	149	-18.54	149	-18.54	24	867	104
138	hsa-miR-2110	150	-18.47	150	-18.47	22	867	713
139	hsa-miR-4421	144	-18.33	144	-18.33	22	867	158
140	hsa-miR-6715b-5p	141	-18.31	141	-18.31	21	867	357
141	hsa-miR-4635	147	-18.23	147	-18.23	21	867	612
142	hsa-miR-6839-3p	153	-18.22	153	-18.22	22	867	749
143	hsa-miR-154-3p	150	-18.13	150	-18.13	22	867	695
144	hsa-miR-6757-5p	143	-18.01	143	-18.01	22	867	399
145	hsa-miR-3164	142	-17.82	142	-17.82	22	867	215
146	hsa-miR-550b-2-5p	150	-17.75	150	-17.75	22	867	112
147	hsa-miR-6747-3p	145	-17.69	145	-17.69	21	867	57
148	hsa-miR-3120-3p	141	-17.62	141	-17.62	21	867	836
149	hsa-miR-4728-3p	148	-17.59	148	-17.59	25	867	492
150	hsa-miR-1270	155	-17.57	155	-17.57	23	867	262
151	hsa-miR-572	142	-17.56	142	-17.56	20	867	33
152	hsa-miR-4762-5p	151	-17.49	151	-17.49	21	867	26
153	hsa-miR-101-3p	166	-17.45	166	-17.45	21	867	648
154	hsa-miR-3974	151	-17.35	151	-17.35	23	867	327
155	hsa-miR-6770-5p	143	-17.32	143	-17.32	24	867	450
156	hsa-miR-561-3p	286	-17.31	146	-10.08	22	867	851 1
157	hsa-miR-3659	153	-17.22	153	-17.22	21	867	1
158	hsa-miR-450a-2-3p	147	-17.18	147	-17.18	22	867	712
159	hsa-miR-6846-3p	150	-17.14	150	-17.14	21	867	219
160	hsa-miR-4772-5p	145	-17.05	145	-17.05	22	867	653
161	hsa-miR-2276-3p	153	-17.04	153	-17.04	22	867	479
162	hsa-miR-495-5p	140	-17	140	-17	22	867	47
163	hsa-miR-4776-3p	148	-16.94	148	-16.94	23	867	112
164	hsa-miR-1-3p	162	-16.84	162	-16.84	22	867	260
165	hsa-miR-627-5p	144	-16.81	144	-16.81	22	867	582
166	hsa-miR-1238-3p	142	-16.8	142	-16.8	20	867	782

167	hsa-miR-550a-5p	148	-16.78	148	-16.78	23	867	107
168	hsa-miR-3116	153	-16.73	153	-16.73	22	867	106
169	hsa-miR-4732-3p	144	-16.66	144	-16.66	21	867	219
170	hsa-miR-206	154	-16.57	154	-16.57	22	867	260
171	hsa-miR-4789-5p	144	-16.48	144	-16.48	22	867	842
172	hsa-miR-3202	143	-16.47	143	-16.47	22	867	447
173	hsa-miR-516b-5p	148	-16.46	148	-16.46	22	867	207
174	hsa-miR-33b-3p	147	-16.28	147	-16.28	22	867	391
175	hsa-miR-4645-5p	141	-16.27	141	-16.27	19	867	518
176	hsa-miR-6815-3p	142	-16.15	142	-16.15	21	867	126
177	hsa-miR-1254	145	-16.12	145	-16.12	24	867	105
178	hsa-miR-508-3p	172	-16.11	172	-16.11	23	867	294
179	hsa-miR-148a-5p	143	-16.1	143	-16.1	22	867	374
180	hsa-miR-34c-3p	143	-16.08	143	-16.08	22	867	730
181	hsa-miR-3606-3p	161	-16.01	161	-16.01	21	867	786
182	hsa-miR-383-5p	156	-16.01	156	-16.01	22	867	654
183	hsa-miR-455-5p	151	-15.99	151	-15.99	22	867	112
184	hsa-miR-199a-3p	145	-15.99	145	-15.99	22	867	325
185	hsa-miR-199b-3p	145	-15.99	145	-15.99	22	867	325
186	hsa-miR-548ai	143	-15.99	143	-15.99	22	867	602
187	hsa-miR-570-5p	143	-15.99	143	-15.99	22	867	602
188	hsa-miR-6716-5p	140	-15.94	140	-15.94	20	867	263
189	hsa-miR-7157-5p	140	-15.94	140	-15.94	23	867	639
190	hsa-miR-4659b-5p	151	-15.92	151	-15.92	19	867	117
191	hsa-miR-24-1-5p	142	-15.87	142	-15.87	22	867	309
192	hsa-miR-3124-3p	144	-15.85	144	-15.85	22	867	60
193	hsa-miR-548g-3p	141	-15.82	141	-15.82	22	867	196
194	hsa-miR-301a-5p	152	-15.78	152	-15.78	22	867	216
195	hsa-miR-4755-3p	140	-15.76	140	-15.76	22	867	442
196	hsa-miR-374c-5p	146	-15.74	146	-15.74	22	867	844
197	hsa-miR-1271-3p	144	-15.74	144	-15.74	22	867	109
198	hsa-miR-509-3-5p	150	-15.71	150	-15.71	22	867	792
199	hsa-miR-532-3p	142	-15.71	142	-15.71	22	867	161
200	hsa-miR-6777-3p	140	-15.58	140	-15.58	20	867	306
201	hsa-miR-5000-3p	148	-15.53	148	-15.53	22	867	651
202	hsa-miR-320b	140	-15.51	140	-15.51	22	867	563
203	hsa-miR-487a-3p	140	-15.51	140	-15.51	22	867	696

204	hsa-miR-1207-3p	140	-15.46	140	-15.46	18	867	758
205	hsa-miR-4424	140	-15.46	140	-15.46	22	867	276
206	hsa-miR-548as-3p	147	-15.37	147	-15.37	22	867	364
207	hsa-miR-6875-3p	148	-15.25	148	-15.25	22	867	37
208	hsa-miR-5701	143	-15.24	143	-15.24	19	867	120
209	hsa-miR-2278	147	-15.17	147	-15.17	22	867	649
210	hsa-miR-320a	140	-15.14	140	-15.14	22	867	563
211	hsa-miR-511-5p	158	-15.13	158	-15.13	21	867	62
212	hsa-miR-548p	152	-15.09	152	-15.09	22	867	832
213	hsa-miR-186-3p	140	-15.03	140	-15.03	22	867	383
214	hsa-miR-4762-3p	148	-15.02	148	-15.02	22	867	177
215	hsa-miR-5699-3p	140	-14.95	140	-14.95	22	867	158
216	hsa-miR-509-5p	149	-14.85	149	-14.85	21	867	792
217	hsa-miR-4670-3p	140	-14.85	140	-14.85	22	867	49
218	hsa-miR-374c-3p	142	-14.8	142	-14.8	22	867	757
219	hsa-miR-539-5p	142	-14.73	142	-14.73	22	867	788
220	hsa-miR-2053	147	-14.67	147	-14.67	23	867	226
221	hsa-miR-5695	142	-14.61	142	-14.61	22	867	724
222	hsa-miR-3148	159	-14.6	159	-14.6	22	867	512
223	hsa-miR-1273e	146	-14.58	146	-14.58	22	867	797
224	hsa-miR-548av-3p	158	-14.55	158	-14.55	20	867	795
225	hsa-miR-3925-3p	147	-14.55	147	-14.55	21	867	693
226	hsa-miR-875-3p	143	-14.51	143	-14.51	21	867	208
227	hsa-miR-3915	149	-14.5	149	-14.5	22	867	75
228	hsa-miR-6507-5p	154	-14.49	154	-14.49	21	867	75
229	hsa-miR-655-5p	152	-14.46	152	-14.46	22	867	149
230	hsa-miR-4774-3p	148	-14.45	148	-14.45	21	867	312
231	hsa-miR-4491	142	-14.39	142	-14.39	22	867	257
232	hsa-miR-7154-5p	145	-14.38	145	-14.38	23	867	403
233	hsa-miR-1323	142	-14.37	142	-14.37	22	867	796
234	hsa-miR-1252-5p	148	-14.35	148	-14.35	22	867	447
235	hsa-miR-4704-3p	142	-14.29	142	-14.29	22	867	814
236	hsa-miR-550a-3-5p	145	-14.26	145	-14.26	20	867	113
237	hsa-miR-378j	145	-14.22	145	-14.22	19	867	754
238	hsa-miR-6508-5p	147	-14.21	147	-14.21	21	867	848
239	hsa-miR-549a	140	-14.11	140	-14.11	21	867	601
240	hsa-miR-22-5p	152	-14.07	152	-14.07	22	867	36

241	hsa-miR-3074-5p	147	-14.07	147	-14.07	21	867	57
242	hsa-miR-5584-5p	140	-14.03	140	-14.03	22	867	263
243	hsa-miR-630	141	-14.02	141	-14.02	22	867	670
244	hsa-miR-548ah-3p	148	-13.9	148	-13.9	22	867	795
245	hsa-miR-1287-5p	142	-13.83	142	-13.83	22	867	751
246	hsa-miR-4659a-5p	151	-13.81	151	-13.81	22	867	114
247	hsa-miR-548o-3p	148	-13.66	148	-13.66	22	867	795
248	hsa-miR-545-3p	154	-13.63	154	-13.63	22	867	836
249	hsa-miR-548am-3p	148	-13.49	148	-13.49	22	867	795
250	hsa-miR-499a-5p	145	-13.47	145	-13.47	21	867	797
251	hsa-miR-4287	140	-13.25	140	-13.25	19	867	349
252	hsa-miR-4524a-5p	146	-13.22	146	-13.22	22	867	663
253	hsa-miR-154-5p	151	-13.18	151	-13.18	22	867	151
254	hsa-miR-1290	145	-13.12	145	-13.12	19	867	752
255	hsa-miR-3613-3p	158	-13.04	158	-13.04	24	867	568
256	hsa-miR-4418	148	-12.9	148	-12.9	18	867	795
257	hsa-miR-548l	146	-12.87	146	-12.87	22	867	248
258	hsa-miR-4255	151	-12.86	151	-12.86	17	867	5
259	hsa-miR-515-5p	145	-12.67	145	-12.67	24	867	383
260	hsa-miR-6839-5p	140	-12.49	140	-12.49	22	867	751
261	hsa-miR-576-3p	148	-12.47	148	-12.47	22	867	257
262	hsa-miR-634	140	-12.15	140	-12.15	22	867	837
263	hsa-miR-8060	145	-12.12	145	-12.12	24	867	402
264	hsa-miR-3977	140	-12.03	140	-12.03	23	867	818
265	hsa-miR-4317	140	-12.02	140	-12.02	17	867	318
266	hsa-miR-485-3p	145	-11.98	145	-11.98	22	867	470
267	hsa-miR-6128	146	-11.95	146	-11.95	19	867	108
268	hsa-miR-4678	142	-11.94	142	-11.94	22	867	250
269	hsa-miR-4282	286	-11.75	146	-9.68	18	867	71 511
270	hsa-miR-6750-3p	143	-11.71	143	-11.71	21	867	487
271	hsa-miR-1278	147	-11.63	147	-11.63	22	867	645
272	hsa-miR-4275	142	-11.62	142	-11.62	17	867	738
273	hsa-miR-548ae-3p	142	-11.56	142	-11.56	21	867	793
274	hsa-miR-539-3p	143	-11.44	143	-11.44	22	867	470
275	hsa-miR-5007-3p	157	-11.4	157	-11.4	22	867	431
276	hsa-miR-651-3p	153	-11.4	153	-11.4	22	867	381
277	hsa-miR-6883-3p	144	-11.37	144	-11.37	22	867	340

278	hsa-miR-5093	146	-11.36	146	-11.36	23	867	260
279	hsa-miR-4714-5p	142	-11.34	142	-11.34	22	867	178
280	hsa-miR-32-3p	142	-11.27	142	-11.27	22	867	504
281	hsa-miR-4524b-5p	140	-11.24	140	-11.24	21	867	664
282	hsa-miR-4760-3p	140	-11.21	140	-11.21	22	867	436
283	hsa-miR-586	154	-11.19	154	-11.19	22	867	674
284	hsa-miR-944	151	-11.13	151	-11.13	22	867	101
285	hsa-miR-135b-5p	142	-11.04	142	-11.04	23	867	411
286	hsa-miR-577	145	-11.02	145	-11.02	21	867	201
287	hsa-miR-548x-3p	141	-11.02	141	-11.02	20	867	794
288	hsa-miR-133a-5p	140	-10.87	140	-10.87	22	867	493
289	hsa-miR-5700	151	-10.76	151	-10.76	22	867	675
290	hsa-miR-548aj-3p	142	-10.74	142	-10.74	21	867	793
291	hsa-miR-548aq-3p	142	-10.74	142	-10.74	22	867	792
292	hsa-miR-556-3p	142	-10.71	142	-10.71	22	867	187
293	hsa-miR-4668-5p	140	-10.68	140	-10.68	23	867	511
294	hsa-miR-4719	142	-10.58	142	-10.58	22	867	814
295	hsa-miR-7159-5p	140	-10.52	140	-10.52	21	867	738
296	hsa-miR-135a-5p	143	-10.43	143	-10.43	23	867	409
297	hsa-miR-3163	288	-10.25	148	-6.63	22	867	201 71
298	hsa-miR-3145-5p	149	-10.15	149	-10.15	22	867	725
299	hsa-miR-548j-3p	147	-10.15	147	-10.15	21	867	796
300	hsa-miR-4698	150	-10.04	150	-10.04	23	867	831
301	hsa-miR-372-5p	148	-9.98	148	-9.98	23	867	23
302	hsa-miR-3140-5p	140	-9.96	140	-9.96	21	867	416
303	hsa-miR-4668-3p	155	-9.95	155	-9.95	23	867	419
304	hsa-miR-15b-3p	144	-9.84	144	-9.84	22	867	102
305	hsa-miR-511-3p	141	-9.84	141	-9.84	20	867	197
306	hsa-miR-548az-3p	155	-9.83	155	-9.83	21	867	795
307	hsa-miR-513a-3p	142	-9.67	142	-9.67	23	867	782
308	hsa-miR-513c-3p	142	-9.67	142	-9.67	23	867	782
309	hsa-miR-633	140	-9.57	140	-9.57	23	867	663
310	hsa-miR-302c-5p	146	-9.28	146	-9.28	22	867	277
311	hsa-miR-19a-3p	144	-9.05	144	-9.05	23	867	272
312	hsa-miR-1468-3p	140	-9.05	140	-9.05	22	867	833
313	hsa-miR-2115-3p	141	-9.04	141	-9.04	22	867	653
314	hsa-miR-548f-3p	152	-9.01	152	-9.01	19	867	794

315	hsa-miR-3646	141	-8.9	141	-8.9	22	867	202
316	hsa-miR-548e-3p	151	-8.6	151	-8.6	22	867	795
317	hsa-miR-548a-3p	158	-8.27	158	-8.27	22	867	792
318	hsa-miR-576-5p	140	-7.78	140	-7.78	22	867	669
319	hsa-miR-126-5p	141	-6.57	141	-6.57	21	867	99
320	hsa-miR-3607-3p	140	-5.96	140	-5.96	20	867	579
321	hsa-miR-8066	140	-5.88	140	-5.88	21	867	259
322	hsa-miR-548n	143	-5.55	143	-5.55	22	867	251

Supplementary Table 7. 681 miRNAs potentially targeting JAK2

miRNA	Position in the UTR	seed match	context++ score	context++ score percentile	weighted context++ score	conserved branch length	Pct
Conserved sites							
hsa-miR-101-3p.1	609-616	8mer	-0.32	97	-0.32	4.565	0.75
hsa-miR-101-3p.2	610-617	8mer	-0.09	83	-0.09	4.863	0.37
hsa-miR-144-3p	610-616	7mer-1A	-0.06	71	-0.06	4.863	0.37
hsa-miR-582-5p	611-617	7mer-1A	-0.07	76	-0.07	3.613	N/A
hsa-miR-211-5p	631-638	8mer	-0.3	97	-0.3	4.521	0.46
hsa-miR-204-5p	631-638	8mer	-0.31	97	-0.31	4.521	0.46
hsa-miR-375	636-642	7mer-1A	-0.04	62	-0.04	4.931	0.35
hsa-miR-300	743-749	7mer-m8	-0.02	67	-0.02	2.867	N/A
hsa-miR-381-3p	743-749	7mer-m8	-0.02	65	-0.02	2.867	N/A
hsa-miR-216a-5p	833-840	8mer	-0.16	91	-0.16	2.267	< 0.1
hsa-miR-135a-5p	935-942	8mer	-0.32	96	-0.32	2.893	0.42
hsa-miR-135b-5p	935-942	8mer	-0.32	96	-0.32	2.893	0.42
Poorly conserved sites							
hsa-miR-4330	17-23	7mer-1A	-0.08	81	-0.08	0	N/A
hsa-miR-6848-3p	20-27	8mer	-0.27	98	-0.27	0	N/A

hsa-miR-6843-3p	20-27	8mer	-0.35	98	-0.35	0	N/A
hsa-miR-133b	22-28	7mer-1A	-0.1	69	-0.1	2.899	0.29
hsa-miR-133a-3p.2	22-28	7mer-1A	-0.1	68	-0.1	2.899	0.29
hsa-miR-9-5p	23-29	7mer-m8	-0.11	85	-0.11	1.736	< 0.1
hsa-miR-138-1-3p	27-33	7mer-1A	-0.02	67	-0.02	0	N/A
hsa-miR-3607-3p	34-41	8mer	-0.19	95	-0.19	0	N/A
hsa-miR-3686	35-42	8mer	-0.18	89	-0.18	0	N/A
hsa-miR-6739-3p	39-46	8mer	-0.2	91	-0.2	0	N/A
hsa-miR-4677-5p	39-45	7mer-1A	-0.05	72	-0.05	0.048	N/A
hsa-miR-4273	39-45	7mer-1A	-0.01	47	-0.01	0	N/A
hsa-miR-7156-5p	39-45	7mer-1A	-0.01	40	-0.01	0	N/A
hsa-miR-375	40-46	7mer-1A	-0.14	93	-0.14	3.584	0.33
hsa-miR-5582-3p	45-52	8mer	-0.09	95	-0.09	0	N/A
hsa-miR-548e-3p	46-52	7mer-1A	-0.01	57	-0.01	0.013	N/A
hsa-miR-548az-3p	46-52	7mer-1A	-0.01	57	-0.01	0.013	N/A
hsa-miR-548f-3p	46-52	7mer-1A	-0.01	57	-0.01	0.013	N/A
hsa-miR-548a-3p	46-52	7mer-1A	-0.01	47	-0.01	0.013	N/A
hsa-miR-548ar-3p	46-52	7mer-1A	-0.01	46	-0.01	0.013	N/A
hsa-miR-3163	48-54	7mer-1A	-0.01	45	-0.01	0	N/A
hsa-miR-8066	57-63	7mer-m8	-0.03	55	-0.03	0	N/A
hsa-miR-3908	60-66	7mer-m8	-0.03	71	-0.03	0	N/A
hsa-miR-3120-3p	62-68	7mer-m8	-0.03	48	-0.03	0.27	N/A
hsa-miR-140-3p.1	65-71	7mer-1A	-0.11	80	-0.11	3.506	0.17
hsa-miR-633	71-77	7mer-m8	-0.1	82	-0.1	0.048	N/A
hsa-miR-2054	76-82	7mer-m8	-0.08	83	-0.08	0	N/A
hsa-miR-380-3p	77-84	8mer	-0.21	97	-0.21	0.277	N/A
hsa-miR-4495	78-84	7mer-1A	-0.12	89	-0.12	0	N/A
hsa-miR-379-3p	78-84	7mer-1A	-0.09	88	-0.09	1.271	N/A
hsa-miR-411-3p	78-84	7mer-1A	-0.09	88	-0.09	1.271	N/A
hsa-miR-580-5p	86-92	7mer-1A	-0.03	70	-0.03	0	N/A
hsa-miR-5692b	91-97	7mer-1A	-0.06	90	-0.06	0	N/A
hsa-miR-374a-5p	91-97	7mer-m8	-0.08	90	-0.08	0.492	N/A
hsa-miR-5692c	91-97	7mer-1A	-0.06	90	-0.06	0	N/A
hsa-miR-374b-5p	91-97	7mer-m8	-0.04	75	-0.04	0.492	N/A
hsa-miR-369-3p	91-97	7mer-1A	-0.01	53	-0.01	0.492	N/A
hsa-miR-410-3p	93-99	7mer-1A	-0.01	55	-0.01	0.699	N/A
hsa-miR-190a-3p	94-100	7mer-1A	-0.02	40	-0.02	0.048	N/A

hsa-miR-6083	95-101	7mer-1A	-0.01	45	-0.01	0	N/A
hsa-miR-433-3p	101-107	7mer-m8	-0.1	84	-0.1	2.507	N/A
hsa-miR-621	108-114	7mer-m8	-0.19	78	-0.19	0.013	N/A
hsa-miR-6069	110-116	7mer-1A	-0.11	67	-0.11	0	N/A
hsa-miR-664a-5p	111-117	7mer-m8	-0.13	82	-0.13	0	N/A
hsa-miR-4794	111-117	7mer-m8	-0.09	78	-0.09	0	N/A
hsa-miR-5580-5p	112-119	8mer	-0.38	97	-0.38	0.048	N/A
hsa-miR-23a-3p	123-129	7mer-1A	-0.04	62	-0.04	2.125	< 0.1
hsa-miR-23b-3p	123-129	7mer-1A	-0.04	62	-0.04	2.125	< 0.1
hsa-miR-23c	123-129	7mer-1A	-0.04	61	-0.04	2.125	< 0.1
hsa-miR-130a-5p	123-129	7mer-1A	-0.03	58	-0.03	2.125	< 0.1
hsa-miR-5096	125-131	7mer-1A	-0.01	44	-0.01	0	N/A
hsa-miR-3145-3p	129-135	7mer-m8	-0.02	23	-0.02	0	N/A
hsa-miR-4468	134-141	8mer	-0.3	96	-0.3	0	N/A
hsa-miR-8055	137-144	8mer	-0.13	94	-0.13	0	N/A
hsa-miR-2052	139-145	7mer-m8	-0.02	30	-0.02	0	N/A
hsa-miR-561-3p	142-148	7mer-m8	-0.02	54	-0.02	0	N/A
hsa-miR-488-3p	145-151	7mer-1A	-0.03	64	-0.03	1.302	N/A
hsa-miR-510-3p	146-152	7mer-1A	-0.01	43	-0.01	0	N/A
hsa-miR-5582-3p	160-166	7mer-m8	-0.02	65	-0.02	0	N/A
hsa-miR-548x-3p	161-167	7mer-m8	-0.02	67	-0.02	0.048	N/A
hsa-miR-548aq-3p	161-167	7mer-m8	-0.02	67	-0.02	0.048	N/A
hsa-miR-548ah-3p	161-167	7mer-m8	-0.02	67	-0.02	0.048	N/A
hsa-miR-548am-3p	161-167	7mer-m8	-0.02	67	-0.02	0.048	N/A
hsa-miR-548j-3p	161-167	7mer-m8	-0.02	67	-0.02	0.048	N/A
hsa-miR-548ae-3p	161-167	7mer-m8	-0.02	67	-0.02	0.048	N/A
hsa-miR-548aj-3p	161-167	7mer-m8	-0.02	67	-0.02	0.048	N/A
hsa-miR-3185	166-172	7mer-1A	-0.02	44	-0.02	0	N/A
hsa-miR-8060	168-175	8mer	-0.16	93	-0.16	0	N/A
hsa-miR-7154-5p	169-175	7mer-m8	-0.02	28	-0.02	0	N/A
hsa-miR-6878-3p	174-180	7mer-m8	-0.2	88	-0.2	0	N/A
hsa-miR-4757-5p	174-180	7mer-1A	-0.14	84	-0.14	0	N/A
hsa-miR-6744-3p	174-180	7mer-1A	-0.07	80	-0.07	0	N/A
hsa-miR-4456	178-184	7mer-m8	-0.19	88	-0.19	0	N/A
hsa-miR-511-5p	187-193	7mer-m8	-0.08	88	-0.08	0.013	N/A
hsa-miR-6830-3p	187-193	7mer-1A	-0.01	29	-0.01	0	N/A
hsa-miR-513b-3p	190-196	7mer-1A	-0.14	87	-0.14	0	N/A

hsa-miR-6758-3p	196-202	7mer-1A	-0.03	67	-0.03	0	N/A
hsa-miR-5571-5p	199-205	7mer-m8	-0.02	79	-0.02	0	N/A
hsa-miR-651-3p	204-210	7mer-1A	-0.06	79	-0.06	0	N/A
hsa-miR-4521	205-211	7mer-m8	-0.06	71	-0.06	0	N/A
hsa-miR-4639-5p	207-214	8mer	-0.14	87	-0.14	0	N/A
hsa-miR-502-5p	211-217	7mer-m8	-0.19	91	-0.19	0.054	N/A
hsa-miR-6841-3p	211-217	7mer-1A	-0.18	80	-0.18	0	N/A
hsa-miR-1915-5p	211-217	7mer-1A	-0.04	47	-0.04	0	N/A
hsa-miR-500b-5p	212-218	7mer-m8	-0.1	77	-0.1	0.032	N/A
hsa-miR-362-5p	212-218	7mer-m8	-0.1	77	-0.1	0.032	N/A
hsa-miR-500a-5p	213-219	7mer-m8	-0.04	67	-0.04	0.013	N/A
hsa-miR-4668-3p	215-221	7mer-m8	-0.02	74	-0.02	0	N/A
hsa-miR-8063	216-222	7mer-m8	-0.02	77	-0.02	0	N/A
hsa-miR-520d-5p	219-225	7mer-1A	-0.01	52	-0.01	0.048	N/A
hsa-miR-524-5p	219-225	7mer-1A	-0.01	51	-0.01	0.048	N/A
hsa-miR-600	221-227	7mer-1A	-0.1	76	-0.1	0.013	N/A
hsa-miR-3658	231-238	8mer	-0.03	60	-0.03	0	N/A
hsa-miR-3653-3p	231-237	7mer-1A	-0.01	42	-0.01	0	N/A
hsa-miR-33a-3p	236-242	7mer-m8	-0.02	49	-0.02	0	N/A
hsa-miR-888-3p	242-248	7mer-m8	-0.19	92	-0.19	0	N/A
hsa-miR-4765	253-259	7mer-m8	-0.16	88	-0.16	0	N/A
hsa-miR-5586-3p	254-260	7mer-m8	-0.18	89	-0.18	0	N/A
hsa-miR-1202	264-270	7mer-1A	-0.09	84	-0.09	0.013	N/A
hsa-miR-5006-5p	264-270	7mer-1A	-0.13	76	-0.13	0	N/A
hsa-miR-3972	264-270	7mer-1A	-0.09	75	-0.09	0.013	N/A
hsa-miR-4776-3p	265-271	7mer-1A	-0.04	51	-0.04	0	N/A
hsa-miR-138-2-3p	279-285	7mer-1A	-0.01	48	-0.01	0.417	N/A
hsa-miR-219a-1-3p	291-298	8mer	-0.22	89	-0.22	0.013	N/A
hsa-miR-571	292-298	7mer-1A	-0.09	61	-0.09	0.013	N/A
hsa-miR-4682	293-299	7mer-m8	-0.06	71	-0.06	0	N/A
hsa-miR-8077	294-300	7mer-m8	-0.12	93	-0.12	0	N/A
hsa-miR-320a	297-303	7mer-m8	-0.1	85	-0.1	1.045	N/A
hsa-miR-320d	297-303	7mer-m8	-0.1	85	-0.1	1.045	N/A
hsa-miR-320c	297-303	7mer-m8	-0.1	85	-0.1	1.045	N/A
hsa-miR-320b	297-303	7mer-m8	-0.1	85	-0.1	1.045	N/A
hsa-miR-4429	297-303	7mer-m8	-0.07	80	-0.07	1.045	N/A
hsa-miR-888-5p	297-308	non-canonical	N/A	N/A	N/A	0	N/A

hsa-miR-888-5p	297-308	non-canonical	N/A	N/A	N/A	0	N/A
hsa-miR-3613-3p	301-307	7mer-1A	-0.01	32	-0.01	0	N/A
hsa-miR-373-5p	302-309	8mer	-0.03	57	-0.03	0.369	N/A
hsa-miR-371b-5p	302-309	8mer	-0.03	57	-0.03	0.369	N/A
hsa-miR-616-5p	302-309	8mer	-0.03	52	-0.03	0.369	N/A
hsa-miR-371a-5p	303-309	7mer-1A	-0.01	41	-0.01	2.528	N/A
hsa-miR-372-5p	303-309	7mer-1A	-0.01	34	-0.01	0.443	N/A
hsa-miR-30e-3p	311-317	7mer-1A	-0.01	38	-0.01	0.013	N/A
hsa-miR-30a-3p	311-317	7mer-1A	-0.01	38	-0.01	0.013	N/A
hsa-miR-30d-3p	311-317	7mer-1A	-0.01	38	-0.01	0.013	N/A
hsa-miR-335-3p	312-318	7mer-1A	-0.01	40	-0.01	0.048	N/A
hsa-miR-590-3p	316-322	7mer-1A	-0.02	76	-0.02	0.395	N/A
hsa-miR-4775	316-322	7mer-m8	-0.02	47	-0.02	0	N/A
hsa-miR-4282	329-335	7mer-m8	-0.02	66	-0.02	0	N/A
hsa-miR-1468-3p	331-338	8mer	-0.2	96	-0.2	0	N/A
hsa-miR-548aw	332-339	8mer	-0.14	97	-0.14	0	N/A
hsa-miR-548f-5p	332-338	7mer-1A	-0.04	69	-0.04	0	N/A
hsa-miR-548g-5p	332-338	7mer-1A	-0.01	47	-0.01	0	N/A
hsa-miR-548x-5p	332-338	7mer-1A	-0.01	47	-0.01	0	N/A
hsa-miR-548aj-5p	332-338	7mer-1A	-0.01	47	-0.01	0	N/A
hsa-miR-527	333-339	7mer-1A	-0.03	67	-0.03	0.013	N/A
hsa-miR-518a-5p	333-339	7mer-1A	-0.03	67	-0.03	0.013	N/A
hsa-miR-3143	338-345	8mer	-0.03	62	-0.03	0	N/A
hsa-miR-543	338-344	7mer-1A	-0.01	53	-0.01	0.818	N/A
hsa-miR-302c-5p	339-346	8mer	-0.03	73	-0.03	0	N/A
hsa-miR-302d-5p	341-348	8mer	-0.03	86	-0.03	0	N/A
hsa-miR-302b-5p	341-348	8mer	-0.03	84	-0.03	0	N/A
hsa-miR-5008-3p	350-356	7mer-1A	-0.1	75	-0.1	0.245	N/A
hsa-miR-7157-3p	350-356	7mer-1A	-0.09	74	-0.09	0.245	N/A
hsa-miR-6737-3p	350-356	7mer-1A	-0.08	71	-0.08	0.245	N/A
hsa-miR-6889-3p	350-356	7mer-1A	-0.08	69	-0.08	0.271	N/A
hsa-miR-4778-5p	352-359	8mer	-0.18	95	-0.18	0	N/A
hsa-miR-5580-3p	357-363	7mer-1A	-0.01	43	-0.01	0	N/A
hsa-miR-1284	364-370	7mer-m8	-0.17	81	-0.17	0.013	N/A
hsa-miR-548x-3p	369-376	8mer	-0.03	84	-0.03	0.048	N/A
hsa-miR-548aj-3p	369-376	8mer	-0.03	84	-0.03	0.048	N/A
hsa-miR-548ah-3p	369-376	8mer	-0.03	84	-0.03	0.048	N/A

hsa-miR-548j-3p	369-376	8mer	-0.03	84	-0.03	0.048	N/A
hsa-miR-548am-3p	369-376	8mer	-0.03	84	-0.03	0.048	N/A
hsa-miR-548ae-3p	369-376	8mer	-0.03	84	-0.03	0.048	N/A
hsa-miR-548aq-3p	369-376	8mer	-0.03	84	-0.03	0.048	N/A
hsa-miR-548h-3p	370-376	7mer-1A	-0.01	57	-0.01	0.297	N/A
hsa-miR-548bb-3p	370-376	7mer-1A	-0.01	57	-0.01	0.297	N/A
hsa-miR-548d-3p	370-376	7mer-1A	-0.01	57	-0.01	0.297	N/A
hsa-miR-548z	370-376	7mer-1A	-0.01	57	-0.01	0.297	N/A
hsa-miR-548ac	370-376	7mer-1A	-0.01	57	-0.01	0.297	N/A
hsa-miR-4536-5p	375-381	7mer-1A	-0.15	68	-0.15	0.054	N/A
hsa-miR-4318	377-383	7mer-m8	-0.14	83	-0.14	0	N/A
hsa-miR-181a-2-3p	380-386	7mer-1A	-0.17	90	-0.17	0	N/A
hsa-miR-6509-3p	380-386	7mer-m8	-0.16	84	-0.16	0	N/A
hsa-miR-1273g-3p	380-386	7mer-1A	-0.09	71	-0.09	0	N/A
hsa-let-7a-3p	387-393	7mer-1A	-0.17	95	-0.17	0.389	N/A
hsa-let-7b-3p	387-393	7mer-1A	-0.14	93	-0.14	0.389	N/A
hsa-miR-98-3p	387-393	7mer-1A	-0.12	88	-0.12	0.389	N/A
hsa-let-7f-1-3p	387-393	7mer-1A	-0.11	86	-0.11	0.389	N/A
hsa-let-7f-2-3p	387-393	7mer-1A	-0.09	84	-0.09	0.517	N/A
hsa-miR-1185-1-3p	387-393	7mer-1A	-0.02	51	-0.02	0.517	N/A
hsa-miR-4789-5p	387-393	7mer-1A	-0.01	51	-0.01	0.363	N/A
hsa-miR-1185-2-3p	387-393	7mer-1A	-0.02	51	-0.02	0.517	N/A
hsa-miR-4678	392-398	7mer-m8	-0.11	76	-0.11	0	N/A
hsa-miR-4480	397-403	7mer-1A	-0.15	87	-0.15	0	N/A
hsa-miR-4731-3p	405-411	7mer-m8	-0.02	59	-0.02	0	N/A
hsa-miR-4801	405-411	7mer-m8	-0.02	54	-0.02	0	N/A
hsa-miR-377-3p	407-413	7mer-m8	-0.02	22	-0.02	2.614	N/A
hsa-miR-499b-3p	410-416	7mer-m8	-0.04	67	-0.04	0.054	N/A
hsa-miR-499a-3p	410-416	7mer-m8	-0.04	67	-0.04	0.054	N/A
hsa-miR-6867-5p	420-426	7mer-1A	-0.05	34	-0.05	0	N/A
hsa-miR-3607-5p	423-429	7mer-1A	-0.07	78	-0.07	0	N/A
hsa-miR-4687-5p	428-434	7mer-m8	-0.12	83	-0.12	0	N/A
hsa-miR-1226-3p	432-438	7mer-m8	-0.15	86	-0.15	0.054	N/A
hsa-miR-21-3p	434-440	7mer-m8	-0.02	22	-0.02	0.048	N/A
hsa-miR-3591-3p	434-440	7mer-m8	-0.02	18	-0.02	0.048	N/A
hsa-miR-16-1-3p	445-451	7mer-m8	-0.08	78	-0.08	0.048	N/A
hsa-miR-144-3p	446-452	7mer-m8	-0.14	88	-0.14	0.16	< 0.1

hsa-miR-570-3p	450-456	7mer-m8	-0.08	76	-0.08	0	N/A
hsa-miR-3148	460-467	8mer	-0.11	90	-0.11	0	N/A
hsa-miR-6124	461-467	7mer-1A	-0.01	66	-0.01	0	N/A
hsa-miR-3688-3p	462-469	8mer	-0.12	86	-0.12	0.363	N/A
hsa-miR-944	478-484	7mer-1A	-0.01	41	-0.01	0.013	N/A
hsa-miR-548ao-5p	486-493	8mer	-0.23	93	-0.23	0	N/A
hsa-miR-548ax	486-493	8mer	-0.23	93	-0.23	0	N/A
hsa-miR-5585-5p	487-493	7mer-1A	-0.06	70	-0.06	0	N/A
hsa-miR-3606-5p	490-496	7mer-m8	-0.17	83	-0.17	0	N/A
hsa-miR-568	495-501	7mer-1A	-0.07	69	-0.07	0.251	N/A
hsa-miR-3149	496-502	7mer-1A	-0.05	71	-0.05	0.013	N/A
hsa-miR-8068	498-504	7mer-1A	-0.12	78	-0.12	0	N/A
hsa-miR-4775	504-510	7mer-1A	-0.01	30	-0.01	0	N/A
hsa-miR-4539	514-520	7mer-1A	-0.05	75	-0.05	0	N/A
hsa-miR-3200-5p	516-522	7mer-1A	-0.14	87	-0.14	0	N/A
hsa-miR-4275	521-527	7mer-1A	-0.02	62	-0.02	0	N/A
hsa-miR-4272	524-530	7mer-1A	-0.01	53	-0.01	0	N/A
hsa-miR-548m	533-539	7mer-m8	-0.02	37	-0.02	0	N/A
hsa-miR-1197	540-546	7mer-m8	-0.25	95	-0.25	1.636	N/A
hsa-miR-8080	541-547	7mer-m8	-0.02	30	-0.02	0	N/A
hsa-miR-3646	549-556	8mer	-0.03	77	-0.03	0	N/A
hsa-miR-1252-3p	550-556	7mer-1A	-0.01	53	-0.01	0	N/A
hsa-miR-4435	562-568	7mer-m8	-0.08	66	-0.08	0	N/A
hsa-miR-3190-5p	563-569	7mer-m8	-0.1	72	-0.1	0	N/A
hsa-miR-5580-5p	565-571	7mer-1A	-0.12	70	-0.12	0.048	N/A
hsa-miR-155-5p	568-574	7mer-m8	-0.04	47	-0.04	0.703	< 0.1
hsa-miR-374a-5p	571-577	7mer-1A	-0.01	30	-0.01	0.703	N/A
hsa-miR-374b-5p	571-577	7mer-1A	-0.01	30	-0.01	0.703	N/A
hsa-miR-4789-5p	582-588	7mer-m8	-0.02	73	-0.02	0	N/A
hsa-miR-548l	586-592	7mer-m8	-0.02	58	-0.02	0.013	N/A
hsa-miR-548n	587-594	8mer	-0.03	74	-0.03	0.054	N/A
hsa-miR-548az-5p	588-594	7mer-1A	-0.04	84	-0.04	0	N/A
hsa-miR-548t-5p	588-594	7mer-1A	-0.04	84	-0.04	0	N/A
hsa-miR-4802-3p	604-611	8mer	-0.14	85	-0.14	0	N/A
hsa-miR-942-3p	605-611	7mer-1A	-0.06	71	-0.06	0	N/A
hsa-miR-4699-3p	614-620	7mer-1A	-0.04	73	-0.04	0	N/A
hsa-miR-4501	625-631	7mer-1A	-0.04	69	-0.04	0	N/A

hsa-miR-6832-3p	631-637	7mer-1A	-0.03	56	-0.03	0.572	N/A
hsa-miR-5006-3p	632-638	7mer-m8	-0.1	84	-0.1	0	N/A
hsa-miR-4755-5p	632-638	7mer-m8	-0.09	82	-0.09	0	N/A
hsa-miR-623	632-638	7mer-1A	-0.08	70	-0.08	0	N/A
hsa-miR-642b-5p	633-640	8mer	-0.39	98	-0.39	0	N/A
hsa-miR-6739-3p	636-642	7mer-1A	-0.03	41	-0.03	0	N/A
hsa-miR-4305	643-649	7mer-m8	-0.17	76	-0.17	0	N/A
hsa-miR-708-3p	645-651	7mer-m8	-0.06	59	-0.06	0.443	N/A
hsa-miR-7844-5p	646-652	7mer-m8	-0.12	94	-0.12	0	N/A
hsa-miR-5582-3p	648-654	7mer-1A	-0.03	89	-0.03	0.27	N/A
hsa-miR-548a-3p	648-654	7mer-1A	-0.01	47	-0.01	0.276	N/A
hsa-miR-548az-3p	648-654	7mer-1A	-0.01	46	-0.01	0.276	N/A
hsa-miR-548e-3p	648-654	7mer-1A	-0.01	46	-0.01	0.276	N/A
hsa-miR-548ar-3p	648-654	7mer-1A	-0.01	46	-0.01	0.276	N/A
hsa-miR-548f-3p	648-654	7mer-1A	-0.01	45	-0.01	0.276	N/A
hsa-miR-4666a-3p	656-662	7mer-1A	-0.04	82	-0.04	0	N/A
hsa-miR-300	656-662	7mer-1A	-0.03	79	-0.03	3.153	N/A
hsa-miR-98-3p	656-662	7mer-m8	-0.04	66	-0.04	0.596	N/A
hsa-let-7b-3p	656-662	7mer-m8	-0.02	50	-0.02	0.596	N/A
hsa-let-7a-3p	656-662	7mer-m8	-0.02	48	-0.02	0.596	N/A
hsa-miR-381-3p	656-662	7mer-1A	-0.01	48	-0.01	3.153	N/A
hsa-let-7f-1-3p	656-662	7mer-m8	-0.02	35	-0.02	0.596	N/A
hsa-miR-1284	657-663	7mer-m8	-0.12	67	-0.12	0.373	N/A
hsa-miR-337-3p	659-666	8mer	-0.13	77	-0.13	0.054	N/A
hsa-miR-202-5p	660-666	7mer-1A	-0.08	69	-0.08	1.948	< 0.1
hsa-miR-384	661-667	7mer-1A	-0.07	70	-0.07	2.022	N/A
hsa-miR-3685	661-667	7mer-1A	-0.01	39	-0.01	0	N/A
hsa-miR-3606-3p	664-670	7mer-m8	-0.02	78	-0.02	0	N/A
hsa-miR-513a-3p	664-670	7mer-m8	-0.02	78	-0.02	0	N/A
hsa-miR-513c-3p	664-670	7mer-m8	-0.02	78	-0.02	0	N/A
hsa-miR-5584-5p	668-674	7mer-m8	-0.02	30	-0.02	0	N/A
hsa-miR-6754-5p	670-676	7mer-1A	-0.09	73	-0.09	0	N/A
hsa-miR-4441	670-676	7mer-1A	-0.1	73	-0.1	0	N/A
hsa-miR-4270	670-676	7mer-1A	-0.03	52	-0.03	0	N/A
hsa-miR-211-3p	670-676	7mer-1A	-0.01	49	-0.01	0	N/A
hsa-miR-1178-5p	674-681	8mer	-0.3	95	-0.3	0	N/A
hsa-miR-1182	675-681	7mer-1A	-0.14	87	-0.14	0.369	N/A

hsa-miR-2909	677-683	7mer-1A	-0.04	64	-0.04	0	N/A
hsa-miR-126-5p	682-688	7mer-1A	-0.02	48	-0.02	0	N/A
hsa-miR-4795-3p	682-688	7mer-m8	-0.02	43	-0.02	0	N/A
hsa-miR-496.2	684-690	7mer-1A	-0.03	61	-0.03	0.383	N/A
hsa-miR-4698	689-696	8mer	-0.03	75	-0.03	0	N/A
hsa-miR-8063	690-696	7mer-1A	-0.01	54	-0.01	0	N/A
hsa-miR-607	692-698	7mer-1A	-0.01	40	-0.01	0	N/A
hsa-miR-579-3p	696-702	7mer-1A	-0.05	84	-0.05	0.054	N/A
hsa-miR-5696	696-702	7mer-1A	-0.02	63	-0.02	0	N/A
hsa-miR-664b-3p	696-702	7mer-1A	-0.01	42	-0.01	0.054	N/A
hsa-miR-100-3p	704-711	8mer	-0.28	98	-0.28	0	N/A
hsa-miR-6844	711-717	7mer-m8	-0.22	95	-0.22	0	N/A
hsa-miR-6083	716-722	7mer-m8	-0.06	93	-0.06	0	N/A
hsa-miR-3121-3p	723-729	7mer-m8	-0.02	48	-0.02	0.013	N/A
hsa-miR-3163	726-732	7mer-m8	-0.02	65	-0.02	0	N/A
hsa-miR-5584-5p	738-744	7mer-m8	-0.05	63	-0.05	0	N/A
hsa-miR-624-3p	742-748	7mer-1A	-0.08	76	-0.08	0.013	N/A
hsa-miR-3121-3p	749-755	7mer-m8	-0.02	48	-0.02	0.013	N/A
hsa-miR-4719	752-758	7mer-m8	-0.02	55	-0.02	0	N/A
hsa-miR-197-3p	756-763	8mer	-0.27	96	-0.27	0.054	N/A
hsa-miR-4262	760-766	7mer-m8	-0.02	43	-0.02	1.074	< 0.1
hsa-miR-181c-5p	760-766	7mer-m8	-0.02	31	-0.02	1.074	< 0.1
hsa-miR-181d-5p	760-766	7mer-m8	-0.02	30	-0.02	1.074	< 0.1
hsa-miR-181b-5p	760-766	7mer-m8	-0.02	30	-0.02	1.074	< 0.1
hsa-miR-181a-5p	760-766	7mer-m8	-0.02	30	-0.02	1.074	< 0.1
hsa-miR-620	784-791	8mer	-0.21	94	-0.21	0	N/A
hsa-miR-1270	784-791	8mer	-0.19	93	-0.19	0	N/A
hsa-miR-4683	784-790	7mer-1A	-0.05	65	-0.05	0	N/A
hsa-miR-4531	785-791	7mer-1A	-0.13	81	-0.13	0	N/A
hsa-miR-4282	790-796	7mer-m8	-0.02	66	-0.02	0	N/A
hsa-miR-1244	803-809	7mer-1A	-0.08	65	-0.08	0.013	N/A
hsa-miR-376a-3p	807-813	7mer-1A	-0.17	82	-0.17	0.115	N/A
hsa-miR-376b-3p	807-813	7mer-1A	-0.17	82	-0.17	0.115	N/A
hsa-miR-6853-3p	809-815	7mer-1A	-0.05	58	-0.05	0	N/A
hsa-miR-1206	809-815	7mer-1A	-0.06	56	-0.06	0.013	N/A
hsa-miR-548g-3p	813-819	7mer-m8	-0.04	76	-0.04	0	N/A
hsa-miR-548ar-3p	814-820	7mer-m8	-0.02	61	-0.02	0.276	N/A

hsa-miR-548a-3p	814-820	7mer-m8	-0.02	60	-0.02	0.276	N/A
hsa-miR-548f-3p	814-820	7mer-m8	-0.02	59	-0.02	0.276	N/A
hsa-miR-548az-3p	814-820	7mer-m8	-0.02	59	-0.02	0.276	N/A
hsa-miR-548e-3p	814-820	7mer-m8	-0.02	59	-0.02	0.276	N/A
hsa-miR-590-3p	825-831	7mer-m8	-0.02	59	-0.02	0.251	N/A
hsa-miR-4282	827-833	7mer-m8	-0.02	66	-0.02	0	N/A
hsa-miR-8063	829-835	7mer-1A	-0.01	54	-0.01	0	N/A
hsa-miR-4698	829-835	7mer-1A	-0.01	42	-0.01	0	N/A
hsa-miR-616-5p	830-837	8mer	-0.1	87	-0.1	0.523	N/A
hsa-miR-371b-5p	830-837	8mer	-0.08	87	-0.08	0.523	N/A
hsa-miR-373-5p	830-837	8mer	-0.07	84	-0.07	0.523	N/A
hsa-miR-372-5p	831-837	7mer-1A	-0.02	62	-0.02	0.417	N/A
hsa-miR-371a-5p	831-837	7mer-1A	-0.01	41	-0.01	2.749	N/A
hsa-miR-216b-5p	834-840	7mer-1A	-0.09	80	-0.09	2.267	< 0.1
hsa-miR-1256	843-849	7mer-1A	-0.16	81	-0.16	0.27	N/A
hsa-miR-4799-3p	844-850	7mer-m8	-0.15	87	-0.15	0	N/A
hsa-miR-4720-5p	844-850	7mer-m8	-0.14	86	-0.14	0	N/A
hsa-miR-5588-5p	844-850	7mer-m8	-0.13	85	-0.13	0	N/A
hsa-miR-8073	845-852	8mer	-0.33	98	-0.33	0	N/A
hsa-miR-221-5p	845-852	8mer	-0.36	98	-0.36	0	N/A
hsa-miR-1343-3p	847-853	7mer-1A	-0.09	69	-0.09	0.27	N/A
hsa-miR-6783-3p	847-853	7mer-1A	-0.08	66	-0.08	0.27	N/A
hsa-miR-195-3p	852-858	7mer-m8	-0.02	41	-0.02	0	N/A
hsa-miR-16-2-3p	852-858	7mer-m8	-0.02	40	-0.02	0	N/A
hsa-miR-338-5p	853-859	7mer-m8	-0.02	51	-0.02	0	N/A
hsa-miR-6744-5p	858-864	7mer-m8	-0.24	92	-0.24	0	N/A
hsa-miR-4660	868-874	7mer-m8	-0.22	91	-0.22	0.013	N/A
hsa-miR-646	869-875	7mer-m8	-0.08	73	-0.08	0.013	N/A
hsa-miR-922	871-877	7mer-1A	-0.08	87	-0.08	0.054	N/A
hsa-miR-214-3p	871-877	7mer-1A	-0.04	62	-0.04	0.054	N/A
hsa-miR-4291	871-877	7mer-m8	-0.04	53	-0.04	0	N/A
hsa-miR-761	871-877	7mer-1A	-0.01	45	-0.01	0.054	N/A
hsa-miR-3619-5p	871-877	7mer-1A	-0.01	36	-0.01	0.054	N/A
hsa-miR-34c-5p	877-883	7mer-1A	-0.19	75	-0.19	1.665	< 0.1
hsa-miR-34a-5p	877-883	7mer-1A	-0.18	74	-0.18	1.665	< 0.1
hsa-miR-449b-5p	877-883	7mer-1A	-0.14	65	-0.14	1.665	< 0.1
hsa-miR-548au-3p	877-883	7mer-1A	-0.08	64	-0.08	0	N/A

hsa-miR-449a	877-883	7mer-1A	-0.12	61	-0.12	1.665	< 0.1
hsa-miR-7150	878-884	7mer-1A	-0.1	70	-0.1	0	N/A
hsa-miR-4756-3p	897-903	7mer-m8	-0.02	52	-0.02	0	N/A
hsa-miR-605-5p	903-910	8mer	-0.08	81	-0.08	0.013	N/A
hsa-miR-433-3p	914-920	7mer-1A	-0.03	56	-0.03	0.3	N/A
hsa-miR-7154-5p	914-920	7mer-1A	-0.02	38	-0.02	0	N/A
hsa-miR-1206	915-921	7mer-m8	-0.13	81	-0.13	0.013	N/A
hsa-miR-5000-5p	917-923	7mer-1A	-0.09	79	-0.09	0	N/A
hsa-miR-32-3p	921-927	7mer-m8	-0.02	73	-0.02	0	N/A
hsa-miR-3606-3p	923-929	7mer-1A	-0.01	57	-0.01	0	N/A
hsa-miR-513c-3p	923-929	7mer-1A	-0.01	57	-0.01	0	N/A
hsa-miR-513a-3p	923-929	7mer-1A	-0.01	57	-0.01	0	N/A
hsa-miR-100-3p	929-935	7mer-1A	-0.12	86	-0.12	0	N/A
hsa-miR-4720-3p	932-938	7mer-m8	-0.09	74	-0.09	0	N/A
hsa-miR-889-5p	936-942	7mer-1A	-0.14	79	-0.14	0.048	N/A
hsa-miR-8074	937-943	7mer-1A	-0.2	87	-0.2	0	N/A
hsa-miR-138-2-3p	943-949	7mer-1A	-0.01	48	-0.01	0	N/A
hsa-miR-153-5p	964-970	7mer-m8	-0.02	50	-0.02	0	N/A
hsa-miR-7151-5p	968-974	7mer-1A	-0.1	88	-0.1	0	N/A
hsa-miR-610	973-979	7mer-1A	-0.23	90	-0.23	0.013	N/A
hsa-miR-556-5p	974-980	7mer-m8	-0.14	88	-0.14	0	N/A
hsa-miR-4325	985-991	7mer-m8	-0.07	76	-0.07	0	N/A
hsa-miR-4793-3p	986-992	7mer-m8	-0.13	89	-0.13	0	N/A
hsa-miR-370-3p	990-996	7mer-m8	-0.1	84	-0.1	0.041	N/A
hsa-miR-6893-3p	990-996	7mer-m8	-0.16	84	-0.16	0.041	N/A
hsa-miR-649	993-999	7mer-1A	-0.1	81	-0.1	0.054	N/A
hsa-miR-490-3p	993-999	7mer-1A	-0.09	76	-0.09	0.154	< 0.1
hsa-miR-3148	1005-1011	7mer-m8	-0.06	79	-0.06	0	N/A
hsa-miR-4496	1007-1013	7mer-1A	-0.01	64	-0.01	0	N/A
hsa-miR-651-5p	1009-1015	7mer-1A	-0.12	84	-0.12	0.013	N/A
hsa-miR-1185-2-3p	1018-1024	7mer-m8	-0.03	62	-0.03	0	N/A
hsa-miR-1185-1-3p	1018-1024	7mer-m8	-0.03	62	-0.03	0	N/A
hsa-let-7f-2-3p	1018-1024	7mer-m8	-0.02	54	-0.02	0	N/A
hsa-miR-3671	1023-1029	7mer-m8	-0.02	41	-0.02	0	N/A
hsa-miR-372-5p	1024-1030	7mer-m8	-0.02	63	-0.02	0	N/A
hsa-miR-6771-3p	1030-1036	7mer-m8	-0.18	90	-0.18	0	N/A
hsa-miR-4766-5p	1034-1040	7mer-1A	-0.02	59	-0.02	0	N/A

hsa-miR-1468-3p	1041-1048	8mer	-0.09	83	-0.09	0	N/A
hsa-miR-548aw	1042-1048	7mer-m8	-0.02	49	-0.02	0	N/A
hsa-miR-548f-5p	1042-1048	7mer-1A	-0.01	35	-0.01	0	N/A
hsa-miR-548aj-5p	1042-1048	7mer-1A	-0.01	35	-0.01	0	N/A
hsa-miR-548g-5p	1042-1048	7mer-1A	-0.01	35	-0.01	0	N/A
hsa-miR-548x-5p	1042-1048	7mer-1A	-0.01	35	-0.01	0	N/A
hsa-miR-106a-3p	1047-1053	7mer-m8	-0.09	77	-0.09	0	N/A
hsa-miR-5002-3p	1051-1057	7mer-1A	-0.09	65	-0.09	0	N/A
hsa-miR-7159-3p	1056-1062	7mer-m8	-0.03	65	-0.03	0	N/A
hsa-miR-6515-3p	1060-1066	7mer-1A	-0.15	89	-0.15	0	N/A
hsa-miR-1236-3p	1060-1066	7mer-1A	-0.03	57	-0.03	0.013	N/A
hsa-miR-216b-5p	1062-1068	7mer-m8	-0.1	82	-0.1	1.906	< 0.1
hsa-miR-651-3p	1071-1077	7mer-m8	-0.02	37	-0.02	0	N/A
hsa-miR-520f-5p	1078-1084	7mer-m8	-0.02	28	-0.02	0	N/A
hsa-miR-6826-3p	1082-1089	8mer	-0.36	96	-0.36	0	N/A
hsa-miR-6795-3p	1082-1088	7mer-1A	-0.14	81	-0.14	0	N/A
hsa-miR-6887-3p	1082-1088	7mer-1A	-0.05	47	-0.05	0	N/A
hsa-miR-3679-3p	1084-1090	7mer-1A	-0.08	67	-0.08	0	N/A
hsa-miR-4777-3p	1091-1098	8mer	-0.25	89	-0.25	0	N/A
hsa-miR-4699-3p	1095-1101	7mer-1A	-0.01	32	-0.01	0	N/A
hsa-miR-2681-5p	1103-1109	7mer-1A	-0.01	53	-0.01	0	N/A
hsa-miR-5003-3p	1107-1113	7mer-1A	-0.01	46	-0.01	0	N/A
hsa-miR-1-5p	1109-1115	7mer-m8	-0.16	86	-0.16	0.048	< 0.1
hsa-miR-4282	1122-1128	7mer-1A	-0.01	49	-0.01	0	N/A
hsa-miR-3163	1123-1129	7mer-m8	-0.02	65	-0.02	0	N/A
hsa-miR-5585-3p	1127-1133	7mer-1A	-0.05	75	-0.05	0	N/A
hsa-miR-4635	1129-1135	7mer-1A	-0.07	84	-0.07	0	N/A
hsa-miR-526b-5p	1130-1136	7mer-1A	-0.09	75	-0.09	0.054	N/A
hsa-miR-1256	1135-1141	7mer-1A	-0.1	66	-0.1	0.276	N/A
hsa-miR-4720-5p	1136-1142	7mer-m8	-0.22	94	-0.22	0	N/A
hsa-miR-5588-5p	1136-1142	7mer-m8	-0.21	94	-0.21	0	N/A
hsa-miR-4799-3p	1136-1142	7mer-m8	-0.21	93	-0.21	0	N/A
hsa-miR-892b	1138-1144	7mer-1A	-0.22	89	-0.22	0.048	N/A
hsa-miR-193a-3p	1138-1144	7mer-1A	-0.18	79	-0.18	2.032	< 0.1
hsa-miR-193b-3p	1138-1144	7mer-1A	-0.18	79	-0.18	2.032	< 0.1
hsa-miR-302a-5p	1165-1171	7mer-1A	-0.01	50	-0.01	0.245	N/A
hsa-miR-153-5p	1171-1178	8mer	-0.03	71	-0.03	0.048	N/A

hsa-miR-1250-3p	1172-1178	7mer-1A	-0.02	66	-0.02	0	N/A
hsa-miR-6847-3p	1175-1181	7mer-1A	-0.12	86	-0.12	0	N/A
hsa-miR-675-3p	1179-1185	7mer-m8	-0.21	92	-0.21	0	N/A
hsa-miR-3171	1181-1187	7mer-m8	-0.11	66	-0.11	0	N/A
hsa-miR-4426	1184-1190	7mer-1A	-0.03	77	-0.03	0	N/A
hsa-miR-4662b	1184-1190	7mer-1A	-0.08	77	-0.08	0	N/A
hsa-miR-4647	1184-1190	7mer-1A	-0.03	77	-0.03	0	N/A
hsa-miR-3658	1186-1192	7mer-1A	-0.05	79	-0.05	0	N/A
hsa-miR-3529-3p	1223-1229	7mer-m8	-0.02	29	-0.02	0	N/A
hsa-miR-549a	1225-1231	7mer-1A	-0.09	64	-0.07	0	N/A
hsa-miR-7-2-3p	1231-1238	8mer	-0.03	87	-0.02	0	N/A
hsa-miR-7-1-3p	1231-1238	8mer	-0.03	87	-0.02	0	N/A
hsa-miR-495-3p	1232-1238	7mer-1A	-0.01	63	-0.01	0.156	N/A
hsa-miR-5688	1232-1238	7mer-1A	-0.01	62	-0.01	0.156	N/A
hsa-miR-4796-3p	1249-1256	8mer	-0.08	86	-0.06	0	N/A
hsa-miR-3609	1250-1256	7mer-1A	-0.03	65	-0.02	0	N/A
hsa-miR-526b-3p	1250-1256	7mer-1A	-0.01	49	-0.01	0.156	< 0.1
hsa-miR-93-5p	1250-1256	7mer-1A	-0.01	47	-0.01	0.156	< 0.1
hsa-miR-20b-5p	1250-1256	7mer-1A	-0.01	47	-0.01	0.156	< 0.1
hsa-miR-17-5p	1250-1256	7mer-1A	-0.01	47	-0.01	0.156	< 0.1
hsa-miR-20a-5p	1250-1256	7mer-1A	-0.01	46	-0.01	0.156	< 0.1
hsa-miR-519d-3p	1250-1256	7mer-1A	-0.01	46	-0.01	0.156	< 0.1
hsa-miR-106b-5p	1250-1256	7mer-1A	-0.01	46	-0.01	0.156	< 0.1
hsa-miR-106a-5p	1250-1256	7mer-1A	-0.01	45	-0.01	0.156	< 0.1
hsa-miR-548ah-5p	1250-1256	7mer-1A	-0.01	39	-0.01	0	N/A
hsa-miR-4744	1252-1258	7mer-1A	-0.07	74	-0.06	0	N/A
hsa-miR-4509	1252-1258	7mer-1A	-0.05	64	-0.04	0	N/A
hsa-miR-548as-3p	1284-1290	7mer-m8	-0.09	87	-0.07	0	N/A
hsa-miR-8485	1290-1296	7mer-m8	-0.06	59	-0.05	0	N/A
hsa-miR-8485	1292-1298	7mer-m8	-0.02	19	-0.01	0	N/A
hsa-miR-8485	1294-1300	7mer-m8	-0.02	19	-0.01	0	N/A
hsa-miR-8485	1296-1302	7mer-m8	-0.06	60	-0.04	0	N/A
hsa-miR-8485	1298-1304	7mer-m8	-0.08	68	-0.05	0	N/A
hsa-miR-8485	1300-1306	7mer-m8	-0.1	72	-0.06	0	N/A
hsa-miR-8485	1302-1308	7mer-m8	-0.1	72	-0.06	0	N/A
hsa-miR-8485	1304-1310	7mer-m8	-0.09	71	-0.06	0	N/A
hsa-miR-8485	1306-1312	7mer-m8	-0.09	70	-0.06	0	N/A

hsa-miR-8485	1308-1314	7mer-m8	-0.09	70	-0.06	0	N/A
hsa-miR-8485	1310-1316	7mer-m8	-0.09	69	-0.06	0	N/A
hsa-miR-8485	1312-1318	7mer-m8	-0.09	70	-0.06	0	N/A
hsa-miR-8485	1314-1320	7mer-m8	-0.09	70	-0.06	0	N/A
hsa-miR-8485	1316-1322	7mer-m8	-0.09	71	-0.06	0	N/A
hsa-miR-8485	1318-1324	7mer-m8	-0.1	72	-0.06	0	N/A
hsa-miR-8485	1320-1326	7mer-m8	-0.1	73	-0.06	0.363	N/A
hsa-miR-8485	1322-1328	7mer-m8	-0.11	76	-0.07	0.363	N/A
hsa-miR-8485	1324-1330	7mer-m8	-0.15	84	-0.09	0.363	N/A
hsa-miR-5692a	1330-1336	7mer-1A	-0.01	56	-0.01	0	N/A
hsa-miR-568	1334-1341	8mer	-0.06	63	-0.04	0.734	N/A
hsa-miR-3149	1336-1342	7mer-1A	-0.01	28	-0.01	0.013	N/A
hsa-miR-7856-5p	1344-1350	7mer-1A	-0.01	40	-0.01	0	N/A
hsa-miR-3120-3p	1354-1360	7mer-m8	-0.08	76	-0.05	0	N/A
hsa-miR-1323	1359-1365	7mer-1A	-0.13	95	-0.08	0.013	N/A
hsa-miR-548o-3p	1359-1365	7mer-1A	-0.13	94	-0.08	0.013	N/A
hsa-miR-548aq-5p	1385-1392	8mer	-0.03	79	-0.02	0.054	N/A
hsa-miR-548as-5p	1385-1392	8mer	-0.03	67	-0.02	0.054	N/A
hsa-miR-559	1385-1392	8mer	-0.03	67	-0.02	0.054	N/A
hsa-miR-548ap-5p	1385-1392	8mer	-0.03	67	-0.02	0.054	N/A
hsa-miR-548j-5p	1385-1392	8mer	-0.03	66	-0.02	0.054	N/A
hsa-miR-548ak	1385-1392	8mer	-0.03	66	-0.02	0.054	N/A
hsa-miR-548h-5p	1385-1392	8mer	-0.03	66	-0.02	0.054	N/A
hsa-miR-548au-5p	1385-1392	8mer	-0.03	66	-0.02	0.054	N/A
hsa-miR-548w	1385-1392	8mer	-0.03	65	-0.02	0.054	N/A
hsa-miR-548ae-5p	1385-1392	8mer	-0.03	65	-0.02	0.054	N/A
hsa-miR-548ay-5p	1385-1392	8mer	-0.03	65	-0.02	0.054	N/A
hsa-miR-548am-5p	1385-1392	8mer	-0.03	65	-0.02	0.054	N/A
hsa-miR-548c-5p	1385-1392	8mer	-0.03	65	-0.02	0.054	N/A
hsa-miR-548ad-5p	1385-1392	8mer	-0.03	65	-0.02	0.054	N/A
hsa-miR-548b-5p	1385-1392	8mer	-0.03	65	-0.02	0.054	N/A
hsa-miR-548o-5p	1385-1392	8mer	-0.03	65	-0.02	0.054	N/A
hsa-miR-548d-5p	1385-1392	8mer	-0.03	65	-0.02	0.054	N/A
hsa-miR-548y	1385-1392	8mer	-0.03	64	-0.02	0.054	N/A
hsa-miR-548i	1385-1392	8mer	-0.03	64	-0.02	0.054	N/A
hsa-miR-548a-5p	1385-1392	8mer	-0.03	64	-0.02	0.054	N/A
hsa-miR-548bb-5p	1385-1392	8mer	-0.03	64	-0.02	0.054	N/A

hsa-miR-548ar-5p	1385-1392	8mer	-0.03	63	-0.02	0.054	N/A
hsa-miR-548ab	1385-1392	8mer	-0.03	63	-0.02	0.054	N/A
hsa-miR-548l	1386-1392	7mer-1A	-0.05	84	-0.03	0.013	N/A
hsa-miR-548k	1386-1392	7mer-1A	-0.03	76	-0.02	0.013	N/A
hsa-miR-548av-5p	1386-1392	7mer-1A	-0.02	67	-0.01	0.013	N/A
hsa-miR-8054	1386-1392	7mer-1A	-0.01	59	-0.01	0.013	N/A
hsa-miR-142-5p	1387-1393	7mer-m8	-0.03	73	-0.01	0.632	< 0.1
hsa-miR-5590-3p	1387-1393	7mer-m8	-0.03	71	-0.01	0.632	< 0.1
hsa-miR-548c-3p	1392-1398	7mer-1A	-0.01	57	0	0.013	N/A
hsa-miR-545-5p	1395-1401	7mer-m8	-0.05	61	-0.02	0	N/A
hsa-miR-4773	1405-1411	7mer-1A	-0.02	64	-0.01	0	N/A
hsa-miR-548n	1410-1416	7mer-m8	-0.02	53	-0.01	0.054	N/A
hsa-miR-3613-3p	1413-1420	8mer	-0.03	62	-0.01	0	N/A
hsa-miR-607	1416-1422	7mer-1A	-0.01	40	0	0	N/A
hsa-miR-222-5p	1427-1433	7mer-1A	-0.01	27	0	0	N/A
hsa-miR-1264	1432-1438	7mer-m8	-0.02	45	-0.01	0.013	N/A
hsa-miR-2115-3p	1437-1443	7mer-m8	-0.02	26	-0.01	0	N/A
hsa-miR-8063	1441-1447	7mer-m8	-0.02	77	-0.01	0	N/A
hsa-miR-676-5p	1449-1455	7mer-m8	-0.02	65	-0.01	0	N/A
hsa-miR-5088-3p	1452-1458	7mer-1A	-0.03	47	-0.01	0	N/A
hsa-miR-211-5p	1453-1459	7mer-1A	-0.04	63	-0.02	1.465	< 0.1
hsa-miR-204-5p	1453-1459	7mer-1A	-0.04	63	-0.02	1.465	< 0.1
hsa-miR-623	1453-1459	7mer-1A	-0.05	61	-0.02	0	N/A
hsa-miR-4755-5p	1453-1459	7mer-m8	-0.03	59	-0.01	0	N/A
hsa-miR-5006-3p	1453-1459	7mer-m8	-0.03	58	-0.01	0	N/A
hsa-miR-6734-3p	1455-1462	8mer	-0.2	89	-0.07	0	N/A
hsa-miR-3667-3p	1456-1462	7mer-1A	-0.05	59	-0.02	0	N/A
hsa-miR-6868-3p	1457-1463	7mer-1A	-0.01	24	0	0	N/A
hsa-miR-6875-3p	1466-1473	8mer	-0.05	71	-0.02	0	N/A
hsa-miR-4659b-3p	1467-1473	7mer-1A	-0.02	52	-0.01	0	N/A
hsa-miR-4659a-3p	1467-1473	7mer-1A	-0.02	52	-0.01	0	N/A
hsa-miR-4495	1479-1485	7mer-m8	-0.02	30	-0.01	0	N/A
hsa-miR-5692c	1490-1496	7mer-1A	-0.08	93	-0.03	0	N/A
hsa-miR-5692b	1490-1496	7mer-1A	-0.08	93	-0.03	0	N/A
hsa-miR-369-3p	1490-1496	7mer-1A	-0.01	53	0	0	N/A
hsa-miR-374a-5p	1490-1496	7mer-m8	-0.02	46	-0.01	0	N/A
hsa-miR-374b-5p	1490-1496	7mer-m8	-0.02	43	-0.01	0	N/A

hsa-miR-548c-3p	1505-1511	7mer-1A	-0.01	57	0	0	N/A
hsa-miR-3662	1515-1521	7mer-1A	-0.01	47	0	0	N/A
hsa-miR-324-5p	1530-1536	7mer-1A	-0.06	53	-0.02	0.414	N/A
hsa-miR-20a-3p	1532-1538	7mer-1A	-0.01	22	0	0.598	N/A
hsa-miR-544a	1533-1540	8mer	-0.03	37	-0.01	0.277	N/A
hsa-miR-6738-3p	1534-1540	7mer-1A	-0.01	32	0	0	N/A
hsa-miR-8061	1543-1549	7mer-1A	-0.03	60	-0.01	0	N/A
hsa-miR-4760-5p	1543-1549	7mer-1A	-0.02	52	-0.01	0	N/A
hsa-miR-153-5p	1547-1553	7mer-1A	-0.02	50	-0.01	0.048	N/A
hsa-miR-1250-3p	1547-1553	7mer-1A	-0.01	46	0	0	N/A
hsa-miR-616-3p	1549-1555	7mer-1A	-0.13	82	-0.05	0.013	N/A
hsa-miR-215-3p	1550-1556	7mer-m8	-0.24	96	-0.08	0.048	N/A
hsa-miR-6760-3p	1552-1559	8mer	-0.23	95	-0.08	0	N/A
hsa-miR-1208	1553-1559	7mer-1A	-0.05	70	-0.02	0.013	N/A
hsa-miR-4420	1554-1560	7mer-1A	-0.07	84	-0.03	0	N/A
hsa-miR-181b-3p	1554-1560	7mer-1A	-0.11	82	-0.04	0	N/A
hsa-miR-181b-2-3p	1554-1560	7mer-1A	-0.11	82	-0.04	0	N/A
hsa-miR-653-3p	1554-1560	7mer-1A	-0.06	74	-0.02	0.048	N/A
hsa-miR-4760-3p	1557-1563	7mer-1A	-0.01	40	0	0	N/A
hsa-miR-548t-3p	1564-1570	7mer-1A	-0.17	95	-0.06	0.048	N/A
hsa-miR-548aa	1564-1570	7mer-1A	-0.17	95	-0.06	0.048	N/A
hsa-miR-548ap-3p	1564-1570	7mer-1A	-0.17	95	-0.06	0.048	N/A
hsa-miR-548at-3p	1564-1570	7mer-1A	-0.09	91	-0.03	0.048	N/A
hsa-miR-548ay-3p	1564-1570	7mer-1A	-0.09	91	-0.03	0.048	N/A
hsa-miR-548as-3p	1564-1570	7mer-1A	-0.01	48	0	0	N/A
hsa-miR-4668-3p	1575-1581	7mer-1A	-0.01	56	0	0	N/A
hsa-miR-548c-3p	1582-1588	7mer-m8	-0.02	84	-0.01	0.013	N/A
hsa-miR-3613-3p	1585-1592	8mer	-0.03	62	-0.01	0	N/A
hsa-miR-607	1588-1594	7mer-1A	-0.01	40	0	0	N/A
hsa-miR-548c-3p	1600-1606	7mer-1A	-0.01	57	0	0.013	N/A
hsa-miR-524-5p	1607-1613	7mer-1A	-0.02	65	-0.01	0	N/A
hsa-miR-520d-5p	1607-1613	7mer-1A	-0.01	52	0	0	N/A
hsa-miR-924	1613-1619	7mer-m8	-0.11	78	-0.04	0.013	N/A
hsa-miR-4294	1614-1620	7mer-m8	-0.12	77	-0.04	0	N/A
hsa-miR-6858-5p	1617-1624	8mer	-0.08	77	-0.03	0	N/A
hsa-miR-4689	1617-1624	8mer	-0.11	76	-0.04	0	N/A
hsa-miR-3150b-3p	1617-1623	7mer-1A	-0.07	59	-0.02	0	N/A

hsa-miR-4784	1617-1623	7mer-1A	-0.07	57	-0.02	0	N/A
hsa-miR-1224-5p	1618-1624	7mer-1A	-0.01	48	0	0.891	N/A
hsa-miR-3915	1618-1624	7mer-1A	-0.01	14	0	0	N/A
hsa-miR-500b-5p	1622-1628	7mer-m8	-0.08	73	-0.03	1.746	N/A
hsa-miR-362-5p	1622-1628	7mer-m8	-0.08	72	-0.03	1.746	N/A
hsa-miR-500a-5p	1623-1629	7mer-m8	-0.02	47	-0.01	0.013	N/A
hsa-miR-300	1629-1635	7mer-1A	-0.01	51	0	0.332	N/A
hsa-miR-381-3p	1629-1635	7mer-1A	-0.01	48	0	0.332	N/A
hsa-miR-4666a-3p	1629-1635	7mer-1A	-0.01	46	0	0	N/A
hsa-let-7b-3p	1629-1635	7mer-m8	-0.02	36	-0.01	0	N/A
hsa-let-7f-1-3p	1629-1635	7mer-m8	-0.02	35	-0.01	0	N/A
hsa-let-7a-3p	1629-1635	7mer-m8	-0.02	35	-0.01	0	N/A
hsa-miR-98-3p	1629-1635	7mer-m8	-0.02	35	-0.01	0	N/A
hsa-miR-448	1633-1640	8mer	-0.16	89	-0.06	0.099	N/A
hsa-miR-153-3p	1634-1640	7mer-1A	-0.07	65	-0.02	0.099	< 0.1
hsa-miR-4684-3p	1636-1642	7mer-1A	-0.09	66	-0.03	0	N/A
hsa-miR-892a	1640-1647	8mer	-0.09	79	-0.03	0.013	N/A
hsa-miR-4693-5p	1641-1648	8mer	-0.11	72	-0.04	0	N/A
hsa-miR-4318	1641-1647	7mer-1A	-0.08	70	-0.03	0	N/A
hsa-miR-3156-5p	1651-1657	7mer-m8	-0.02	37	-0.01	0	N/A
hsa-miR-7-5p	1654-1660	7mer-1A	-0.03	66	-0.01	3.315	0.1
hsa-miR-6077	1654-1660	7mer-1A	-0.01	38	0	0	N/A
hsa-miR-4694-3p	1657-1663	7mer-m8	-0.02	42	-0.01	0	N/A
hsa-miR-3662	1659-1665	7mer-1A	-0.01	47	0	0	N/A
hsa-miR-196a-5p	1665-1671	7mer-m8	-0.2	88	-0.07	0.085	< 0.1
hsa-miR-196b-5p	1665-1671	7mer-m8	-0.19	87	-0.06	0.085	< 0.1
hsa-miR-548m	1667-1673	7mer-m8	-0.03	56	-0.01	0	N/A
hsa-miR-6730-5p	1668-1675	8mer	-0.13	89	-0.04	0	N/A
hsa-miR-488-3p	1669-1676	8mer	-0.05	73	-0.02	0.082	N/A
hsa-miR-5681a	1669-1675	7mer-1A	-0.08	71	-0.03	0	N/A
hsa-miR-3614-5p	1674-1680	7mer-1A	-0.06	64	-0.02	0	N/A
hsa-miR-6500-3p	1674-1680	7mer-1A	-0.01	26	0	0	N/A
hsa-miR-5096	1677-1683	7mer-1A	-0.01	44	0	0	N/A
hsa-miR-335-3p	1678-1684	7mer-1A	-0.01	40	0	0	N/A
hsa-miR-3149	1693-1699	7mer-m8	-0.05	73	-0.02	0.013	N/A
hsa-miR-297	1693-1699	7mer-1A	-0.08	66	-0.03	0.373	N/A
hsa-miR-675-3p	1693-1699	7mer-1A	-0.03	63	-0.01	0	N/A

hsa-miR-1279	1697-1703	7mer-m8	-0.02	57	-0.01	0	N/A
hsa-miR-551b-5p	1705-1711	7mer-m8	-0.02	62	-0.01	0	N/A
hsa-miR-4766-5p	1709-1715	7mer-1A	-0.09	90	-0.03	0	N/A
hsa-miR-4703-3p	1733-1739	7mer-1A	-0.15	80	-0.05	0	N/A
hsa-miR-7852-3p	1734-1741	8mer	-0.2	90	-0.07	0	N/A
hsa-miR-3668	1735-1741	7mer-1A	-0.11	70	-0.04	0	N/A
hsa-miR-7856-5p	1742-1748	7mer-1A	-0.01	40	0	0	N/A
hsa-miR-5003-3p	1745-1751	7mer-1A	-0.01	46	0	0	N/A
hsa-miR-548b-3p	1752-1759	8mer	-0.12	90	-0.04	0	N/A
hsa-miR-4511	1753-1759	7mer-1A	-0.01	43	0	0	N/A
hsa-miR-3161	1756-1763	8mer	-0.03	61	-0.01	0	N/A
hsa-miR-599	1757-1763	7mer-m8	-0.02	23	-0.01	0.052	N/A
hsa-miR-4780	1760-1766	7mer-m8	-0.17	82	-0.06	0	N/A
hsa-miR-1303	1767-1773	7mer-m8	-0.02	23	-0.01	0.013	N/A
hsa-miR-3908	1774-1780	7mer-m8	-0.12	92	-0.04	0	N/A
hsa-miR-548e-5p	1777-1783	7mer-m8	-0.02	39	-0.01	0	N/A
hsa-miR-130a-5p	1793-1799	7mer-1A	-0.01	26	0	0.642	< 0.1
hsa-miR-23b-3p	1793-1799	7mer-1A	-0.01	26	0	0.642	< 0.1
hsa-miR-23a-3p	1793-1799	7mer-1A	-0.01	26	0	0.642	< 0.1
hsa-miR-23c	1793-1799	7mer-1A	-0.01	25	0	0.642	< 0.1
hsa-miR-5000-5p	1796-1802	7mer-1A	-0.07	76	-0.03	0	N/A
hsa-miR-548at-5p	1801-1807	7mer-m8	-0.02	46	-0.01	0	N/A
hsa-miR-3653-3p	1807-1813	7mer-1A	-0.02	63	-0.01	0	N/A
hsa-miR-3658	1807-1814	8mer	-0.03	60	-0.01	0	N/A
hsa-miR-33a-3p	1812-1818	7mer-m8	-0.02	49	-0.01	0	N/A
hsa-miR-5582-3p	1840-1846	7mer-1A	-0.04	90	-0.01	0.27	N/A
hsa-miR-548a-3p	1840-1846	7mer-1A	-0.01	47	0	0.276	N/A
hsa-miR-548az-3p	1840-1846	7mer-1A	-0.01	46	0	0.276	N/A
hsa-miR-548ar-3p	1840-1846	7mer-1A	-0.01	46	0	0.276	N/A
hsa-miR-548e-3p	1840-1846	7mer-1A	-0.01	46	0	0.276	N/A
hsa-miR-548f-3p	1840-1846	7mer-1A	-0.01	45	0	0.276	N/A
hsa-miR-3163	1842-1848	7mer-1A	-0.01	45	0	0	N/A
hsa-miR-410-3p	1844-1850	7mer-1A	-0.01	55	0	0.16	N/A
hsa-miR-190a-3p	1845-1851	7mer-1A	-0.01	27	0	0.048	N/A
hsa-miR-6083	1846-1852	7mer-1A	-0.01	45	0	0	N/A
hsa-miR-4320	1852-1858	7mer-m8	-0.04	55	-0.01	0	N/A
hsa-miR-5089-5p	1854-1860	7mer-m8	-0.02	37	-0.01	0	N/A

hsa-miR-4769-5p	1855-1862	8mer	-0.2	94	-0.07	0.013	N/A
hsa-miR-4654	1855-1862	8mer	-0.25	94	-0.08	0.013	N/A
hsa-miR-4648	1856-1862	7mer-1A	-0.07	71	-0.03	0	N/A
hsa-miR-1233-5p	1856-1862	7mer-1A	-0.07	46	-0.02	0	N/A
hsa-miR-6778-5p	1856-1862	7mer-1A	-0.07	46	-0.02	0	N/A
hsa-miR-299-3p	1857-1863	7mer-m8	-0.17	84	-0.06	1.743	N/A
hsa-miR-4666a-5p	1860-1866	7mer-m8	-0.02	36	-0.01	0	N/A
hsa-miR-493-5p	1862-1868	7mer-m8	-0.07	76	-0.02	0.16	N/A
hsa-miR-548ac	1874-1880	7mer-1A	-0.01	57	0	0.297	N/A
hsa-miR-548z	1874-1880	7mer-1A	-0.01	57	0	0.297	N/A
hsa-miR-548bb-3p	1874-1880	7mer-1A	-0.01	57	0	0.297	N/A
hsa-miR-548d-3p	1874-1880	7mer-1A	-0.01	57	0	0.297	N/A
hsa-miR-548h-3p	1874-1880	7mer-1A	-0.01	57	0	0.297	N/A
hsa-miR-548am-3p	1874-1880	7mer-1A	-0.01	50	0	0.048	N/A
hsa-miR-548ae-3p	1874-1880	7mer-1A	-0.01	50	0	0.048	N/A
hsa-miR-548aq-3p	1874-1880	7mer-1A	-0.01	50	0	0.048	N/A
hsa-miR-548j-3p	1874-1880	7mer-1A	-0.01	50	0	0.048	N/A
hsa-miR-548ah-3p	1874-1880	7mer-1A	-0.01	50	0	0.048	N/A
hsa-miR-548x-3p	1874-1880	7mer-1A	-0.01	49	0	0.048	N/A
hsa-miR-548aj-3p	1874-1880	7mer-1A	-0.01	49	0	0.048	N/A
hsa-miR-4799-5p	1877-1883	7mer-1A	-0.01	35	0	0	N/A
hsa-miR-6802-3p	1885-1891	7mer-m8	-0.13	76	-0.04	0	N/A
hsa-miR-6879-3p	1886-1892	7mer-m8	-0.09	63	-0.03	0	N/A
hsa-miR-196a-5p	1891-1897	7mer-m8	-0.06	59	-0.02	1.639	< 0.1
hsa-miR-196b-5p	1891-1897	7mer-m8	-0.06	59	-0.02	1.639	< 0.1
hsa-miR-548ba	1893-1899	7mer-1A	-0.06	58	-0.02	0	N/A
hsa-miR-548ag	1893-1899	7mer-1A	-0.05	52	-0.02	0	N/A
hsa-miR-548ai	1893-1899	7mer-1A	-0.04	41	-0.01	0	N/A
hsa-miR-570-5p	1893-1899	7mer-1A	-0.04	41	-0.01	0	N/A
hsa-miR-5692b	1898-1904	7mer-1A	-0.07	91	-0.02	0	N/A
hsa-miR-5692c	1898-1904	7mer-1A	-0.07	91	-0.02	0	N/A
hsa-miR-374a-5p	1898-1905	8mer	-0.03	65	-0.01	0.695	N/A
hsa-miR-374b-5p	1898-1905	8mer	-0.03	64	-0.01	0.695	N/A
hsa-miR-369-3p	1898-1904	7mer-1A	-0.01	53	0	0.695	N/A
hsa-miR-590-3p	1903-1909	7mer-m8	-0.02	59	-0.01	0.549	N/A
hsa-miR-6505-5p	1907-1913	7mer-1A	-0.08	74	-0.03	0.048	N/A
hsa-miR-890	1909-1915	7mer-m8	-0.13	83	-0.04	0.054	N/A

hsa-miR-4261	1914-1920	7mer-1A	-0.07	62	-0.02	0	N/A
hsa-miR-3978	1915-1921	7mer-1A	-0.01	49	0	0	N/A
hsa-miR-432-5p	1917-1923	7mer-1A	-0.08	69	-0.03	0.054	N/A
hsa-miR-3184-3p	1922-1928	7mer-m8	-0.05	70	-0.02	0	N/A
hsa-miR-7109-3p	1938-1944	7mer-m8	-0.12	81	-0.04	0	N/A
hsa-miR-5582-3p	1949-1955	7mer-m8	-0.02	65	-0.01	0	N/A
hsa-miR-3148	1952-1958	7mer-1A	-0.06	80	-0.02	0	N/A
hsa-miR-6124	1952-1958	7mer-1A	-0.01	49	0	0	N/A
hsa-miR-3688-3p	1953-1959	7mer-m8	-0.09	80	-0.03	0	N/A
hsa-miR-4694-3p	1955-1961	7mer-m8	-0.03	63	-0.01	0	N/A
hsa-miR-4698	1957-1964	8mer	-0.03	75	-0.01	0	N/A
hsa-miR-8063	1958-1964	7mer-1A	-0.01	54	0	0	N/A
hsa-miR-511-3p	1972-1979	8mer	-0.09	72	-0.03	0.443	N/A
hsa-miR-200a-5p	1984-1990	7mer-1A	-0.04	74	-0.02	0	N/A
hsa-miR-200b-5p	1984-1990	7mer-1A	-0.01	46	0	0	N/A
hsa-miR-6823-5p	1990-1996	7mer-m8	-0.15	86	-0.05	0	N/A
hsa-miR-606	1996-2002	7mer-1A	-0.05	58	-0.02	0.013	N/A
hsa-let-7g-3p	2014-2021	8mer	-0.1	81	-0.04	0	N/A
hsa-let-7a-2-3p	2014-2021	8mer	-0.1	78	-0.03	0	N/A
hsa-let-7c-3p	2015-2021	7mer-1A	-0.04	59	-0.02	0.048	N/A
hsa-miR-493-5p	2015-2021	7mer-1A	-0.01	31	0	0.3	N/A
hsa-miR-643	2017-2023	7mer-1A	-0.08	70	-0.03	0.604	N/A
hsa-miR-578	2018-2025	8mer	-0.03	62	-0.01	0.048	N/A
hsa-miR-338-5p	2038-2044	7mer-1A	-0.01	35	0	0	N/A
hsa-miR-150-3p	2041-2047	7mer-m8	-0.15	70	-0.05	0	N/A
hsa-miR-582-3p	2044-2051	8mer	-0.22	94	-0.07	0	N/A
hsa-miR-1256	2051-2057	7mer-1A	-0.12	70	-0.04	0.276	N/A
hsa-miR-4642	2052-2058	7mer-1A	-0.11	79	-0.04	0	N/A
hsa-miR-182-5p	2053-2059	7mer-1A	-0.19	83	-0.06	2.753	0.11
hsa-miR-96-5p	2053-2059	7mer-1A	-0.11	77	-0.04	2.753	0.11
hsa-miR-1271-5p	2053-2059	7mer-1A	-0.12	77	-0.04	2.753	0.11
hsa-miR-6507-5p	2076-2082	7mer-m8	-0.02	59	-0.01	0	N/A
hsa-miR-3185	2078-2084	7mer-1A	-0.04	64	-0.01	0	N/A
hsa-miR-4762-3p	2082-2088	7mer-1A	-0.08	83	-0.03	0	N/A
hsa-miR-6780b-3p	2088-2094	7mer-m8	-0.23	93	-0.08	0	N/A
hsa-miR-7848-3p	2091-2097	7mer-1A	-0.12	73	-0.04	0	N/A
hsa-miR-6782-3p	2096-2102	7mer-1A	-0.05	68	-0.02	0	N/A

hsa-miR-6507-5p	2101-2108	8mer	-0.03	81	-0.01	0	N/A
hsa-miR-4263	2105-2111	7mer-m8	-0.02	67	-0.01	0	N/A
hsa-miR-5571-5p	2108-2114	7mer-1A	-0.01	53	0	0	N/A
hsa-miR-6505-5p	2129-2135	7mer-m8	-0.02	34	-0.01	0	N/A
hsa-miR-6128	2130-2136	7mer-m8	-0.1	87	-0.03	0.013	N/A
hsa-miR-1243	2132-2138	7mer-1A	-0.15	86	-0.05	0	N/A
hsa-miR-3654	2132-2138	7mer-1A	-0.05	76	-0.02	0	N/A
hsa-miR-1277-5p	2137-2143	7mer-1A	-0.03	74	-0.01	0	N/A
hsa-miR-889-3p	2138-2144	7mer-1A	-0.01	47	0	0.054	N/A
hsa-miR-568	2146-2153	8mer	-0.04	53	-0.01	0.054	N/A
hsa-miR-508-3p	2149-2155	7mer-m8	-0.16	82	-0.05	0.013	N/A
hsa-miR-5590-3p	2155-2161	7mer-1A	-0.07	87	-0.03	2.179	< 0.1
hsa-miR-340-5p	2155-2161	7mer-m8	-0.02	76	-0.01	2.179	N/A
hsa-miR-142-5p	2155-2161	7mer-1A	-0.01	44	0	2.179	< 0.1
hsa-miR-410-3p	2157-2163	7mer-1A	-0.01	55	0	1.957	N/A
hsa-miR-190a-3p	2158-2164	7mer-1A	-0.01	27	0	0	N/A
hsa-miR-6083	2159-2165	7mer-1A	-0.01	45	0	0	N/A
hsa-miR-4729	2161-2168	8mer	-0.03	74	-0.01	0	N/A
hsa-miR-5696	2162-2168	7mer-m8	-0.02	50	-0.01	0	N/A
hsa-miR-3148	2170-2177	8mer	-0.03	60	-0.01	0	N/A
hsa-miR-6124	2171-2177	7mer-1A	-0.01	49	0	0	N/A
hsa-miR-3688-3p	2172-2178	7mer-m8	-0.02	29	-0.01	0	N/A
hsa-miR-5591-3p	2177-2183	7mer-m8	-0.07	62	-0.02	0	N/A
hsa-miR-660-5p	2177-2183	7mer-1A	-0.06	60	-0.02	0.054	N/A
hsa-miR-3609	2200-2206	7mer-m8	-0.02	50	-0.01	0	N/A
hsa-miR-548ah-5p	2200-2206	7mer-m8	-0.02	48	-0.01	0	N/A
hsa-miR-548az-5p	2201-2207	7mer-m8	-0.02	64	-0.01	0	N/A
hsa-miR-548t-5p	2201-2207	7mer-m8	-0.02	64	-0.01	0	N/A
hsa-miR-3121-3p	2213-2219	7mer-1A	-0.01	35	0	0.013	N/A
hsa-miR-410-3p	2217-2223	7mer-1A	-0.01	55	0	1.936	N/A
hsa-miR-5011-5p	2218-2224	7mer-m8	-0.02	37	-0.01	0	N/A
hsa-miR-103a-3p	2223-2229	7mer-m8	-0.12	80	-0.04	1.219	< 0.1
hsa-miR-107	2223-2229	7mer-m8	-0.12	80	-0.04	1.219	< 0.1
hsa-miR-885-3p	2225-2231	7mer-1A	-0.19	89	-0.06	0.054	N/A
hsa-miR-6868-5p	2226-2232	7mer-m8	-0.21	90	-0.07	0	N/A
hsa-miR-892b	2228-2234	7mer-1A	-0.13	75	-0.05	0.048	N/A
hsa-miR-193a-3p	2228-2234	7mer-1A	-0.1	67	-0.04	1.285	< 0.1

hsa-miR-193b-3p	2228-2234	7mer-1A	-0.1	67	-0.04	1.285	< 0.1
hsa-miR-142-3p.2	2234-2240	7mer-m8	-0.05	61	-0.02	0.407	< 0.1
hsa-miR-4255	2234-2240	7mer-1A	-0.02	59	-0.01	0	N/A
hsa-miR-548p	2244-2250	7mer-1A	-0.13	93	-0.05	0.013	N/A
hsa-miR-587	2249-2255	7mer-m8	-0.02	39	-0.01	0.013	N/A
hsa-miR-1236-3p	2252-2258	7mer-m8	-0.06	66	-0.02	0.013	N/A
hsa-miR-3133	2260-2266	7mer-m8	-0.02	80	-0.01	0	N/A
hsa-miR-154-5p	2267-2273	7mer-1A	-0.08	73	-0.03	0.16	N/A
hsa-miR-708-3p	2279-2285	7mer-m8	-0.05	56	-0.02	0	N/A
hsa-miR-7844-5p	2280-2287	8mer	-0.03	74	-0.01	0	N/A
hsa-miR-30c-5p	2283-2289	7mer-1A	-0.01	9	0	0.041	< 0.1
hsa-miR-30e-5p	2283-2289	7mer-1A	-0.01	9	0	0.041	< 0.1
hsa-miR-30b-5p	2283-2289	7mer-1A	-0.01	9	0	0.041	< 0.1
hsa-miR-30d-5p	2283-2289	7mer-1A	-0.01	9	0	0.041	< 0.1
hsa-miR-30a-5p	2283-2289	7mer-1A	-0.01	9	0	0.041	< 0.1
hsa-miR-3607-3p	2284-2290	7mer-m8	-0.02	46	-0.01	0	N/A
hsa-miR-943	2287-2294	8mer	-0.21	93	-0.07	0.013	N/A
hsa-miR-3136-5p	2288-2294	7mer-1A	-0.15	83	-0.05	0.013	N/A
hsa-miR-4439	2288-2294	7mer-1A	-0.07	72	-0.02	0.013	N/A
hsa-miR-8070	2306-2312	7mer-1A	-0.01	33	-0.01	0	N/A
hsa-miR-4670-3p	2316-2322	7mer-m8	-0.09	73	-0.03	0	N/A
hsa-miR-4282	2331-2338	8mer	-0.03	87	-0.01	0	N/A
hsa-miR-3163	2333-2339	7mer-m8	-0.02	65	-0.01	0	N/A
hsa-miR-5692a	2336-2342	7mer-1A	-0.01	56	0	0	N/A
hsa-miR-3674	2348-2354	7mer-1A	-0.07	50	-0.02	0	N/A
hsa-miR-618	2355-2361	7mer-1A	-0.11	84	-0.04	0.013	N/A
hsa-miR-4639-5p	2359-2365	7mer-1A	-0.04	58	-0.01	0	N/A
hsa-miR-4678	2365-2372	8mer	-0.12	79	-0.04	0	N/A
hsa-miR-331-5p	2366-2372	7mer-m8	-0.06	52	-0.02	0.389	N/A
hsa-miR-1183	2370-2376	7mer-m8	-0.09	76	-0.03	0.054	N/A
hsa-miR-6822-3p	2387-2394	8mer	-0.17	83	-0.06	0	N/A
hsa-miR-8057	2388-2395	8mer	-0.11	90	-0.04	0	N/A
hsa-miR-760	2388-2394	7mer-1A	-0.1	62	-0.03	0.588	N/A
hsa-miR-3064-3p	2409-2415	7mer-m8	-0.03	33	-0.01	0	N/A
hsa-miR-365b-3p	2412-2418	7mer-1A	-0.01	30	0	0.654	< 0.1
hsa-miR-365a-3p	2412-2418	7mer-1A	-0.01	30	0	0.654	< 0.1
hsa-miR-15a-5p	2414-2425	non-canonical	N/A	N/A	N/A	0	N/A

hsa-miR-15a-5p	2414-2425	non-canonical	N/A	N/A	N/A	0	N/A
hsa-miR-3663-3p	2420-2426	7mer-1A	-0.04	44	-0.01	0	N/A
hsa-miR-3609	2424-2431	8mer	-0.03	66	-0.01	0	N/A
hsa-miR-548ah-5p	2424-2431	8mer	-0.03	64	-0.01	0	N/A
hsa-miR-4796-3p	2425-2431	7mer-1A	-0.01	52	0	0	N/A
hsa-miR-526b-3p	2425-2431	7mer-1A	-0.01	49	0	0.237	< 0.1
hsa-miR-17-5p	2425-2431	7mer-1A	-0.01	47	0	0.237	< 0.1
hsa-miR-20b-5p	2425-2431	7mer-1A	-0.01	47	0	0.237	< 0.1
hsa-miR-93-5p	2425-2431	7mer-1A	-0.01	47	0	0.237	< 0.1
hsa-miR-20a-5p	2425-2431	7mer-1A	-0.01	46	0	0.237	< 0.1
hsa-miR-106b-5p	2425-2431	7mer-1A	-0.01	46	0	0.237	< 0.1
hsa-miR-519d-3p	2425-2431	7mer-1A	-0.01	46	0	0.237	< 0.1
hsa-miR-106a-5p	2425-2431	7mer-1A	-0.01	45	0	0.237	< 0.1
hsa-miR-142-5p	2426-2432	7mer-m8	-0.02	59	-0.01	0.237	< 0.1
hsa-miR-5590-3p	2426-2432	7mer-m8	-0.02	56	-0.01	0.237	< 0.1
hsa-miR-95-5p	2428-2435	8mer	-0.03	47	-0.01	0.048	N/A
hsa-miR-7158-5p	2431-2437	7mer-m8	-0.02	52	-0.01	0	N/A
hsa-miR-4803	2439-2445	7mer-1A	-0.01	30	0	0.048	N/A
hsa-miR-302c-5p	2440-2446	7mer-m8	-0.02	58	-0.01	0	N/A
hsa-miR-3143	2440-2446	7mer-1A	-0.01	28	0	0	N/A
hsa-miR-3133	2448-2454	7mer-1A	-0.01	63	0	0	N/A
hsa-miR-186-5p	2448-2454	7mer-1A	-0.01	59	0	0.105	N/A
hsa-miR-4744	2449-2455	7mer-m8	-0.02	30	-0.01	0	N/A
hsa-miR-939-5p	2459-2465	7mer-m8	-0.24	92	-0.08	0	N/A
hsa-miR-1343-5p	2459-2465	7mer-m8	-0.2	90	-0.07	0	N/A
hsa-miR-4723-5p	2460-2466	7mer-m8	-0.22	72	-0.07	0	N/A
hsa-miR-6870-5p	2460-2466	7mer-m8	-0.19	68	-0.07	0	N/A
hsa-miR-5698	2460-2466	7mer-m8	-0.19	68	-0.07	0	N/A
hsa-miR-7111-5p	2460-2466	7mer-m8	-0.19	67	-0.07	0	N/A
hsa-miR-6794-5p	2461-2468	8mer	-0.27	88	-0.09	0.048	N/A
hsa-miR-4716-3p	2461-2468	8mer	-0.27	87	-0.09	0.048	N/A
hsa-miR-4278	2462-2469	8mer	-0.39	95	-0.13	0	N/A
hsa-miR-6732-5p	2462-2468	7mer-1A	-0.17	80	-0.06	0	N/A
hsa-miR-6805-5p	2462-2468	7mer-1A	-0.1	65	-0.03	0	N/A
hsa-miR-6824-5p	2463-2469	7mer-1A	-0.1	57	-0.04	0	N/A
hsa-miR-6789-5p	2463-2469	7mer-1A	-0.02	27	-0.01	0	N/A
hsa-miR-2909	2464-2470	7mer-1A	-0.01	34	0	0	N/A

hsa-miR-32-3p	2466-2472	7mer-m8	-0.02	73	-0.01	0	N/A
hsa-miR-205-3p	2472-2478	7mer-1A	-0.01	50	0	0	N/A
hsa-miR-138-2-3p	2473-2479	7mer-m8	-0.02	73	-0.01	0	N/A
hsa-miR-34c-3p	2477-2483	7mer-m8	-0.09	81	-0.03	0.054	N/A
hsa-miR-499b-3p	2479-2486	8mer	-0.21	94	-0.07	0.054	N/A
hsa-miR-499a-3p	2479-2486	8mer	-0.21	94	-0.07	0.054	N/A
hsa-miR-4699-3p	2486-2493	8mer	-0.03	67	-0.01	0	N/A
hsa-miR-4427	2492-2499	8mer	-0.17	94	-0.06	0	N/A
hsa-miR-5187-3p	2493-2499	7mer-1A	-0.01	41	0	0	N/A
hsa-miR-4680-3p	2493-2499	7mer-1A	-0.01	30	0	0	N/A
hsa-miR-548f-5p	2509-2516	8mer	-0.06	80	-0.02	0	N/A
hsa-miR-548x-5p	2509-2516	8mer	-0.05	77	-0.02	0	N/A
hsa-miR-548aj-5p	2509-2516	8mer	-0.05	77	-0.02	0	N/A
hsa-miR-548g-5p	2509-2516	8mer	-0.05	77	-0.02	0	N/A
hsa-miR-1468-3p	2510-2516	7mer-1A	-0.08	81	-0.03	0	N/A
hsa-miR-548aw	2510-2516	7mer-m8	-0.02	49	-0.01	0	N/A
hsa-miR-548c-3p	2516-2522	7mer-1A	-0.01	57	0	0.013	N/A
hsa-miR-331-5p	2521-2527	7mer-1A	-0.08	61	-0.03	0.048	N/A
hsa-miR-6509-5p	2521-2527	7mer-m8	-0.04	30	-0.01	0	N/A
hsa-miR-487b-5p	2525-2531	7mer-1A	-0.01	34	0	0	N/A
hsa-miR-487a-5p	2525-2531	7mer-1A	-0.01	33	0	0	N/A
hsa-miR-3189-3p	2528-2534	7mer-1A	-0.06	62	-0.02	0	N/A
hsa-miR-212-5p	2528-2534	7mer-1A	-0.02	48	-0.01	1.371	< 0.1
hsa-miR-502-5p	2529-2535	7mer-1A	-0.01	35	0	0.054	N/A
hsa-miR-1538	2537-2543	7mer-1A	-0.23	68	-0.08	0	N/A
hsa-miR-4745-3p	2537-2543	7mer-1A	-0.2	63	-0.07	0	N/A
hsa-miR-663b	2538-2545	8mer	-0.18	89	-0.06	0.013	N/A
hsa-miR-7108-5p	2539-2545	7mer-m8	-0.07	74	-0.02	0	N/A
hsa-miR-644a	2540-2547	8mer	-0.19	86	-0.06	0.048	N/A
hsa-miR-147a	2541-2547	7mer-m8	-0.02	63	-0.01	0.054	N/A
hsa-miR-3911	2541-2547	7mer-1A	-0.07	55	-0.02	0	N/A
hsa-miR-6867-5p	2542-2548	7mer-m8	-0.02	16	-0.01	0	N/A
hsa-miR-3650	2543-2549	7mer-m8	-0.02	37	-0.01	0	N/A
hsa-miR-4761-5p	2546-2552	7mer-m8	-0.02	33	-0.01	0	N/A
hsa-miR-497-3p	2551-2557	7mer-1A	-0.01	55	0	0.598	N/A
hsa-miR-5003-5p	2562-2569	8mer	-0.04	44	-0.01	0	N/A
hsa-miR-548ag	2574-2580	7mer-m8	-0.02	20	-0.01	0	N/A

hsa-miR-548ba	2574-2580	7mer-m8	-0.02	19	-0.01	0	N/A
hsa-miR-548ai	2574-2580	7mer-m8	-0.02	19	-0.01	0	N/A
hsa-miR-570-5p	2574-2580	7mer-m8	-0.02	19	-0.01	0	N/A
hsa-miR-4533	2577-2583	7mer-1A	-0.08	71	-0.03	0	N/A
hsa-miR-3202	2577-2583	7mer-1A	-0.06	61	-0.02	0	N/A
hsa-miR-548ar-3p	2582-2588	7mer-m8	-0.02	61	-0.01	0.013	N/A
hsa-miR-548a-3p	2582-2588	7mer-m8	-0.02	60	-0.01	0.013	N/A
hsa-miR-548e-3p	2582-2588	7mer-m8	-0.02	59	-0.01	0.013	N/A
hsa-miR-548az-3p	2582-2588	7mer-m8	-0.02	59	-0.01	0.013	N/A
hsa-miR-548f-3p	2582-2588	7mer-m8	-0.02	59	-0.01	0.013	N/A
hsa-miR-548am-3p	2583-2590	8mer	-0.03	84	-0.01	0.048	N/A
hsa-miR-548j-3p	2583-2590	8mer	-0.03	84	-0.01	0.048	N/A
hsa-miR-548x-3p	2583-2590	8mer	-0.03	84	-0.01	0.048	N/A
hsa-miR-548aj-3p	2583-2590	8mer	-0.03	84	-0.01	0.048	N/A
hsa-miR-548ah-3p	2583-2590	8mer	-0.03	84	-0.01	0.048	N/A
hsa-miR-548aq-3p	2583-2590	8mer	-0.03	84	-0.01	0.048	N/A
hsa-miR-548ae-3p	2583-2590	8mer	-0.03	84	-0.01	0.048	N/A
hsa-miR-548d-3p	2584-2590	7mer-1A	-0.01	57	0	0.048	N/A
hsa-miR-548bb-3p	2584-2590	7mer-1A	-0.01	57	0	0.048	N/A
hsa-miR-548z	2584-2590	7mer-1A	-0.01	57	0	0.048	N/A
hsa-miR-548h-3p	2584-2590	7mer-1A	-0.01	57	0	0.048	N/A
hsa-miR-548ac	2584-2590	7mer-1A	-0.01	57	0	0.048	N/A
hsa-miR-302a-5p	2587-2593	7mer-1A	-0.01	50	0	0	N/A
hsa-miR-6828-5p	2596-2602	7mer-m8	-0.17	84	-0.06	0.048	N/A
hsa-miR-4476	2598-2604	7mer-1A	-0.05	68	-0.02	0	N/A
hsa-miR-6876-5p	2598-2604	7mer-1A	-0.01	42	0	0	N/A
hsa-miR-8065	2599-2606	8mer	-0.13	64	-0.05	0	N/A
hsa-miR-135b-3p	2601-2607	7mer-1A	-0.15	70	-0.05	0.048	N/A
hsa-miR-2277-3p	2610-2616	7mer-1A	-0.12	81	-0.04	0	N/A
hsa-miR-563	2613-2620	8mer	-0.43	97	-0.14	0	N/A
hsa-miR-380-5p	2613-2620	8mer	-0.39	97	-0.13	0	N/A
hsa-miR-1273g-5p	2615-2621	7mer-m8	-0.14	85	-0.05	0	N/A
hsa-miR-140-5p	2616-2622	7mer-m8	-0.06	64	-0.02	0.105	< 0.1
hsa-miR-548u	2628-2634	7mer-1A	-0.03	62	-0.01	0	N/A
hsa-miR-7161-5p	2628-2634	7mer-1A	-0.01	35	0	0	N/A
hsa-miR-6853-3p	2634-2641	8mer	-0.08	69	-0.03	0	N/A
hsa-miR-1206	2635-2641	7mer-1A	-0.03	37	-0.01	0.013	N/A

hsa-miR-124-5p	2636-2642	7mer-m8	-0.17	76	-0.06	0	N/A
hsa-miR-4255	2637-2643	7mer-m8	-0.13	87	-0.05	0	N/A
hsa-miR-4709-5p	2641-2647	7mer-m8	-0.08	81	-0.03	0	N/A
hsa-miR-3529-3p	2643-2649	7mer-1A	-0.03	51	-0.01	0	N/A
hsa-miR-2392	2652-2659	8mer	-0.18	88	-0.04	0.013	N/A
hsa-miR-1265	2653-2659	7mer-1A	-0.11	83	-0.02	0.013	N/A
hsa-miR-651-5p	2654-2660	7mer-m8	-0.08	78	-0.02	0.013	N/A
hsa-miR-222-5p	2667-2673	7mer-1A	-0.07	65	-0.02	0	N/A
hsa-miR-30d-3p	2668-2674	7mer-m8	-0.04	73	-0.01	0	N/A
hsa-miR-30a-3p	2668-2674	7mer-m8	-0.04	73	-0.01	0	N/A
hsa-miR-30e-3p	2668-2674	7mer-m8	-0.04	73	-0.01	0	N/A
hsa-miR-205-3p	2669-2675	7mer-m8	-0.02	69	0	0	N/A
hsa-miR-5692a	2674-2680	7mer-m8	-0.02	73	0	0	N/A
hsa-miR-539-5p	2677-2684	8mer	-0.03	80	-0.01	0.054	N/A
hsa-miR-433-3p	2682-2688	7mer-1A	-0.01	29	0	0.105	N/A
hsa-miR-7154-5p	2682-2688	7mer-1A	-0.02	26	0	0	N/A
hsa-miR-3123	2692-2698	7mer-1A	-0.03	69	-0.01	0	N/A
hsa-miR-3925-5p	2692-2698	7mer-1A	-0.01	54	0	0	N/A
hsa-miR-1303	2693-2699	7mer-m8	-0.08	65	-0.02	0.013	N/A
hsa-miR-5680	2701-2707	7mer-1A	-0.01	53	0	0	N/A
hsa-miR-3123	2716-2722	7mer-1A	-0.02	67	-0.01	0	N/A
hsa-miR-3925-5p	2716-2722	7mer-1A	-0.01	54	0	0	N/A
hsa-miR-1303	2717-2723	7mer-m8	-0.05	53	-0.01	0.013	N/A
hsa-miR-4313	2727-2733	7mer-1A	-0.22	85	-0.04	0	N/A
hsa-miR-4633-5p	2730-2736	7mer-1A	-0.13	68	-0.03	0	N/A
hsa-miR-323a-3p	2734-2740	7mer-1A	-0.01	52	0	0.013	N/A
hsa-miR-545-3p	2752-2758	7mer-m8	-0.04	56	-0.01	0.054	N/A
hsa-miR-3120-3p	2753-2760	8mer	-0.07	72	-0.01	0	N/A
hsa-let-7f-2-3p	2756-2762	7mer-m8	-0.02	54	0	0	N/A
hsa-miR-1185-2-3p	2756-2762	7mer-m8	-0.02	52	0	0	N/A
hsa-miR-1185-1-3p	2756-2762	7mer-m8	-0.02	51	0	0	N/A
hsa-miR-1277-5p	2759-2765	7mer-1A	-0.01	40	0	0	N/A
hsa-miR-576-5p	2762-2768	7mer-m8	-0.02	61	0	0	N/A
hsa-miR-892c-3p	2777-2783	7mer-1A	-0.09	78	-0.02	0.953	N/A
hsa-miR-4676-3p	2777-2783	7mer-1A	-0.06	72	-0.01	0.953	N/A
hsa-miR-452-5p	2777-2783	7mer-1A	-0.06	68	-0.01	0.953	N/A
hsa-miR-4693-5p	2778-2784	7mer-1A	-0.12	75	-0.03	0	N/A

hsa-miR-628-5p	2785-2791	7mer-m8	-0.21	93	-0.04	0.271	N/A
hsa-miR-93-3p	2786-2792	7mer-m8	-0.06	63	-0.01	0.048	N/A
hsa-miR-6893-3p	2787-2794	8mer	-0.24	93	-0.05	0.653	N/A
hsa-miR-370-3p	2787-2794	8mer	-0.14	89	-0.03	0.653	N/A
hsa-miR-500a-5p	2791-2797	7mer-1A	-0.01	33	0	0.054	N/A
hsa-miR-1261	2795-2802	8mer	-0.1	67	-0.02	0	N/A
hsa-miR-5004-3p	2797-2803	7mer-1A	-0.08	70	-0.02	0	N/A
hsa-miR-6740-5p	2799-2805	7mer-1A	-0.07	53	-0.01	0	N/A
hsa-miR-548aa	2814-2820	7mer-1A	-0.16	95	-0.03	0.048	N/A
hsa-miR-548t-3p	2814-2820	7mer-1A	-0.16	95	-0.03	0.048	N/A
hsa-miR-548ap-3p	2814-2820	7mer-1A	-0.15	94	-0.03	0.048	N/A
hsa-miR-548at-3p	2814-2820	7mer-1A	-0.09	91	-0.02	0.048	N/A
hsa-miR-548ay-3p	2814-2820	7mer-1A	-0.03	75	-0.01	0.048	N/A
hsa-miR-548as-3p	2814-2820	7mer-1A	-0.01	48	0	0	N/A
hsa-miR-1285-5p	2821-2827	7mer-1A	-0.01	57	0	0	N/A
hsa-miR-590-5p	2825-2831	7mer-m8	-0.11	87	-0.02	0.83	< 0.1
hsa-miR-21-5p	2825-2831	7mer-m8	-0.07	67	-0.01	0.83	< 0.1
hsa-miR-551b-5p	2831-2838	8mer	-0.03	87	-0.01	0.048	N/A
hsa-miR-302a-5p	2835-2841	7mer-1A	-0.01	50	0	0.245	N/A
hsa-miR-1-5p	2854-2861	8mer	-0.34	98	-0.07	0.048	< 0.1
hsa-miR-539-3p	2856-2862	7mer-1A	-0.26	91	-0.05	0.054	N/A
hsa-miR-485-3p	2856-2862	7mer-1A	-0.13	84	-0.03	0.054	N/A
hsa-miR-664a-3p	2858-2864	7mer-1A	-0.01	33	0	0.054	N/A
hsa-miR-6074	2859-2865	7mer-m8	-0.02	45	0	0	N/A
hsa-miR-3145-3p	2861-2867	7mer-1A	-0.11	77	-0.02	0	N/A
hsa-miR-4762-3p	2864-2871	8mer	-0.23	97	-0.05	0	N/A
hsa-miR-3192-3p	2864-2870	7mer-1A	-0.03	63	-0.01	0	N/A
hsa-miR-16-1-3p	2870-2876	7mer-m8	-0.02	39	0	0.048	N/A
hsa-miR-144-3p	2871-2877	7mer-m8	-0.02	51	-0.01	0.099	< 0.1
hsa-miR-8485	2875-2881	7mer-m8	-0.02	19	0	0	N/A
hsa-miR-30d-5p	2879-2885	7mer-m8	-0.02	16	0	0.774	< 0.1
hsa-miR-30a-5p	2879-2885	7mer-m8	-0.02	16	0	0.774	< 0.1
hsa-miR-30b-5p	2879-2885	7mer-m8	-0.02	15	0	0.774	< 0.1
hsa-miR-30e-5p	2879-2885	7mer-m8	-0.02	15	0	0.774	< 0.1
hsa-miR-30c-5p	2879-2885	7mer-m8	-0.02	15	0	0.774	< 0.1
hsa-miR-4693-3p	2884-2890	7mer-1A	-0.22	87	-0.04	0	N/A
hsa-miR-6761-5p	2885-2892	8mer	-0.19	94	-0.04	0	N/A

hsa-miR-4251	2886-2892	7mer-1A	-0.1	82	-0.02	0	N/A
hsa-miR-4329	2886-2892	7mer-1A	-0.01	45	0	0	N/A
hsa-miR-3200-5p	2887-2893	7mer-m8	-0.2	94	-0.04	0	N/A
hsa-miR-4668-3p	2891-2897	7mer-1A	-0.01	56	0	0	N/A
hsa-miR-605-5p	2905-2911	7mer-1A	-0.01	44	0	0.013	N/A
hsa-miR-3145-3p	2911-2917	7mer-1A	-0.08	69	-0.02	0	N/A
hsa-miR-101-5p	2919-2926	8mer	-0.12	89	-0.02	0	N/A
hsa-miR-646	2926-2933	8mer	-0.19	92	-0.04	0.013	N/A
hsa-miR-4524a-5p	2927-2934	8mer	-0.24	89	-0.05	0	N/A
hsa-miR-4524b-5p	2927-2934	8mer	-0.24	89	-0.05	0	N/A
hsa-miR-503-5p	2927-2933	7mer-1A	-0.11	85	-0.02	1.206	< 0.1
hsa-miR-424-5p	2927-2933	7mer-1A	-0.13	77	-0.03	1.206	< 0.1
hsa-miR-497-5p	2927-2933	7mer-1A	-0.12	76	-0.02	1.206	< 0.1
hsa-miR-6838-5p	2927-2933	7mer-1A	-0.12	75	-0.03	1.206	< 0.1
hsa-miR-16-5p	2927-2933	7mer-1A	-0.11	74	-0.02	1.206	< 0.1
hsa-miR-15b-5p	2927-2933	7mer-1A	-0.11	73	-0.02	1.206	< 0.1
hsa-miR-15a-5p	2927-2933	7mer-1A	-0.11	73	-0.02	1.206	< 0.1
hsa-miR-195-5p	2927-2933	7mer-1A	-0.11	73	-0.02	1.206	< 0.1
hsa-miR-374b-3p	2928-2934	7mer-m8	-0.02	37	0	0	N/A
hsa-miR-580-3p	2945-2951	7mer-m8	-0.11	90	-0.02	0.013	N/A
hsa-miR-4677-3p	2948-2954	7mer-m8	-0.18	91	-0.04	0	N/A
hsa-miR-1208	2951-2957	7mer-1A	-0.03	59	-0.01	0.013	N/A
hsa-miR-6760-3p	2951-2957	7mer-1A	-0.01	39	0	0	N/A
hsa-miR-34c-3p	2953-2959	7mer-1A	-0.03	41	-0.01	0.013	N/A
hsa-miR-889-3p	2956-2962	7mer-m8	-0.02	76	0	0.013	N/A
hsa-miR-510-3p	2965-2971	7mer-1A	-0.01	43	0	0	N/A
hsa-miR-1250-3p	2969-2975	7mer-1A	-0.01	46	0	0	N/A
hsa-miR-153-5p	2969-2975	7mer-1A	-0.01	33	0	0	N/A
hsa-miR-616-3p	2971-2977	7mer-1A	-0.1	72	-0.02	0.013	N/A
hsa-miR-6733-3p	2974-2981	8mer	-0.33	97	-0.07	0	N/A
hsa-miR-4302	2975-2981	7mer-1A	-0.03	55	-0.01	0	N/A
hsa-miR-4797-3p	2977-2983	7mer-1A	-0.07	66	-0.01	0	N/A
hsa-miR-4264	2977-2983	7mer-1A	-0.04	63	-0.01	0	N/A
hsa-miR-6833-3p	2980-2986	7mer-m8	-0.02	61	0	0	N/A
hsa-miR-4768-5p	2980-2986	7mer-m8	-0.02	59	0	0	N/A
hsa-miR-6873-3p	2980-2986	7mer-1A	-0.01	20	0	0	N/A
hsa-miR-2117	2981-2987	7mer-m8	-0.03	31	-0.01	0	N/A

hsa-miR-4277	2983-2990	8mer	-0.03	65	-0.01	0	N/A
hsa-miR-584-3p	2984-2990	7mer-1A	-0.01	33	0	0	N/A
hsa-miR-30d-3p	2986-2993	8mer	-0.03	68	-0.01	0.013	N/A
hsa-miR-30a-3p	2986-2993	8mer	-0.03	68	-0.01	0.013	N/A
hsa-miR-30e-3p	2986-2993	8mer	-0.03	68	-0.01	0.013	N/A
hsa-miR-335-3p	2988-2994	7mer-1A	-0.01	40	0	0	N/A
hsa-miR-4703-3p	2993-2999	7mer-1A	-0.1	67	-0.02	0	N/A
hsa-miR-7852-3p	2994-3000	7mer-m8	-0.11	77	-0.02	0	N/A
hsa-miR-3171	2996-3002	7mer-1A	-0.06	42	-0.01	0	N/A
hsa-miR-3136-5p	3000-3007	8mer	-0.26	95	-0.05	0.013	N/A
hsa-miR-4439	3000-3007	8mer	-0.15	90	-0.03	0.013	N/A
hsa-miR-6855-3p	3000-3006	7mer-1A	-0.09	73	-0.02	0	N/A
hsa-miR-4513	3000-3006	7mer-1A	-0.08	70	-0.02	0	N/A
hsa-miR-6857-3p	3000-3006	7mer-1A	-0.08	61	-0.02	0	N/A
hsa-miR-943	3001-3007	7mer-1A	-0.11	80	-0.02	0.013	N/A
hsa-miR-4484	3030-3036	7mer-1A	-0.15	91	-0.03	0	N/A
hsa-miR-548an	3030-3036	7mer-1A	-0.12	81	-0.03	0	N/A
hsa-miR-6768-3p	3030-3036	7mer-1A	-0.05	70	-0.01	0	N/A
hsa-miR-548at-5p	3035-3041	7mer-m8	-0.03	63	-0.01	0	N/A
hsa-miR-4495	3041-3047	7mer-m8	-0.07	79	-0.02	0	N/A
hsa-miR-3920	3048-3055	8mer	-0.29	98	-0.06	0	N/A
hsa-miR-29a-5p	3049-3055	7mer-1A	-0.15	89	-0.03	0	N/A
hsa-miR-6507-5p	3055-3061	7mer-1A	-0.01	45	0	0	N/A
hsa-miR-20a-3p	3066-3073	8mer	-0.19	91	-0.04	0	N/A
hsa-miR-622	3070-3076	7mer-m8	-0.15	86	-0.03	0	N/A
hsa-let-7f-2-3p	3074-3080	7mer-m8	-0.06	77	-0.01	0	N/A
hsa-miR-1185-1-3p	3074-3080	7mer-m8	-0.04	69	-0.01	0	N/A
hsa-miR-1185-2-3p	3074-3080	7mer-m8	-0.04	69	-0.01	0	N/A
hsa-miR-331-5p	3078-3085	8mer	-0.3	95	-0.06	0	N/A
hsa-miR-4678	3078-3084	7mer-1A	-0.11	77	-0.02	0	N/A
hsa-miR-6509-5p	3079-3086	8mer	-0.29	95	-0.06	0	N/A
hsa-miR-889-3p	3086-3092	7mer-1A	-0.01	47	0	0.251	N/A
hsa-miR-559	3098-3104	7mer-m8	-0.07	87	-0.02	0.013	N/A
hsa-miR-548ak	3098-3104	7mer-m8	-0.07	86	-0.02	0.013	N/A
hsa-miR-548c-5p	3098-3104	7mer-m8	-0.07	86	-0.02	0.013	N/A
hsa-miR-548ay-5p	3098-3104	7mer-m8	-0.07	86	-0.02	0.013	N/A
hsa-miR-548o-5p	3098-3104	7mer-m8	-0.07	86	-0.02	0.013	N/A

hsa-miR-548ad-5p	3098-3104	7mer-m8	-0.07	86	-0.02	0.013	N/A
hsa-miR-548b-5p	3098-3104	7mer-m8	-0.07	86	-0.02	0.013	N/A
hsa-miR-548d-5p	3098-3104	7mer-m8	-0.07	86	-0.02	0.013	N/A
hsa-miR-548am-5p	3098-3104	7mer-m8	-0.07	86	-0.02	0.013	N/A
hsa-miR-548w	3098-3104	7mer-m8	-0.07	86	-0.02	0.013	N/A
hsa-miR-548ae-5p	3098-3104	7mer-m8	-0.07	86	-0.02	0.013	N/A
hsa-miR-548h-5p	3098-3104	7mer-m8	-0.07	86	-0.02	0.013	N/A
hsa-miR-548au-5p	3098-3104	7mer-m8	-0.07	86	-0.02	0.013	N/A
hsa-miR-548y	3098-3104	7mer-m8	-0.07	85	-0.02	0.013	N/A
hsa-miR-548i	3098-3104	7mer-m8	-0.07	85	-0.02	0.013	N/A
hsa-miR-548bb-5p	3098-3104	7mer-m8	-0.07	85	-0.02	0.013	N/A
hsa-miR-548ab	3098-3104	7mer-m8	-0.07	85	-0.02	0.013	N/A
hsa-miR-548ar-5p	3098-3104	7mer-m8	-0.07	85	-0.02	0.013	N/A
hsa-miR-548a-5p	3098-3104	7mer-m8	-0.06	82	-0.01	0.013	N/A
hsa-miR-548as-5p	3098-3104	7mer-m8	-0.05	80	-0.01	0.013	N/A
hsa-miR-548ap-5p	3098-3104	7mer-m8	-0.04	75	-0.01	0.013	N/A
hsa-miR-548j-5p	3098-3104	7mer-m8	-0.04	75	-0.01	0.013	N/A
hsa-miR-548aq-5p	3098-3104	7mer-m8	-0.02	53	0	0.013	N/A
hsa-miR-548n	3099-3106	8mer	-0.06	88	-0.01	0.013	N/A
hsa-miR-548t-5p	3100-3106	7mer-1A	-0.11	95	-0.02	0	N/A
hsa-miR-548az-5p	3100-3106	7mer-1A	-0.11	95	-0.02	0	N/A
hsa-miR-3163	3102-3108	7mer-1A	-0.01	45	0	0	N/A
hsa-miR-340-5p	3103-3109	7mer-1A	-0.01	51	0	0.594	N/A
hsa-miR-548ba	3112-3118	7mer-m8	-0.17	89	-0.04	0	N/A
hsa-miR-570-5p	3112-3118	7mer-m8	-0.14	84	-0.03	0	N/A
hsa-miR-548ai	3112-3118	7mer-m8	-0.14	84	-0.03	0	N/A
hsa-miR-548ag	3112-3118	7mer-m8	-0.13	82	-0.03	0	N/A
hsa-miR-548m	3113-3119	7mer-m8	-0.15	91	-0.03	0	N/A
hsa-miR-6083	3127-3133	7mer-1A	-0.01	45	0	0	N/A
hsa-miR-590-3p	3134-3140	7mer-m8	-0.04	90	-0.01	0.013	N/A
hsa-miR-3653-3p	3141-3147	7mer-m8	-0.07	82	-0.01	0	N/A
hsa-miR-552-5p	3148-3155	8mer	-0.21	98	0	0	N/A

Supplementary Table 8. 121 predicted miRNAs potentially binding with both circ-MALAT1 and JAK2

No.	miRNA	Total Score	Total Energy	Max Score	Max Energy	Len1	Len2	Positions
1	hsa-miR-6887-3p	323	-59.24	162	-30.5	21	867	351 538
2	hsa-miR-4773	457	-53.42	159	-17.98	22	867	458 622 648
3	hsa-miR-214-3p	285	-45.74	145	-27.71	22	867	628 453
4	hsa-miR-676-5p	296	-38.44	153	-22.23	21	867	739 779
5	hsa-miR-503-5p	172	-36.46	172	-36.46	23	867	656
6	hsa-miR-3529-3p	297	-34.81	152	-17.62	24	867	553 737
7	hsa-miR-605-5p	160	-33.77	160	-33.77	23	867	362
8	hsa-miR-1236-3p	144	-32.58	144	-32.58	22	867	539
9	hsa-miR-5088-3p	140	-31.98	140	-31.98	21	867	541
10	hsa-miR-6826-3p	162	-31.38	162	-31.38	22	867	350
11	hsa-miR-3686	323	-30.32	175	-15.47	22	867	578 385
12	hsa-miR-548at-5p	315	-30.1	158	-17.45	22	867	278 47
13	hsa-miR-6744-3p	171	-29.01	171	-29.01	23	867	124
14	hsa-miR-3184-3p	141	-28.94	141	-28.94	23	867	547
15	hsa-miR-7109-3p	152	-28.76	152	-28.76	22	867	548

16	hsa-miR-4699-3p	296	-28.31	150	-20.21	22	867	638 178
17	hsa-miR-6879-3p	143	-27.7	143	-27.7	21	867	355
18	hsa-miR-4468	156	-25.19	156	-25.19	18	867	460
19	hsa-miR-4757-5p	150	-25.02	150	-25.02	23	867	122
20	hsa-miR-20a-3p	284	-24.73	142	-12.47	22	867	595 638
21	hsa-miR-6802-3p	141	-24.71	141	-24.71	22	867	543
22	hsa-miR-885-3p	142	-23.77	142	-23.77	22	867	661
23	hsa-miR-3192-3p	152	-23.71	152	-23.71	21	867	177
24	hsa-miR-1182	141	-23.05	141	-23.05	23	867	125
25	hsa-miR-518a-5p	166	-23.01	166	-23.01	20	867	385
26	hsa-miR-527	166	-23.01	166	-23.01	20	867	385
27	hsa-miR-3667-3p	142	-22.84	142	-22.84	22	867	539
28	hsa-miR-5582-3p	284	-22.4	143	-11.66	22	867	267 507
29	hsa-miR-6876-5p	146	-21.42	146	-21.42	22	867	450
30	hsa-miR-8063	152	-20.99	152	-20.99	22	867	421
31	hsa-miR-607	156	-20.9	156	-20.9	21	867	28
32	hsa-miR-556-5p	140	-20.9	140	-20.9	22	867	489
33	hsa-miR-4709-5p	144	-20.73	144	-20.73	22	867	737
34	hsa-miR-6771-3p	150	-20.63	150	-20.63	21	867	364
35	hsa-miR-548ar-3p	171	-20.08	171	-20.08	21	867	795
36	hsa-miR-6734-3p	145	-20.05	145	-20.05	23	867	342
37	hsa-miR-4778-5p	152	-19.53	152	-19.53	22	867	580
38	hsa-miR-6830-3p	154	-19.39	154	-19.39	23	867	61
39	hsa-miR-548ba	144	-19.27	144	-19.27	22	867	602
40	hsa-miR-1226-3p	151	-19.23	151	-19.23	22	867	838
41	hsa-miR-6077	149	-18.71	149	-18.71	21	867	447
42	hsa-miR-6760-3p	146	-18.58	146	-18.58	21	867	232
43	hsa-miR-4635	147	-18.23	147	-18.23	21	867	612
44	hsa-miR-3120-3p	141	-17.62	141	-17.62	21	867	836
45	hsa-miR-1270	155	-17.57	155	-17.57	23	867	262
46	hsa-miR-561-3p	286	-17.31	146	-10.08	22	867	851 1
47	hsa-miR-4776-3p	148	-16.94	148	-16.94	23	867	112
48	hsa-miR-4789-5p	144	-16.48	144	-16.48	22	867	842
49	hsa-miR-3202	143	-16.47	143	-16.47	22	867	447
50	hsa-miR-508-3p	172	-16.11	172	-16.11	23	867	294
51	hsa-miR-34c-3p	143	-16.08	143	-16.08	22	867	730
52	hsa-miR-3606-3p	161	-16.01	161	-16.01	21	867	786

53	hsa-miR-548ai	143	-15.99	143	-15.99	22	867	602
54	hsa-miR-570-5p	143	-15.99	143	-15.99	22	867	602
55	hsa-miR-548g-3p	141	-15.82	141	-15.82	22	867	196
56	hsa-miR-320b	140	-15.51	140	-15.51	22	867	563
57	hsa-miR-548as-3p	147	-15.37	147	-15.37	22	867	364
58	hsa-miR-6875-3p	148	-15.25	148	-15.25	22	867	37
59	hsa-miR-320a	140	-15.14	140	-15.14	22	867	563
60	hsa-miR-511-5p	158	-15.13	158	-15.13	21	867	62
61	hsa-miR-548p	152	-15.09	152	-15.09	22	867	832
62	hsa-miR-4762-3p	148	-15.02	148	-15.02	22	867	177
63	hsa-miR-4670-3p	140	-14.85	140	-14.85	22	867	49
64	hsa-miR-539-5p	142	-14.73	142	-14.73	22	867	788
65	hsa-miR-3148	159	-14.6	159	-14.6	22	867	512
66	hsa-miR-3915	149	-14.5	149	-14.5	22	867	75
67	hsa-miR-6507-5p	154	-14.49	154	-14.49	21	867	75
68	hsa-miR-7154-5p	145	-14.38	145	-14.38	23	867	403
69	hsa-miR-1323	142	-14.37	142	-14.37	22	867	796
70	hsa-miR-549a	140	-14.11	140	-14.11	21	867	601
71	hsa-miR-5584-5p	140	-14.03	140	-14.03	22	867	263
72	hsa-miR-548ah-3p	148	-13.9	148	-13.9	22	867	795
73	hsa-miR-548o-3p	148	-13.66	148	-13.66	22	867	795
74	hsa-miR-545-3p	154	-13.63	154	-13.63	22	867	836
75	hsa-miR-548am-3p	148	-13.49	148	-13.49	22	867	795
76	hsa-miR-4524a-5p	146	-13.22	146	-13.22	22	867	663
77	hsa-miR-154-5p	151	-13.18	151	-13.18	22	867	151
78	hsa-miR-3613-3p	158	-13.04	158	-13.04	24	867	568
79	hsa-miR-548l	146	-12.87	146	-12.87	22	867	248
80	hsa-miR-4255	151	-12.86	151	-12.86	17	867	5
81	hsa-miR-8060	145	-12.12	145	-12.12	24	867	402
82	hsa-miR-485-3p	145	-11.98	145	-11.98	22	867	470
83	hsa-miR-6128	146	-11.95	146	-11.95	19	867	108
84	hsa-miR-4678	142	-11.94	142	-11.94	22	867	250
85	hsa-miR-4282	286	-11.75	146	-9.68	18	867	71 511
86	hsa-miR-4275	142	-11.62	142	-11.62	17	867	738
87	hsa-miR-548ae-3p	142	-11.56	142	-11.56	21	867	793
88	hsa-miR-539-3p	143	-11.44	143	-11.44	22	867	470
89	hsa-miR-651-3p	153	-11.4	153	-11.4	22	867	381

90	hsa-miR-32-3p	142	-11.27	142	-11.27	22	867	504
91	hsa-miR-4524b-5p	140	-11.24	140	-11.24	21	867	664
92	hsa-miR-4760-3p	140	-11.21	140	-11.21	22	867	436
93	hsa-miR-944	151	-11.13	151	-11.13	22	867	101
94	hsa-miR-135b-5p	142	-11.04	142	-11.04	23	867	411
95	hsa-miR-548x-3p	141	-11.02	141	-11.02	20	867	794
96	hsa-miR-548aj-3p	142	-10.74	142	-10.74	21	867	793
97	hsa-miR-548aq-3p	142	-10.74	142	-10.74	22	867	792
98	hsa-miR-4719	142	-10.58	142	-10.58	22	867	814
99	hsa-miR-135a-5p	143	-10.43	143	-10.43	23	867	409
100	hsa-miR-3163	288	-10.25	148	-6.63	22	867	201 71
101	hsa-miR-548j-3p	147	-10.15	147	-10.15	21	867	796
102	hsa-miR-4698	150	-10.04	150	-10.04	23	867	831
103	hsa-miR-372-5p	148	-9.98	148	-9.98	23	867	23
104	hsa-miR-4668-3p	155	-9.95	155	-9.95	23	867	419
105	hsa-miR-511-3p	141	-9.84	141	-9.84	20	867	197
106	hsa-miR-548az-3p	155	-9.83	155	-9.83	21	867	795
107	hsa-miR-513a-3p	142	-9.67	142	-9.67	23	867	782
108	hsa-miR-513c-3p	142	-9.67	142	-9.67	23	867	782
109	hsa-miR-633	140	-9.57	140	-9.57	23	867	663
110	hsa-miR-302c-5p	146	-9.28	146	-9.28	22	867	277
111	hsa-miR-1468-3p	140	-9.05	140	-9.05	22	867	833
112	hsa-miR-2115-3p	141	-9.04	141	-9.04	22	867	653
113	hsa-miR-548f-3p	152	-9.01	152	-9.01	19	867	794
114	hsa-miR-3646	141	-8.9	141	-8.9	22	867	202
115	hsa-miR-548e-3p	151	-8.6	151	-8.6	22	867	795
116	hsa-miR-548a-3p	158	-8.27	158	-8.27	22	867	792
117	hsa-miR-576-5p	140	-7.78	140	-7.78	22	867	669
118	hsa-miR-126-5p	141	-6.57	141	-6.57	21	867	99
119	hsa-miR-3607-3p	140	-5.96	140	-5.96	20	867	579
120	hsa-miR-8066	140	-5.88	140	-5.88	21	867	259
121	hsa-miR-548n	143	-5.55	143	-5.55	22	867	251

Supplementary Table 9. Primers for qRT-PCR

name	sequence（5'→3'）
U1 snRNA-F	GGGAGATACCATGATCACGAAGGT
U1 snRNA-R	CCACAAATTATGCAGTCGAGTTTCCC
18s rRNA-F	AACCCGTTGAACCCCATT
18s rRNA-R	CCATCCAATCGGTAGTAGCG
MALAT1-rt-F	GGTAACGATGGTGTCGAGGTC
MALAT1-rt-R	CCAGCATTACAGTTCTTGAACATG
pre-circ-MALAT1-F	GGTAACGATGGTGTCGAGGTC
pre-circ-MALAT1-R	CCAGCATTACAGTTCTTGAACATG
Oct4-rt-F	GGGAGATTGATAACTGGTGTGTT
Oct4-rt-R	GTGTATATCCCAGGGTGATCCTC
Sox2-rt-F	TACAGCATGTCCTACTCGCAG
Sox2-rt-R	GAGGAAGAGGTAACCACAGGG
NANOG-rt-F	TGATTTGTGGGCCTGAAGAAAA
NANOG-rt-R	GAGGCATCTCAGCAGAAGACA
β-actin-rt-F	CATGTACGTTGCTATCCAGGC
β-actin-rt-R	CTCCTTAATGTCACGCACGAT

GAPDH-rt-F	ATTGCCCTCAACGACCACTTTG
GAPDH-rt-R	TTGATGGTACATGACAAGGTGCGG
PAX5-rt-F	AAACCAAAGGTCGCCACAC
PAX5-rt-R	GTTGATGGAACTGACGCTAGG
AUF1-rt-F	GCGTGGGTTCTGCTTTATTACC
AUF1-rt--R	TTGCTGATATTGTTCCTTCGACA
AGO2-rt-F	TCCACCTAGACCCGACTTTGG
AGO2-rt-R	GTGTTCCACGATTTCCCTGTT
FUS-rt-F	CTATGGAACTCAGTCAACTCCCC
FUS-rt-R	CTGCCCGTAAGACGATTGG
JAK2-rt-F	TCTGGGGAGTATGTTGCAGAA
JAK2-rt-R	AGACATGGTTGGGTGGATACC
circ-MALAT1-rt-F(divergent)	AGCTGAGTGATAAAGGCTGAGTG
circ-MALAT1-rt-R(divergent)	TGATCTGGTCCATTAAAGAGTGTTC
circ-MALAT1-rt-F(convergent)	TGTCTGCGAACACTCTTT
circ-MALAT1-rt-R(convergent)	AATCTCCCACCTGTCTAA
circ-CDYL-rt-F(divergent)	CATGGCCACAGGCTTAGCTG
circ-CDYL-rt-R(divergent)	TCATAGCCTTTCCACCGAACC
circ-CDYL-rt-F(convergent)	CATCCACGACTTCAACAGA
circ-CDYL-rt-R(convergent)	CCAATCACGAGTGCCTTA
circ-TCONS_l2-rt-F(divergent)	GTATACCTGATCACCAGCAGCAG
circ-TCONS_l2-rt-R(divergent)	AGGAGCCACCTGACACCTATTC
circ-TCONS_l2-rt-F(convergent)	GGCTCCTCACCACTCTAC
circ-TCONS_l2-rt-R(convergent)	ACACCAATAACTCCGTCA
circ-LTBP2-rt-F(divergent)	TGACTACCCAGGAGGACTGCTG
circ-LTBP2-rt-R(divergent)	TCGGGTGAAGTCGGACAGTG
circ-LTBP2-rt-F(convergent)	TTCCAGGATGGCAGTTGT
circ-LTBP2-rt-R(convergent)	GGTATTGACAAGCACCAAGTAG
circ-RPPH1-rt-F(divergent)	ATGGGAGTGGAGTGACAGGAC
circ-RPPH1-rt-R(divergent)	CGGAGCTTGGAACAGACTCAC
circ-RPPH1-rt-F(convergent)	GCCGTGAGTCTGTTCCAAGC
circ-RPPH1-rt-R(convergent)	CGTCCTGTCACTCCACTCCC
circ-FKBP10-rt-F(divergent)	GATTATCATCCCTCCATTCCTG
circ-FKBP10-rt-R(divergent)	CTCACCTGCACGGTGTCTTC
circ-FKBP10-rt-F(convergent)	AAGGGCGGCACTTATGAC
circ-FKBP10-rt-R(convergent)	CCAGGAATGGAGGGATGA
circ-HIPK3-rt-F(divergent)	CCAACATGGGAAATCCAGTGAC

circ-HIPK3-rt-R(divergent)	GGTGGGTAGACCAAGACTTGTG
circ-HIPK3-rt-F(convergent)	AAGTAGAGCCAAGCAGTT
circ-HIPK3-rt-R(convergent)	CTATGACCTTTGTAGCACC
circ-COL5A1-rt-F(divergent)	GCGTCTTCCCTGACAAGAAGTC
circ-COL5A1-rt-R(divergent)	GTCCACGTAGTTCTCGCCATTC
circ-COL5A1-rt-F(convergent)	GGCATGGAAGAGATCTTC
circ-COL5A1-rt-R(convergent)	GAGCTGCAGGTCCTTGCA
circ-SPARC-rt-F(divergent)	GTACATCGCCCTGGATGAGTG
circ-SPARC-rt-R(divergent)	GCAAAGAAGTGGCAGGAAGAG
circ-SPARC-rt-F(convergent)	TACATCCCCCCTTGCCTG
circ-SPARC-rt-R(convergent)	GCTTCTCAGTCAGAAGGT
U6 snRNA-Forward	GCTTCGGCAGCACATATACTAAAAT
miR-6887-3p-Forward	TCCCCTCCACTTTCCTCCTAG
miR-214-3p-Forward	ACAGCAGGCACAGACAGGCAGT
miR-676-5p-Forward	TCTTCAACCTCAGGACTTGCA
miR-503-5p-Forward	TAGCAGCGGGAACAGTTCTGCAG
miR-4773-Forward	CAGAACAGGAGCATAGAAAGGC
miR-512-5p-Forward	CACTCAGCCTTGAGGGCACTTTC
miR-Reverse	TGCTGTCAACGATACGCTACG

Supplementary Table 10. Sequences of siRNAs and miRNAs

name	sequence（5’→3’）
siRNA NC Sense	UUCUCCGAACGUGUCACGUTT
siRNA NC Antisense	ACGUGACACGUUCGGAGAATT
siAUF1-1 Sense	GCGAAGAUUGACGCCAGUATT
siAUF1-1 Antisense	UACUGGCGUCAAUCUUCGCTT
siAUF1-2 Sense	GAAGGUGAUUGAUCCUAAATT
siAUF1-2 Antisense	UUUAGGAUCAAUCACCUUCTT
sicirc-MALAT1 Sense	UUAGAAACUUUGUCUGCGATT
sicirc-MALAT1 Antisense	UCGCAGACAAAGUUUCUAATT
siPAX5 Sense	CGCAAGAGAGACGAAGGUATT
siPAX5 Antisense	UACCUUCGUCUCUCUUGCGTT
control mimic sense	UUCUCCGAACGUGUCACGUTT
control mimic antisense	ACGUGACACGUUCGGAGAATT
miR-6887-3p mimic sense	UCCCCUCCACUUUCCUCCUAG
miR-6887-3p mimic antisense	AGGAGGAAAGUGGAGGGGAUU
miR-512-5p mimic sense	CACUCAGCCUUGAGGGCACUUUC
miR-512-5p mimic antisense	AAGUGCCCUCAAGGCUGAGUGUU
control inhibitor	CAGUACUUUUGUGUAGUACAA

miR-6887-3p inhibitor	CUAGGAGGAAAGUGGAGGGGA
miR-512-5p inhibitor	GAAAGUGCCCUCAAGGCUGAGUG

Supplementary Table 11. Sequences of probes

name	sequence（5’→3’）	modified
Control Probe	AACCGTCCATAATACTCTGTCCTTCGTAGTTCGGACCCCATT	3’-biotin
Circ-MALAT1 Probe 1	GAGTGTTCGCAGACAAAGTTTCTAAAAATACACCAGCAAAAT	3’-biotin
Circ-MALAT1 Probe 2	TTAAAGAGTGTTCGCAGACAAAGTTTCTAAAAATACACCAGCA AAATGTACT	3’-biotin
PAX5 Probe 1	TTACCCCAGGCTTGATGCTTCCTGTCTCATAATACCTGCCAA	3’-biotin
PAX5 Probe 2	AGTGGACACTATGCTGTGACTGGAAGCTGGGACTGGTT	3’-biotin

Detailed Experimental Section

Isolation of HCC Primary Cells and Cell Culture: The tumor tissues for isolation of HCC primary cells were collected from HCC patients at the Second Affiliated Hospital of Navy Medical University (Shanghai, China). HCC primary cells were prepared from patients' tumor tissues within 2 h after surgical resection by human tumor dissociation kit (Miltenyi) according to

the recommended procedure in the manual. Cells were resuspended by Dulbecco's modification of Eagle's medium (DMEM; Corning) with 10% fetal bovine serum (FBS; Gibco) for further culture. All HCC primary cells and HCC cell lines were routinely cultured in DMEM supplemented with 10% FBS at 37°C, 5% CO_2. This study was authorized by the Committee on Ethics of Biomedicine, Navy Medical University.

Tumorsphere Assays: Confluent routinely cultured monolayers of HCC cells were trypsinized and plated as single-cell suspensions (5×10^4 cells/well) onto 6-well polyHEMA (Sigma)-coated plates (Corning), and grown in DME/F-12 1:1 (Hyclone) supplemented with 20 ng/mL insulin-like growth factor (IGF; PEPROTECH), 1.0 ng/mL basic fibroblast growth factor (bFGF; PEPROTECH), and 20 ng/mL epidermal growth factor (EGF; PEPROTECH). The primary tumorspheres were counted or harvested after 5-7 d. Tumorspheres were then dissociated into single-cell suspension to be reseeded for a second round of tumorspheres formation. After 5-7 d, the secondary tumorspheres were collected and dissociated to be reseeded for a tertiary round. The tertiary tumorspheres were cultured for 5-7 d and then counted.

CircRNA-seq Analysis: Total RNA of over 20 μg per sample was purified using TRIzol reagent (Invitrogen). CircRNA-seq was then performed by RiboBio (Guangzhou, China). Briefly, total RNA was removed poly(A) RNA and rRNA and treated with RNase R before constructing RNA-seq libraries. Strand-specific RNA-seq libraries were then prepared and sequenced using IlluminaHiSeq 2500 platform. RNA-seq reads were sequentially aligned to the reference genome of human hg19 using TopHat[1] and TopHat-Fusion. The reads mapped with TopHat-Fusion but not with TopHat on the same chromosome were screened as candidate back-spliced junction

reads, and their precise positions were determined by further realigning against existing gene annotation. Mapping errors were adjusted with GT/AG. From the remaining reads circRNAs were identified using a computational pipeline according to the method described by Memczak et al.[2]. Reads mapped to the human hg19 genome and across an identified backsplice in each sample were counted, and counts were normalized by Reads Per Million mapped reads [RPM] and read length. CircRNAs differentially expressed between samples were selected by fold change (> 2 or < 0.5) and significance level ($P< 0.001$).

RNase R Treatment: Total RNA was first incubated with DNase I (Invitrogen) for 15 min at 37°C to remove poly(A) RNA and rRNA and then incubated with or without 3 U/μg of RNase R (Epicentre) for 3 h at 37°C before they were subjected to RNA extraction.

RNA Isolation, Reverse Transcription and qRT-PCR: For all samples, total RNA was purified with the TRIzol Reagent (Invitrogen). First-strand cDNA was synthesized using PrimeScript™ RT Master Mix (TaKaRa) according to the manufacturer's protocol, except that reverse transcription reaction for miRNA was used miRcute Plus miRNA First-Strand cDNA Kit (TIANGEN). For circRNAs, first-strand cDNA was synthesized without Oligo dT Primer. cDNA was prepared for qRT-PCR with SYBR® Premix Ex Taq™ (TaKaRa) in a 96-well plate with specific primers (see table S6) on a LightCycler® 480 System (Roche) following the instructions. The relative expression of RNA compared with control was calculated using the $2^{-\Delta\Delta Ct}$ method. Divergent primers were used for circRNAs-specific amplification unless otherwise indicated .

Immunocytochemistry: Tumorspheres were washed twice with 4% sucrose, and then fixed with

4% sucrose/4% formaldehyde (Thermo) for 15 min. Tumorspheres were washed three times for 5 min each time with 1X phosphate-buffered-saline (PBS; Corning) before dehydrating and paraffin-embedding, and cut to 6 μm slices. Then the slices were permeabilized with 0.2% Triton X-100 (Amresco) for 10 min, and blocked with 5% normal goat serum (Abcam) for 30 min. Slices were then incubated with primary antibody for 1 h at room temperature, washed three times for 5 min each time in 1X PBS, and incubated in the secondary antibody dilution for 1 h at room temperature. Primary antibodies used included rabbit anti-Oct4 (1: 250; Abcam) and mouse anti-AFP (1: 200; Abcam). 4,6-diamidino-2-phenylindole (DAPI) was used for nucleic acid staining (Invitrogen). Secondary antibodies to IgG of rabbit or mouse were conjugated with Alexa Fluor®488 or 594, respectively (1: 250-500; Invitrogen).

Western Blot Analysis: Total soluble proteins extracted from the samples were separated on 10% SurePAGE™ Bis-Tris gels (GenScript) and transferred electrophoretically to an Immobilon® PVDF Membrane (Millipore). Blots were blocked in 5% bovine serum albumin, followed by incubation with antibodies specific for either proteins of interest or GAPDH. Blots were then incubated with goat anti-rabbit HRP-conjugated secondary antibodies (Santa Cruz) and visualized through enhanced chemiluminescence. Primary antibodies of GAPDH, JAK2, PAX5, FGF2, TGFB1, PTK2, GH1, ACTA1, ARFIP1, CLDN5, KRT18, STAT3, STAT5a/b, phospho-JAK2, phospho-STAT3, phospho-STAT5 and p53 were purchased from Abcam. AUF1 Rabbit mAb was purchased from Cell Signaling Technology.

Construction of Overexpression Plasmids: To construct circ-MALAT1 and circ-SPARC overexpression plasmid, PCR fragments were amplified from Huh7 transcripts using primers as

follows: circc-MALAT1-forward, 5'-ATCTGTTCAATTAACGAATTCTGAAATATGCTATCTTACAGAAACTTTGTCTGCGAAC -3'; circ-MALAT1-reverse: 5'- ATCATCCCAAATTA GTGGATCCTCAAGAAAAAATATATTCACCTAAAAATACACCAGCAA -3'; circ-SPARC -forward, 5'-ATCTGTTCAATTAACGAATTCTGAAATATGCTATCTTACAGGTGTGCAGCAATGACA ACAAG -3'; circ-SPARC-reverse: 5'-ATCATCCCAAATTAGTGGATCCTCAAGAAAAAATATATTCACTCTGCTTGATGCCGA AGCA -3'. Fragments were inserted into the pLCDH-ciR vector (Geneseed) digested with restriction endonuclease EcoR1 and BamH1 (Thermo) using ClonExpress®II One Step Cloning Kit (Vazyme).

Plasmid, SiRNA and MiRNA Transfection: Circ-MALAT1 was overexpressed using custom designed plasmid as mentioned above and knocked down using custom designed siRNA oligonucleotides (RiboBio, see Table S10) with Lipofectamine 3000 (Invitrogen). AUF1 and PAX5 were knocked down using custom designed siRNA oligonucleotides (Sangon, see Table S10) with Lipofectamine 3000 (Invitrogen). 2×10^6 cells were transfected with 5 μg plasmid or 10 nM siRNA duplex or miRNA oligonucleotides following the manufacturer's protocol. For stable overexpression of circ-MALAT1, lentivirus was produced using three-plasmid packaging system. 293T cells were co-transfected with the plasmid of interest and packaging plasmids pMD2G and psPAX2 by calcium phosphate transfection. Infectious lentiviruses were harvested at 48 h after transfection.

Cell Proliferation, Cell Cycle and Invasion Assays: For cell proliferation assay, 1×10^4 Hep3B or 5×10^3 Huh7 cells per well were plated into 96-well flat-bottomed plates with 100 μL of cell suspension. Every 24 h, cell viability was measured with Cell Counting Kit-8 (Dojindo), and each experiment with six replicates was repeated three times. EdU incorporation assays were performed using the Click-iT™ EdU cell proliferation Assay Kit (Invitrogen). Before beginning, HCC cells were seeded in the appropriate amount such that they would be 60-70% confluent at the time of incubation with 2 μM EdU. At last, the stained sections were examined using a fluorescence microscope and cell numbers in at least three random fields were counted. For cell cycle analysis, HCC cells were prepared as single cell suspensions before being fixed in 70% ethanol at 4°C overnight. The cells were labeled with propidium iodide (Sigma) and analyzed through flow cytometry (BD Biosciences). Transwell assays were carried out in Millicell chambers in triplicate. 5-10×10^4 cells were added to the upper coated filters in serum-free DMEM, and the lower chambers were supplemented with cell-free DMEM containing 10% FBS as a chemoattractant. After 12-16 h culture, cells that migrated through the filters to the bottom were fixed and stained. Cell numbers in at least three random fields were counted.

Protein Microarrays: Differential expressions of proteins between circ-MALAT1-overexpressed and empty vector-transfected Huh7 cells were measured using H-Wayen Biotin Label-based Human Antibody Array (#SET100), according to recommended procedures. These arrays could detect 1358 proteins. Hybridization was done at room temperature for 2 h. All slides were scanned using Axon's GenePix 4000B (Axon Instruments) scanner and analyzed using GenePix Pro 6.0. F532 Median-B532 score on each array was averaged across triplicates. Internal controls were used to normalize the results and values of vector controls were subtracted. All results were

available in Table S2.

RNA Fluorescence *in situ* Hybridization: Hep3B cells were fixed at 70-80% confluence in the exponential growth phase with 4% paraformaldehyde and then permeabilized in 0.5% Triton X-100. After 30 min of pre-hybridization at 37°C, the cells were hybridized with DIG-labelled probes (DIG 5'- GCAGACAAAGTTTCTAAAATACACCAGCA -3' DIG) specific to circ-MALAT1 at 37°C overnight. Signals were measured using Alexa Fluor™ 488 Tyramide Reagent (Invitrogen). Nuclei were counterstained with DAPI.

Prediction of MiRNAs Related to CircRNAs: The sequences and annotations of published miRNAs were obtained via miRBase database (www.mirbase.org). The miRNAs with high confidence in humans were selected. The circRNA-miRNA interaction networks were predicted using miRanda pipeline[3]. Match score was set above 170 and minimum free energy was less than -30 to improve reliability of our prediction.

Dual Luciferase Reporter Assay: The 3'UTR of human Janus kinase 2 (JAK2) containing miR-6887-3p target sites was cloned using the following primers: forward, 5'-GACTAGTCACATGAGGGCTGGTGTTCA -3'; reverse, 5'-GGTTTAAACTGACAACAACGAACAACCCC -3'. The fragment was then inserted into the pMIR-REPORT vector to generate a wild-type pMIR-JAK2-3'UTR reporter. A mutated pMIR-JAK2-3'UTR plasmid was cloned with substitution of miR-6887-3p target site. Both were verified by sequencing. HEK293T cells were co-transfected with wild type or mutant constructs, scrambled mimic (or inhibitor) or miR-6887-3p mimic (or inhibitor) and pRL-TK using

Lipofectamine 3000 (Invitrogen). The cells were collected for detecting luciferase activity at 24 h post transfection using the Dual Luciferase Reporter Assay Kit (Promega). The luciferase activity was measured using the Multiscan Spectrum (PerkinElmer). miRNA mimics and inhibitors were synthesized by Sangon Biotech. The miRNA sequences were shown in Table S10.

RNAs *in vivo* Pull Down: The following assay was performed referring to the protocols of circRNAs *in vivo* precipitation (circRIP) by Han et al.[4] with minor modifications. Circ-MALAT1-overexpressing Huh7 cells were prepared in advance and then transfected with the biotin-tagged specific probes or control probe (Sangon, sequences shown in Table S11) at a final concentration of 0.2 μM at ~70% confluency. Cells were incubated at 37°C and 5% CO_2 for 24 h. The next day, after thoroughly homogenized by washing twice with Solution A and once with Solution B, 100 μL of M-280 Streptavidin Dynabeads (Invitogen) suspension per sample was blocked for 3 h. Then, the beads mixture was washed twice in a lysis buffer and ready to be used. During blocking, cells transfected with the probes were fixed by 1% formaldehyde for 10 min, equilibrated by glycine solution for 5 min and washed with ice-cold PBS three times. After that, cell samples were resuspended with 1 mL lysis buffer, followed by sonication (50% amplitude, 10 s constant pulse, and 10 s pause) for 10 min and centrifugation at 12000 rpm for 10 min at room temperature. The supernatant was then allowed to interact with the prepared M-280 dynabeads, followed by incubation overnight with rotating at room temperature. On day 3, the resultant dynabeads-probes-RNAs mixture was washed with wash buffer A and wash buffer B twice, and resuspended in 500 μL TRIzol (Invitrogen) and subjected to RNA purification.

RNA-binding Protein Immunoprecipitation (RIP): RIP experiment was performed with the

Millipore universal RIP immunoprecipitation kit (Millipore) according to the manufacturer's guidelines. Briefly, circ-MALAT1-overexpressing Huh7 cells were harvested at ~90% confluency. Cell samples was resuspended and homogenized thoroughly in complete RIP lysis buffer, incubated on ice, and centrifuged at 4°C. Before cells lysis, antibodies of interest (anti-AUF1 (CST), anti-RPS6 (Abcam)) or Rabbit IgG were added to the beads suspension and incubated with rotation for 30 min at room temperature. The beads-antibody complex was then washed and resuspended in the RIP immunoprecipitation buffer. 5% of the cell supernatant was transferred into a new tube as an input and stored at -80°C until RNA purification was started. The remaining supernatant was split equally into each beads-antibody complex in the RIP immunoprecipitation buffer and incubated with rotating at 4°C overnight. Next day, each immunoprecipitation tube was washed completely with the RIP wash buffer, resuspended in the protein K buffer and incubated with shaking for 30 min at 55°C to digest the proteins. After incubation and magnetic separation, the supernatant was transferred into a new RNase-free tube and subjected to RNA isolation.

Tumor Xenograft Model: Male BALB/c nude mice without specific pathogens of 4-5 weeks were purchased from the Charles River Laboratories (Beijing, China), and maintained under a specific pathogen-free condition. After about 1 week, 2×10^6 HCC cells resuspended in DMEM were subcutaneously inoculated into the dorsal flank of a nude mouse (day 0). One month later, the mice were dislocated and the subcutaneous tumors were removed and measured. To verify the miRNA regulatory mechanism *in vivo*, miRNA mimic- or inhibitor-overexpressing lentivirus was injected intratumorally into mice bearing established tumors on day 14, once weekly for two weeks. After two weeks, the mice were sacrificed, and subcutaneous tumors were analyzed. All

experiments involving mice were conducted in accordance with the guidelines for animal welfare formulated by the laboratory animal center at Navy Medical University.

Statistical Analysis: Statistical analysis was performed by GraphPad Prism 7.0. All numerical data were presented as mean±SD for multiple samples with at least three replicates. Differences between groups were evaluated by the two-tailed Student's *t*-test. *P* values less than 0.05 were considered significant, indicated by an asterisk (*), and below 0.01 were considered extremely significant, indicated by two asterisks (**).

REFERENCES

[1] C. Trapnell, L. Pachter, S.L. Salzberg, *Bioinformatics* **2009**, *25*, 1105.

[2] S. Memczak, M. Jens, A. Elefsinioti, F. Torti, J. Krueger, A. Rybak, L. Maier, S.D. Mackowiak, L.H. Gregersen, M. Munschauer, A. Loewer, U. Ziebold, M. Landthaler, C. Kocks, F. le Noble, N. Rajewsky, *Nature* **2013**, *495*, 333.

[3] D. Betel, A. Koppal, P. Agius, C. Sander, C. Leslie, *Genome Biol* **2010**, *11*, R90.

[4] D. Han, J. Li, H. Wang, X. Su, J. Hou, Y. Gu, C. Qian, Y. Lin, X. Liu, M. Huang, N. Li, W. Zhou, Y. Yu, X. Cao, *Hepatology* **2017**, *66*, 1151.
